# Integrated Science Teaching in Atmospheric Ice Nucleation Research: Immersion Freezing Experiments

**DOI:** 10.1021/acs.jchemed.2c01060

**Published:** 2023-03-08

**Authors:** Elise K. Wilbourn, Sarah Alrimaly, Holly Williams, Jacob Hurst, Gregory P. McGovern, Todd A. Anderson, Naruki Hiranuma

**Affiliations:** † Department of Life, Earth, and Environmental Sciences, West Texas A&M University, Canyon, Texas 79016, United States; ‡ Department of Chemistry and Physics, West Texas A&M University, Canyon, Texas 79016, United States; § Department of Environmental Toxicology, Texas Tech University, Lubbock, Texas 79416, United States

**Keywords:** First-Year Undergraduate/General, Upper-Division Undergraduate, Graduate Education/Research, Environmental Chemistry, Hands-On Learning/Manipulatives, Collaborative/Cooperative Learning, Atmospheric Chemistry

## Abstract

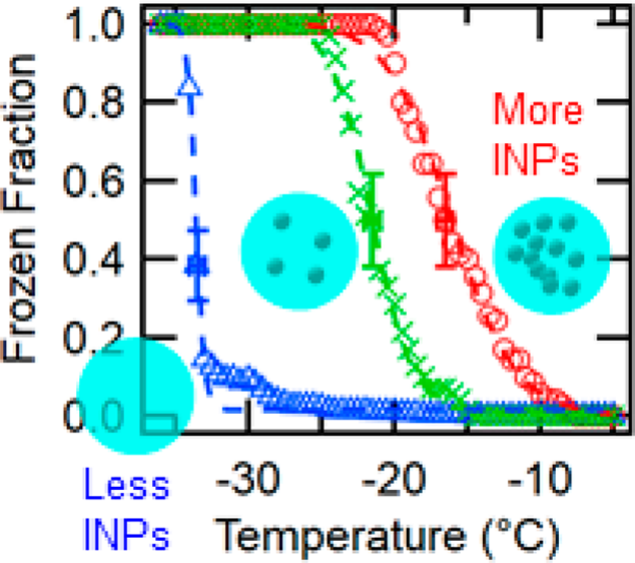

This paper introduces hands-on curricular modules integrated with research in atmospheric ice nucleation, which is an important phenomenon potentially influencing global climate change. The primary goal of this work is to promote meaningful laboratory exercises to enhance the competence of students in the fields of science, technology, engineering, and math (STEM) by applying an appropriate methodology to laboratory ice nucleation measurements. To achieve this goal, three laboratory modules were developed with 18 STEM interns and tested by 28 students in a classroom setting. Students were trained to experimentally simulate atmospheric ice nucleation and cloud droplet freezing. For practical training, this work utilized a simple freezing assay device called the West Texas Cryogenic Refrigerator Applied to Freezing Test (WT-CRAFT) system. More specifically, students were provided with hands-on lessons to calibrate WT-CRAFT with deionized water and apply analytical techniques to understand the physicochemical properties of bulk water and droplet freezing. All procedures to implement the developed modules were typewritten during this process, and shareable read-ahead exploration materials were developed and compiled as a curricular product. Additionally, students conducted complementary analyses to identify possible catalysts of heterogeneous freezing in the water. The water analyses included: pH, conductivity, surface tension, and electron microscopy–energy-dispersive X-ray spectroscopy. During the data and image analysis process, students learned how to analyze droplet freezing spectra as a function of temperature, screen and interpret the data, perform uncertainty analyses, and estimate ice nucleation efficiency using computer programs. Based on the formal program assessment of learning outcomes and direct (yet deidentified) student feedback, we broadly achieved our goals to (1) improve their problem-solving skills by combining multidisciplinary science and math skills and (2) disseminate data and results with variability and uncertainty. The developed modules can be applied at any institute to advance undergraduate and graduate curricula in environmental science.

## 1 Introduction

Atmospheric ice-nucleating particles (INPs) are a subset of aerosol particles that promote the heterogeneous formation of ice crystals under ice supersaturation conditions. According to a recent modeling simulation study, more than 85% of INPs are activated to ice crystals in tropospheric clouds through so-called immersion freezing, which refers to ice nucleation of cloud droplets in the presence of INP.^1^ On a planetary scale, INPs contribute to the partitioning between ice and liquid water in boundary layer clouds, influencing their albedo and climate sensitivity.^2^ Recently, Murray et al. (2021) postulated potential climatic feedback through increases in the atmospheric INP concentration in response to climate change.^3^ Briefly, the authors proposed a mechanism of increasing ambient INP concentration and emission from bare geological surfaces in part due to decreased snow and ice coverage. Because INPs can catalyze precipitation and act as cloud-destroying agents, the increase in INPs may result in accelerated positive radiative feedback.

To date, aerosol radiative forcing remains highly uncertain, and climate feedback mechanisms associated with INPs and clouds are not well understood. In particular, the understanding of atmospheric ice formation in mixed-phase clouds, where supercooled water droplets and ice crystals coexist, represents a major challenge and motivates the community’s current interest in quantifying and improving predictive skill for INP number concentrations.^4^ Aerosol–cloud interactions through INPs are not well represented in most CMIP6 (Coupled Model Intercomparison Project Phase 6) models. The intermodel spread is so large that model predictions of effective radiative forcing from aerosol–cloud interactions even differ in sign according to the most recent Intergovernmental Panel on Climate Change Report (i.e., Chapter 7).^5^

Ambient INP abundance, sources (e.g., dust, sea spray, and biogenic), and their fundamental properties remain poorly quantified despite recent efforts.^6−8^ In general, immersion-active INP concentrations can span 2–3 orders of magnitude at a given temperature or 10 orders of magnitude, ranging from 10^–6^ to 10^4^ L^–1^, in both continental and marine-predominant sites across the world at temperatures above about −35 °C (See Supporting Information, SI, Sect. S1, Freezing of Water Droplets and Ice-nucleating Particles Figures 2 and 4). However, how fundamental physicochemical properties of aerosol particles introduce such a diverse INP concentration range remains uncertain.

Droplet freezing assays are a common practice in atmospheric INP research to measure the immersion freezing abilities of INPs as a function of temperature in a controlled setting. This technique has been widely applied to assess freezing properties of various sample types, including suspended dry powders and filter-collected ambient particle suspensions, from different environments.^9−11^ The reproducibility of this assay is dependent on exclusion of background freezing artifacts.^12^ Additionally, substrate surfaces, variation in droplet size, and experimental variables can impact the ability to determine homogeneous freezing temperature, which is typically below ≈−35 °C.^13^ An exception is found in deionized (DI) nanoliter water droplets, which facilitate a result that follows homogeneous freezing according to Classical Nucleation Theory.^14^ However, such a rigorous nanoscale droplet technique is not commonly available to researchers.

Providing hands-on opportunities for STEM (science, technology, engineering, and math) students and junior scientists to apply affordable INP measurement technologies is one of the key aspects to fill the knowledge gap in the physicochemical properties of INPs. Hence, this paper offers integrated science curricular teaching and fundamental laboratory modules/lessons examining and understanding heterogeneous and homogeneous freezing (i.e., triggered by INPs or without INPs, respectively) of suspension samples to engage college students (at any level) in the diverse and rich science of atmospheric ice nucleation.

## 2 Method

The presented curricular training was developed to educate bulk water sample characterization techniques (pH, conductivity, and surface tension), an immersion freezing assay, and chemical composition analysis. In Sects. 2.1 to 2.3, individual techniques are described along with associated procedures. A set of three written curricular module instructions is available in SI Sect. S1. Outcomes of the student-participating modules were assessed for each module, and the assessment procedure and intended learning outcomes are described in Sect. 2.4 and SI Sect. S2.

### 2.1 Module 1: Water Samples and Characterization of Their Bulk Properties

The module instructors and three intern students preassessed three water samples and codeveloped the model results, as well as curricular Module 1 shown in SI Sect. S1. Afterward, four water samples were examined by 18 students in this curriculum in the classroom setting. In class, all students spent time in one-on-one or one-on-two training with the module instructors or teaching assistant prior to being exposed to the module.

Instructors purchased commercially available high-performance liquid chromatography (HPLC)-grade water (Sigma-Aldrich, 270733-20L), and the students used it as their pure water standard. The tap water sample was collected in West Texas on 3/24/2021. The filtered tap water sample was prepared by filtering the tap water through a sterile syringe filter connected to a sterile 25 mm diameter polycarbonate filter with 0.2 μm pore size (VWR, 28145-477 and 309653). Additionally, DI water was examined in the classroom setting. The HPLC water sample was kept in a dry and cool lab, and a 1 gallon sample for each tap water type was stocked in a chemically inert high-density polyethylene bottle (VWR, 414004-159) and stored at −80 °C until analyzed. An exception was during the transportation (no more than 3 h), but all samples were kept frozen in a cooling box during this time with ice. It should be noted that the same individual samples from each stock were used for all subsequent analyses.

The surface tension of all water samples was measured by using a tensiometer (DWK Life Science, Model 14818). Briefly, the employed surface tension analyzer consists of a glass capillary tube (0.5 mm nominal inner diameter) and an outer glass cylinder with tubulation covered by a rubber cap. As surface tension leads to the phenomena of suspension capillary rise or depression, positive (or negative) pressure was purposely introduced into the tensiometer to estimate the surface tension based on the measurement of capillary depression or rise (See SI Sect. S3). More specifically, in our experiment, we first drew air out of the cylinder until we observed that air bubbles were pulled out of the capillary. Then, the syringe was removed from the tubing to allow the sample liquid to reach equilibrium inside the capillary tube. Finally, the distance was measured between the meniscus inside the capillary tube and the meniscus of the cylinder. This process was repeated three times, followed by creating a positive pressure inside the capillary tube to pull liquid from the top of the tube. The distance between the meniscus inside the tube and the meniscus inside the cylinder was again measured after the syringe was disconnected. This process was also repeated three times, and the average was taken and used to calculate the surface tension using Eqn. S1, provided by DWK LifeScience.

An Oakton pH/conductivity probe (Waterproof pH/Con 10 m) was used for pH and conductivity analyses of all water samples. The probe was calibrated with liquid pH and conductivity standards (pH of 4.01, 7.00, and 10.01 as well as conductivities of 23, 100, 447, and 2764 μS, purchased from VWR). The manufacturer-reported systematic error of the probe is ±0.01 pH and ±1% conductivity. The probe tip was kept moist with DI water until analysis was completed.

### 2.2 Module 2: Immersion Freezing Assessment

To assess water droplet freezing efficiency, 12 interns and 5 in-class students used an offline droplet-freezing assay instrument, West Texas Cryogenic Refrigerator Applied to Freezing Test (WT-CRAFT).^15^ The module lesson plan as read-ahead exploration material was prepared by the module instructors and 12 interns (Module 2 in SI Sect. S1) to introduce the students to the scientific concepts. Additionally, the instructors and teaching assistant provided a guide to the students to optimize the measurement conditions and subsequent data analysis processes. This strategy helped to develop the student-centered curriculum and also ensure that the students have an understanding of the science that they convey via prefabricated modules. Further, laboratory training by an instructor or teaching assistant was provided for all students in a one-to-one fashion to use the instrument.

The WT-CRAFT system is a replica of Cryogenic Refrigerator Applied to Freezing Test (CRAFT).^16^ Two students from West Texas A&M University visited the National Institute of Polar Research (NIPR) in Japan to learn the operation of CRAFT and replicate the system at West Texas A&M University. Currently, the measurement sensitivities, variables, and detection limits of WT-CRAFT differ from the original CRAFT system as described in Vepuri et al. (2021).^15^ Briefly, WT-CRAFT enables a simulation of atmospheric immersion freezing using supercooled droplets in the subzero temperature range. The interns and students evaluated 70 solution droplets (3 μL each) placed on a hydrophobic petroleum jelly layer with a cooling rate of 1 °C min^–1^. Each freezing event can be determined optically based on the change in droplet brightness when the initially transparent liquid droplets became opaque upon freezing. If the freezing temperature was not obvious for any droplets, the 8-bit greyscale images were assessed using ImageJ software to determine the temperature of the phase change.^17^

After a set of freezing measurements for multiple water types, the students calculated the frozen fraction (*FF*), which represents the number of frozen droplets at a given temperature, *n*_frozen_(*T*), scaled to a total number of examined droplets in a single experiment (*n* = 70) for every 0.5 °C. While the previously reported investigable temperature with negligible artifacts is −25 °C for WT-CRAFT,^15^ they examined each sample type until they observed *FF* of 1 in this curricular work. They further analyzed the data through sigmoidal curve fittings to find a 50% frozen fraction (*FF*_50_) and corresponding temperature (*T*_*FF*__50_) for each water type. The systematic uncertainties in WT-CRAFT with respect to temperature and freezing efficiency are ±0.5 °C and ±23.5%, respectively.^18^ Further, standard deviations, standard errors (i.e., standard deviations divided by square root of the number of observations), and/or the 95% confidence intervals (CI95%) of freezing measurements (statistical uncertainties) were estimated and compared to the systematic errors.

One-on-one or one-on-two training with the module instructors or teaching assistant is essential for motivating students to connect with the diverse fields of atmospheric science and contemporary climate science and to train their problem-solving and hypothesis formulation skills. To develop intuitive and student-centered curricular activities, the instructors and interns documented a written protocol of their WT-CRAFT analysis to create instructional materials, including a step-by-step operational instruction (SI Sect. S1), data (SI Sects. S4), and publicly shared video (https://doi.pangaea.de/10.1594/PANGAEA.952536). While testing the curriculum modules, the instructors kept experimental procedures and planning open and flexible and discussed the next steps together with their interns weekly.

It is worth noting that, before the students performed the module lesson in class, the instructors and intern students examined the comparability of their WT-CRAFT immersion freezing results using these off-the-shelf materials to previous studies in terms of their ice nucleation efficiencies to confirm experimental processes and uncertainties. The calibration procedure and results are discussed in SI Sect. S5.

### 2.3 Module 3: Offline Composition Analysis

The instructors trained three interns and five students in class in a one-to-one fashion to use a scanning electron microscope equipped with an energy-dispersive X-ray spectroscopy function (SEM-EDX) to assess the elemental composition of residuals in their water samples.

Complementary operation instructions for SEM-EDX (SI Sect. S1 Module 3) were provided by instrument mentors onsite or in virtual video meetings. This interaction created educational opportunities for the student to input and output scientific information through critical listening and presenting scientific progress. Due to limited instrument time and resources for this pilot study, the students focused on comparing filtered tap water vs HPLC water for SEM-EDX.

To specify the elemental composition of residual materials in water, SEM-EDX was conducted for several water samples. More specifically, to gain knowledge of nonvolatile constituents of the examined water samples, the students imaged the generated precipitates under SEM-EDX (JEOL, JSM-6010LA) and characterized their elemental compositions on a particle-by-particle basis. A consistent electron beam intensity of 20 kV was used to investigate the atomic percentage (Atomic %) abundance of 14 elements; C, N, O, Na, Mg, Si, P, S, Cl, K, Ca, Mn, Fe, and Zn. The students were instructed by the instructors to exclude the background signal of aluminum from the substrate, which was used for SEM-EDX. The weight percentage of each element was first estimated by counting characteristic X-rays emitted by measured elements via a replacement of electrons that typically happens in the K-shell electron orbital upon an interaction between the incoming electron beam and the specimen (see the Introduction section of SI Sect. S1 Module 3).

The Atomic % value represents the number of atoms of that element, at that weight percentage, divided by the total number of atoms in the sample. Thus, we initially calculated atomic proportion, which is a ratio of each element weight percentage (i.e., the weight of that element measured in the sample divided by the weight of all measured elements in the sample multiplied by 100) to its atomic weight [*atomic proportion = element weight %/atomic weight*]. By estimating this for all elements in the sample, a list of atomic proportions was obtained. Then, we summed these together to obtain a total atomic weight proportion [*TAWP = ∑ atomic proportion*]. Atomic % for each element in the sample by dividing its atomic proportion was obtained by TAWP [*Atomic % = atomic proportion/TAWP*]. An example of the atomic % calculation is provided in Sect. S1 Module 3, Introduction.

To prepare the samples for SEM-EDX, a small amount of each water sample (10 mL) from a sample stock bottle was placed on an aluminum foil container, which was precleaned with 70% reagent alcohol (VWR, BDH1164-4LP), and evaporated on a heating stage to extract nonvolatile residuals. Subsequently, the bottom section of the aluminum foil container (∼8 mm × 8 mm) was cut and kept in a Petri dish until analyzed for the elemental composition of residual precipitates.

Using SEM-EDX, the interns and in-class students assessed 257 and 131 particles, respectively, on aluminum substrates for their elemental composition. All particles had an area equivalent diameter smaller than 10 μm, and the lower bound of the particle diameter, which was resolved by SEM, was 0.3 μm. It should be noted that, because volatile and semivolatile components were presumably evaporated during our sample preparation for SEM-EDX, our precipitate composition results may not reflect the composition of INPs analyzed by WT-CRAFT for immersion freezing. This point should be cautiously kept in mind when interpreting our SEM-EDX results.

### 2.4 Assessment of Modules and Intended Learning Outcomes

The modules were assessed in the classroom setting using the end-of-course survey data collected by a third-party university contractor. These data are useful to identify typical problems that the students encounter in preparation and during exercise. This information might help faculty members who might, want to adopt this experiment at their institute. The survey and assessment results are typically anonymously gathered and digitized without student identifiers before being delivered to instructors. Further, the collected data cannot be associated with specific parties (i.e., reidentified). The University Institutional Review Board classified the data as fitting the “Exempt” category and appropriate for use in this discussion.

The classroom assessment data represent averages of the class performances on specific learning outcomes (LOs) for relevant classes. The assessment results were used to review the perceptions and engagement of the students for each module developed in this study. The two learning outcomes used for the assessment of our laboratory modules were

- LO 1: Students should be able to use integrated multidisciplinary science and math skills to address complex and emerging geoscience and environmental issues.
- LO 2: Students should be able to effectively explain research data and outcomes (graphs, tables, modeling results) with statistical defensibility

Table 1 shows the list of competency and exercise questions used to assess students’ learning outcomes for each module. The data were used to assess a positive impact on teaching and learning. Table 2 summarizes the courses in which the module and survey were given.

**Table 1:**
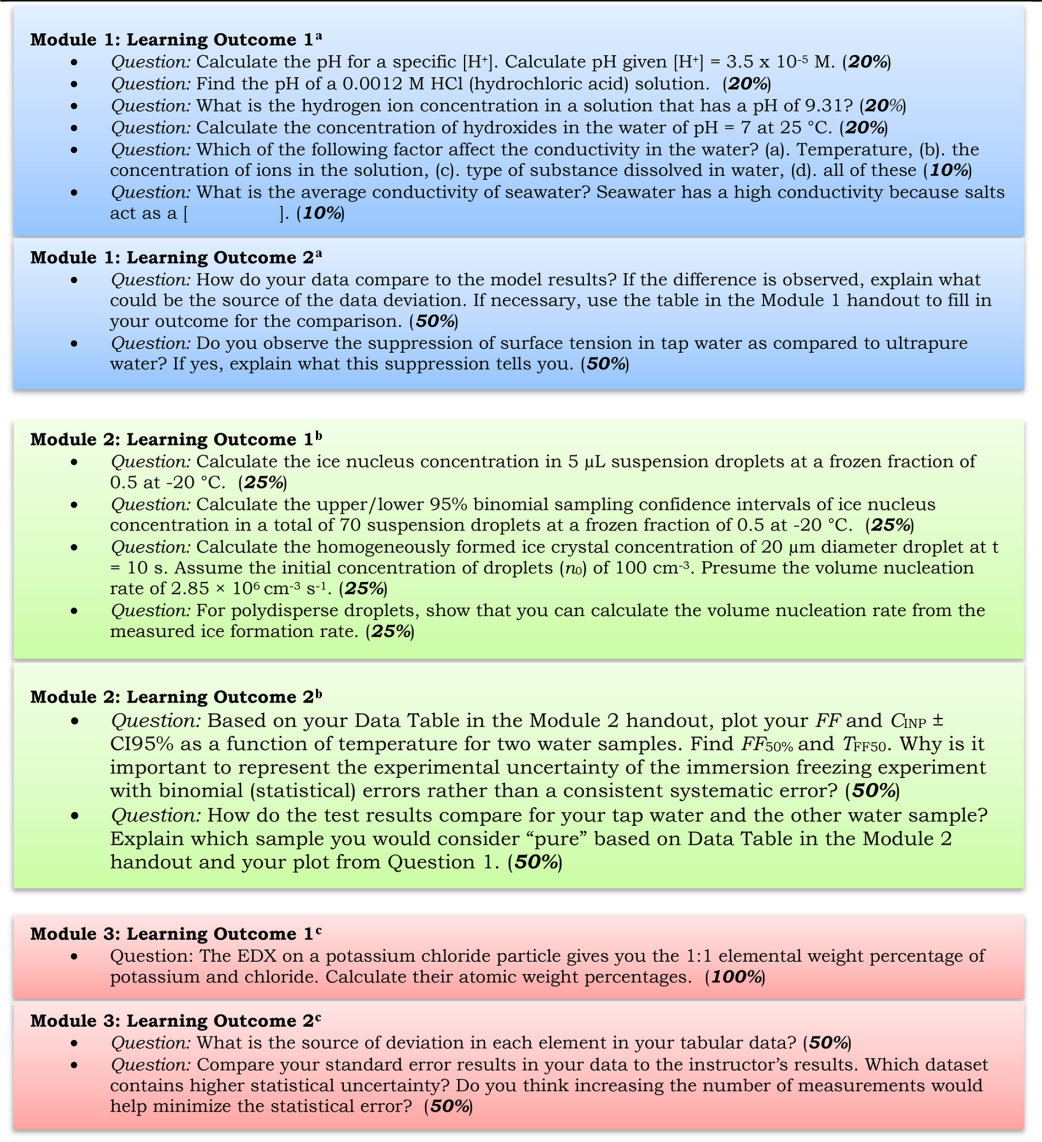
List of Competency and Exercise Questions for the Module Assessment and Grading Allocation (%)

a Model answers are available in SI Sect. 1 Module 1.

b Model answers are available in SI Sect. 1 Module 2.

c Model answers are available in SI Sect. 1 Module 3.

**Table 2:** Three Lab Modules for Different Academic Level Categories

Course	Target	Module
*Fundamentals of Environmental Science* (ENVR1407)	freshman–sophomore	Characterization of bulk water properties
*Advanced Numerical Analysis* (ENVR6303)	upper-level undergraduate or graduate-level course in Environmental Science/Chemistry	Freezing of water droplets and ice-nucleating particles
*Earth and Atmospheric Chemistry* (ENVR6092)	upper-level undergraduate or graduate-level course in Environmental Science/Chemistry	Elemental composition analysis of water residual particles by SEM-EDX

## 3 Results and Discussion

### 3.1 Bulk Properties of Water Samples

Shown in Table 3 is a summary of the model bulk analysis results for each water sample. For each sample type, three sample replicates (140 mL each) were subsequently examined for pH, conductivity, and surface tension at a stable water temperature condition (∼19 °C). In the classroom setting, students computed the average and standard deviation of these measurements. This process was important for training students to understand basic scientific uncertainties in multiple replicates.

**Table 3:** Bulk Water Properties: Average ± Standard Deviation of Three Replicated Measurements

	pH	Conductivity (μS cm^–1^)	Surface Tension (dyn cm^–1^)	*T* (°C)
Unfiltered tap water	7.5 ± 0.1	859.8 ± 23.7	73.8 ± 0.5	19.3 ± 0.9
Filtered tap water	8.0 ± 0.3	1006.3 ± 47.6	73.0 ± 0.7	18.7 ± 1.5
HPLC water	5.6 ± 0.3	22.4 ± 16.4	75.9 ± 2.1	18.9 ± 1.1

While the data may vary depending on sampling location of tap water and associated water hardness (i.e., Briggs and Ficke, 1977; concentrations of minerals, such as calcium ions, Ca^2+^, magnesium ions, Mg^2+^, hydrogen carbonate ions, HCO_3_^–^ and carbonate ions, CO_3_^2–^),^19^ the examined tap water samples used in this work exhibited higher pH and conductivity than the HPLC water. As pH is computed as a negative log value of the hydrogen ion concentration in water, [H^+^], students at any location should find a positive correlation between pH and conductivity. In other words, the abundance of mineral ions (a.k.a., impurities) is higher in tap water samples, coinciding with high ionic conductivity and relatively low [H^+^] (therefore high pH) for the given volume. On the other hand, the relative abundance of [H^+^] is the highest in the HPLC water and thereby it has the lowest pH among three samples. The reason why filtered tap water has higher conductivity and pH than unfiltered tap water is unknown. This may be due to higher concentrations of conductive ions dissolved (<0.2 μm physical size) in the filtered tap water sample assessed in this study. Regardless, according to general water quality guidelines, the tap water samples are categorized as consumable water by humans (MRCCC; <2500 μS cm^–1^).^20^

The measured surface tension of the water samples consistently shows ∼75 dyn cm^–1^. The typical surface tension of distilled water is ∼72 dyn cm^–1^ at 25 °C.^21^ The slightly lower surface tension of tap water samples may be indicative of the presence of a small concentration of surfactants. It was tested and confirmed that adding 0.5 g of sodium dodecyl sulfate (Sigma-Aldrich, 436143–25G), used as a surrogate surfactant, lowers the surface tension of our samples to <37 dyn cm^–1^. Thus, the validity of the tensiometer can be demonstrated by introducing surfactants.

### 3.2 Freezing Properties of Calibrator-Surrogates and Water Samples

**Figure 1:**
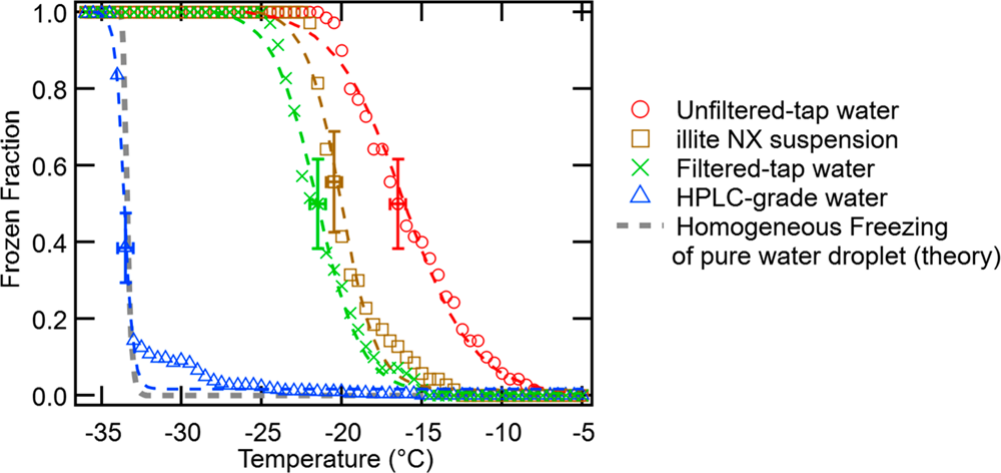
Model *FF*(*T*) spectra of HPLC-grade pure water droplets (blue triangles), tap water (red circles), filtered tap water (green cross), and Illite NX suspension (brown squares, 0.1 wt % suspended in HPLC water). Color dashed lines represent fit curves (*r* > 0.97). The conceptual spectrum of a 3 μL pure water droplet based on the classical nucleation theory is superposed on the measured spectra.^13^ Error bars indicate systematic measurement uncertainties.

Figure 1 displays the model outcome of frozen fractions of individual water samples. To guide the reader’s eye, a freezing spectrum of 0.1 wt % Illite NX suspension is added as a reference curve of heterogeneous freezing in this figure.

Using a sigmoidal fit (eq 1), students first evaluated *T*_*FF*__50_ for each sample type.
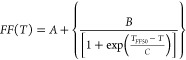
1in which *A* and *B* are the dimensionless max–min domains for the fit and *C* is the rate of temperature change (°C). Shown in Table 4 is a summary of the computed fit parameters with Pearson correlation coefficients (*r*). It should be noted that the *FF* spectrum represents a cumulative distribution function of frozen droplets as a function of temperature. Therefore, it can be converted to a probability density function with a standard deviation, σ (i.e., ), to conduct statistical uncertainty analysis for assigned temperature steps.

**Table 4:** Summary of Sigmoidal Fit Parameters^a^

	A	B	*T*_*FF*__50_ (°C)	*C* (°C)	*r*
Tap	1.011 ± 0.005	–1.011 ± 0.008	–16.136 ± 0.080	2.199 ± 0.070	0.984
Illite NX	1.008 ± 0.005	–0.993 ± 0.008	–20.085 ± 0.064	1.110 ± 0.056	0.989
Filtered tap	1.009 ± 0.005	–1.004 ± 0.006	–21.568 ± 0.055	1.394 ± 0.047	0.978
HPLC	1.001 ± 0.008	–0.984 ± 0.009	–33.597 ± 0.028	0.284 ± 0.024	0.977

a All coefficients are reported ± one standard deviation based on the given correlation coefficients (see Eqn. 1).

As seen in the figure, there is a notable gap between the water samples for their freezing spectra. Briefly, the *T*_*FF*__50_ values for the HPLC water, filtered tap water, and untreated tap water are approximately −33.6 °C, −21.6 °C, and −16.1 °C, respectively. The observed difference might represent different amounts of possible catalysts that can act as INPs in the three water samples. Such impurities may shift the *FF*_50_ point of the tap water toward a higher temperature, resulting in the observed *T*_*FF*__50_ gap between the sample types (Figure 1).

It is surprising to observe that the nonfiltered tap water contains more INPs than the Illite NX suspension, which has *T*_*FF*__50_ of −20.1 °C. Illite NX is composed of aluminosilicate and other dust components which are known to be ice nucleation active,^22^ and its freezing spectrum is similar to filtered tap water. The difference between *T*_*FF*__50_ of original tap water and that of filtered tap water might represent the amount of filterable INPs removed with 0.2 μm pore size filter.

**Figure 2:**
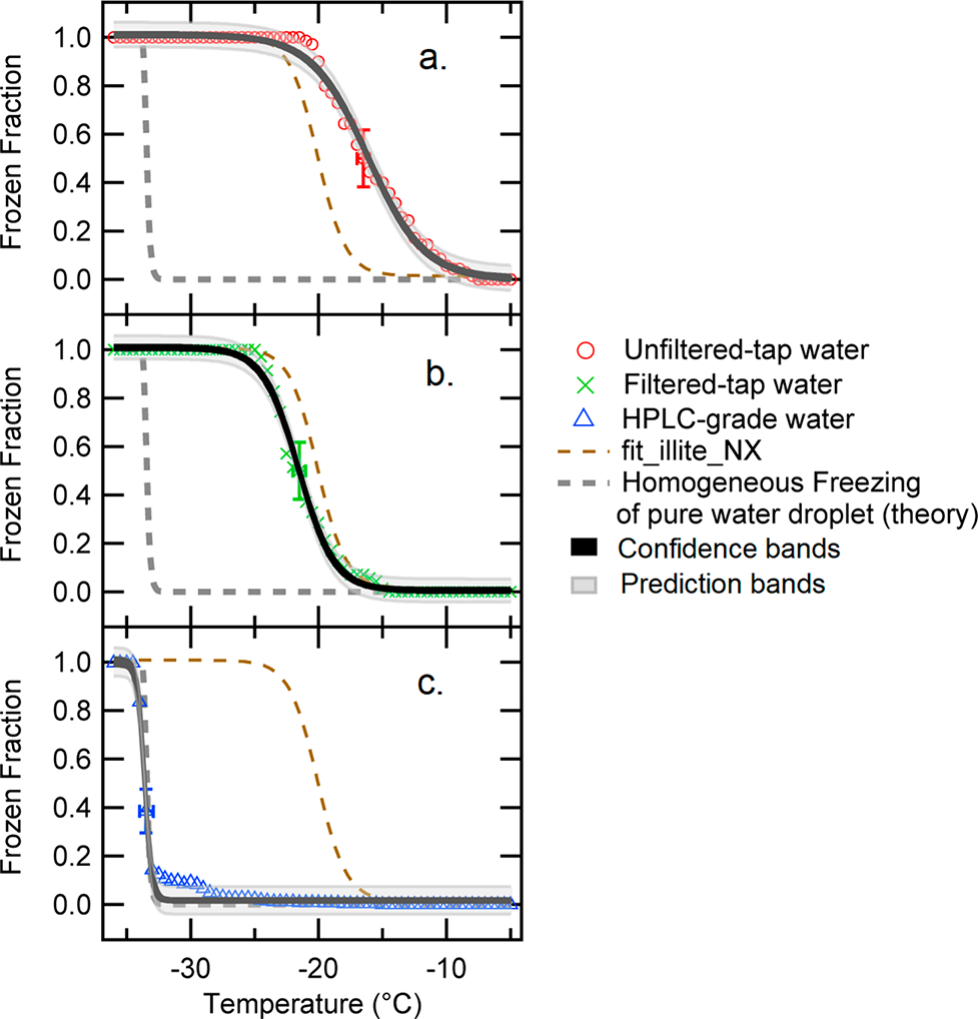
*FF*(*T*) spectra of tap water (a), filtered tap water (b), and HPLC-grade pure water droplets (c) with the upper-lower confidence bands (black shaded area) and prediction bands (gray shaded area). Two reference freezing spectra (i.e., Illite NX suspension and homogeneous freezing of a 3 μL pure water droplet) are adapted from Figure 1 and shown in dashed lines. Color dashed lines represent fit curves (*r* > 0.97). Error bars at *FF*_50_ indicate systematic measurement uncertainties.

In addition, the instructors asked their interns to apply confidence and prediction bands to estimate data uncertainty related to CI95.^23,24^ The results of the *FF*(*T*) spectra with both statistical and systematic uncertainties for each water type are shown in Figure 2. In general, a prediction band is subject to noise. In fact, the estimated prediction bands show larger uncertainties than the confidence bands at CI95 without exception. The systematic error of the employed immersion freezing assay at *FF*_50_ represents even larger uncertainties for each water type. Thus, at *FF*_50_, the statistical errors are within systematic error, validating the system performance. Most importantly, through this error analysis, students verify that all water types have unique freezing properties beyond their measurement uncertainties.

### 3.3 Artifact Composition

Following the bulk and freezing analyses, interns conducted composition characterizations of the same water samples to reveal possible INPs. Some impurities were identified in their samples through SEM-EDX. In particular, a non-negligible amount of carbon, mineral, and salt elements was found in the tap water sample.

From the SEM images, residual density was estimated on 8 mm × 8 mm aluminum substrates for each sample. The total number of residuals found in the HPLC sample was much sparser than that of tap water samples. In fact, the estimated residual particle density for particle diameters in the range of 0.3–10 μm was substantially lower for the HPLC sample (47 particles/64 mm^2^) as compared to tap water samples (1.3 × 10^6^ particles/64 mm^2^ and 6.9 × 10^5^ particles/64 mm^2^ for filtered and nonfiltered samples, respectively). The aluminum substrate itself had only 10 particles/64 mm^2^ as background contaminants, which are mostly composed of Al, C, N, and O.

**Figure 3:**
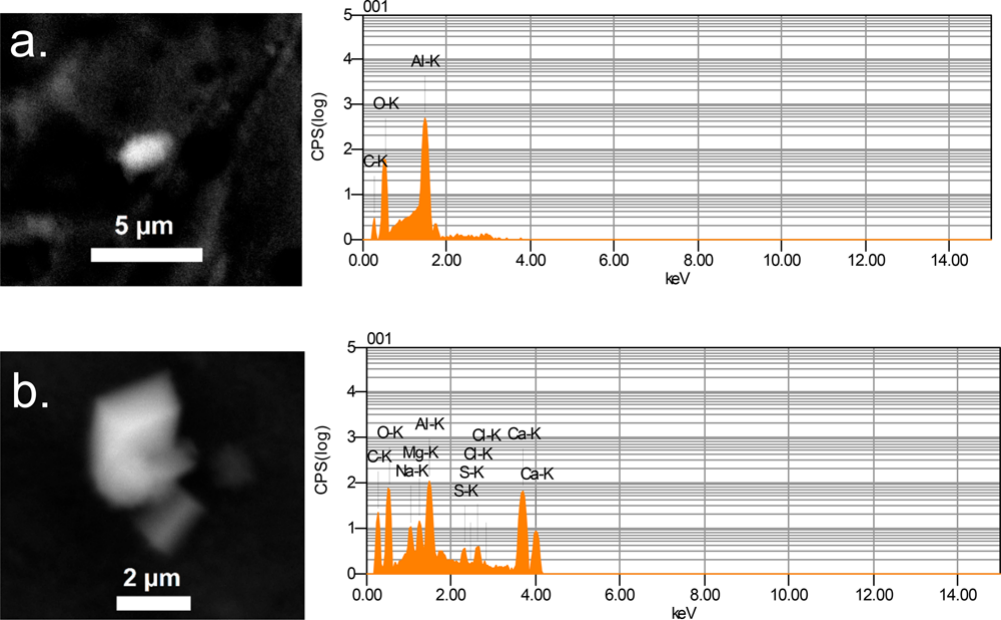
Representative electron microscopy images and EDX spectra of (a) the organic dominant residual particle in the HPLC sample and (b) a typical mineral including particle found in the filtered tap water sample.

The EDX analysis assessed the Atomic % of organic (C, N, O), salt-rich (Na, Mg, K, P), mineral-rich (Si, Ca), and others. Specifically, we investigated 47, 100, and 100 residuals in the HPLC, filtered tap, and original tap water samples, respectively. Residuals from the latter two samples were randomly selected from an 8 mm × 8 mm cross-section. Representative SEM images and identified elements are shown in Figure 3. A few oxidized organic-dominant particle residuals were found in the HPLC sample, whereas a substantial amount of mineral plus salt-rich particles was identified in the tap water residuals. Table 5 summarizes elements (excluding aluminum) and atomic % identified in water residuals as well as on a background aluminum substrate. As seen, similar inorganic elements were found in tap water residuals. On the other hand, residual particles found in HPLC water mainly contain C, N, and O with a trace amount of N, Mg, Si, and Fe. No substantial amounts of other minerals and salt elements were found in the examined HPLC water. A similar result (but with lower O) was found for the substrate background. The observed similarity implies that residuals found in the HPLC water sample might have been in part from the substrate itself, but highly oxygenated materials may have derived from HPLC water. It is worth noting that the EDX analysis on a small number of particles from a single sample cannot provide any statistically valid conclusions or size distribution data (see the assessment discussion in SI Sect. S2, SEM-EDX Module).

**Table 5:** Summary of Elements and Atomic % ± Standard Error Identified in Water Residuals^a^

	Atomic %			
Element	HPLC water (*n* = 47)	Filtered tap water (*n* = 100)	Tap water (*n* = 100)	Substrate Background (*n* = 10)
C	26.03 ± 3.32	17.61 ± 0.36	17.64 ± 0.37	53.69 ± 4.14
N	1.29 ± 0.76	1.24 ± 0.06	0.93 ± 0.05	9.08 ± 0.62
O	68.22 ± 3.65	62.63 ± 1.00	61.87 ± 0.73	24.26 ± 4.76
Na	0.05 ± 0.02	8.98 ± 0.59	9.80 ± 0.50	0.05 ± 0.04
Mg	0.55 ± 0.24	4.63 ± 0.25	6.25 ± 0.68	3.02 ± 0.36
Si	3.02 ± 1.39	0.91 ± 0.04	1.20 ± 0.04	6.11 ± 2.99
P	0.01 ± <0.01	0.03 ± <0.01	0.02 ± < 0.01	0.34 ± 0.13
S	0.01 ± 0.01	0.65 ± 0.04	0.66 ± 0.09	0.02 ± 0.01
Cl	<0.01 ± <0.01	2.41 ± 0.32	0.87 ± 0.17	0.03 ± 0.01
K	0.08 ± 0.05	0.16 ± 0.01	0.13 ± <0.01	0.03 ± 0.01
Ca	0.01 ± <0.01	0.31 ± 0.09	0.19 ± 0.05	0.04 ± 0.02
Mn	0.01 ± <0.01	0.02 ± <0.01	0.02 ± <0.01	0.05 ± 0.02
Fe	0.64 ± 0.42	0.37 ± 0.10	0.32 ± 0.04	2.56 ± 0.91
Zn	0.09 ± 0.02	0.19 ± 0.01	0.22 ± 0.01	0.74 ± 0.09

a Aluminum was excluded to eliminate the background signal from an Al substrate. The numbers in the parentheses represent the sub-total of the number of particles investigated from the sample.

The observed predominance of salt and mineral particles in tap water types was expected as the local municipal water is typically enriched in those elements.^19^ Although only 47 precipitates were identified for the HPLC water sample, they were mainly organic. This observation is interesting because the lack of salt and mineral precipitates in the HPLC water sample indicates that these elements might be mainly responsible for the observed gap in freezing spectra of filtered tap water and HPLC water sample shown in Figure 1 (at least in part). Nevertheless, what creates a gap between the HPLC water spectrum and the homogeneous freezing curve (Figure 1) remains unknown. SI Sect. S1 Module 3 provides additional insights into the impurities (i.e., methyl/alkyl organic compounds) in the same water samples through a gas chromatography system coupled with a mass selective detector (GC-MS) and nuclear magnetic resonance spectroscopy (NMR) instrument. Subsections in SI Sect. S1 Module 3 describe each analytical result. Based on the previous literature, eliminating them via chemical treatment may be key to realizing artifact-free freezing experiments (see SI Sect. S6).

### 3.4 Class Assessment

#### 3.4.1 Students’ vs Model Results

In this section, measurable and tangible performance of the students in a classroom setting for each module is discussed in comparison to the model results of the instructors.

For Module 1, the students’ outcomes, summarized in Table 6, in general, agree with the model results from the instructor and interns (Table 3). The students’ results, which exceed the desired standard (See SI Sect. S2 1.1.2), reflect adequate guidance, instruction, and practical support during the module lesson. An evaluation of the missed exercise questions revealed that the majority of the errors were in the mathematical calculations of surface tension and miscalibration of the pH and conductivity sensor. A supplemental math workshop as well as more hands-on experimental support by instructors (including a teaching assistant) are envisioned for the future class to improve the students’ math skills.

**Table 6:** Student Data of Bulk Water Properties: Average ± Standard Deviation of Three Replicated Measurements (Subtotal of the Number of Particles Investigated from the Sample)

	pH	Conductivity (μS cm^–1^)	Surface tension (dyn cm^–1^)	*T* (°C)
Unfiltered tap water (*N* = 6)	7.8 ± 0.4	705.2 ± 77.0	72.8 ± 1.0	12.2 ± 0.9
filtered tap water (*N* = 6)	7.7 ± 0.2	899.2 ± 6.9	72.0 ± 0.9	14.1 ± 0.5
HPLC water (*N* = 18)	5.9 ± 0.2	21.5 ± 3.0	74.0 ± 2.3	22.62 ± 0.1
DI water (*N* = 6)	6.5 ± 0.3	962.6 ± 86.3	73.4 ± 1.5	15.5 ± 0.3

**Figure 4:**
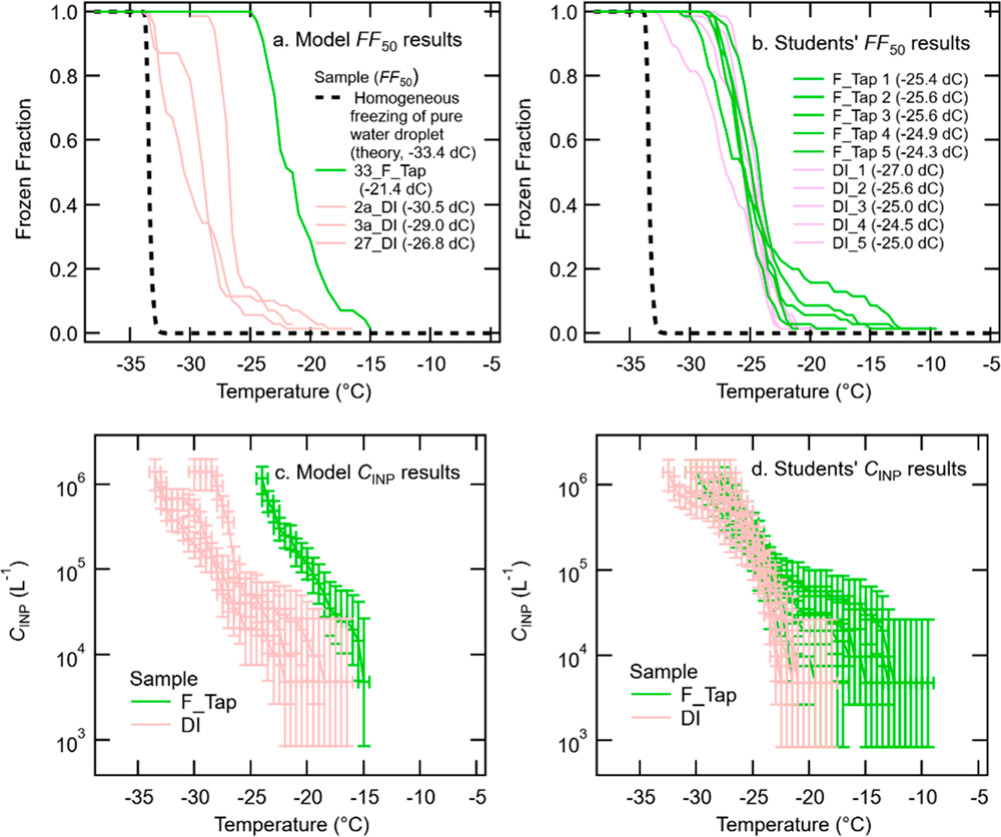
Model results vs students’ results of *FF*(*T*) (a, b) and *C*_INP_(*T*) ± CI95%(*T*) (c, d) for filtered tap water (F_Tap) and DI water samples. Note: the *C*_INP_(*T*) calculation is described in SI Sect. S5 Eqn. S2.

Figure 4 shows the summary of the students’ results in comparison to the model results for Module 2. In general, the students’ outcomes agree with the model results but some deviations are identified. As can be inferred by the figure, the DI water samples showed the suppression of freezing temperatures and activities as compared to the filtered tap water samples. This result was expected for the reason discussed in Sect. 3.2. The *FF*_50_ value of the model DI water results (−28.8 ± 1.9 °C, average ± standard deviation) was slightly lower than that of the students’ results (−25.5 ± 1.0 °C). On the other hand, the *FF*_50_ value of the model filtered tap water results (−21.4 °C) was higher than that of the students’ results (−25.2 ± 0.6 °C). The observed offset may be stemming from different stock tap water samples used for filtration and deionization for the students and/or other deviations during the sample preparation. Nonetheless, the students were successfully able to visually capture the “hump” feature of active INPs in both *FF*(*T* > −20 °C) and INP concentration in suspension, *C*_INP_(*T* > −20 °C), plots for the filtered tap water while they observed the step function-like activation in the DI water samples. The INPs in filtered tap water are presumably pre-existing contaminants in tap water (refer to the introduction section of the elemental composition analysis of water residual particles by SEM-EDX module). The students also successfully estimated CI95%. They understood that it is important to represent the experimental uncertainty of the immersion freezing experiment with binomial CI95% errors rather than a constant systematic error as the former can capture the temperature-dependent uncertainties. As fewer particles freeze at high temperatures in general, the associated *C*_INP_(*T*) error at those temperatures is typically larger than at lower temperatures (panels c and d of Figure 4).

For Module 3, the students compared their standard error in their atomic % results to the instructor’s results. A summary of comparison data is shown in Table 7. An evaluation of the two data sets did not indicate a substantial deviation since the statistical uncertainty (i.e., standard error) from the students’ data are comparable to that of the instructor. In general, the students were able to identify high inclusion of salts in their filtered tap water samples (Na, Mg, S, Cl, and K) and high carbon content in the HPLC water samples, which is also consistent with the instructor’s model data. After the evaluation, instructors recap the importance of examining large particle sizes (nominally > 10,000 particles) to statistically represent particle properties and ambient particle data to the students.^25,26^ Nevertheless, the representativeness of single particle analysis is beyond the scope of this educational activity.

**Table 7:** Summary Comparison of Elements and Atomic % ± Standard Error Identified in Water Residuals for Model Results (Table 5) vs the Student Results^a^

	Atomic %			
	Table 5	Table 5	Student	Student
Element	HPLC water (*n* = 47)	Filtered tap water (*n* = 100)	HPLC water (*n* = 31)	Filtered tap water (*n* = 100)
C	26.03 ± 3.32	17.61 ± 0.36	28.29 ± 1.54	7.90 ± 0.34
N	1.29 ± 0.76	1.24 ± 0.06	<0.01 ± <0.01	<0.01 ± <0.01
O	68.22 ± 3.65	62.63 ± 1.00	65.15 ± 1.22	72.44 ± 1.00
Na	0.05 ± 0.02	8.98 ± 0.59	0.08 ± 0.01	8.32 ± 0.64
Mg	0.55 ± 0.24	4.63 ± 0.25	1.65 ± 0.35	6.46 ± 0.18
Si	3.02 ± 1.39	0.91 ± 0.04	1.14 ± 0.45	1.11 ± 0.03
P	0.01 ± <0.01	0.03 ± <0.01	0.03 ± 0.01	0.03 ± <0.01
S	0.01 ± 0.01	0.65 ± 0.04	0.20 ± 0.18	0.86 ± 0.03
Cl	<0.01 ± <0.01	2.41 ± 0.32	0.01 ± <0.01	2.16 ± 0.45
K	0.08 ± 0.05	0.16 ± 0.01	0.03 ± 0.01	0.21 ± 0.01
Ca	0.01 ± <0.01	0.31 ± 0.09	3.08 ± 0.28	0.15 ± 0.01
Mn	0.01 ± <0.01	0.02 ± <0.01	0.01 ± <0.01	0.03 ± <0.01
Fe	0.64 ± 0.42	0.37 ± 0.10	0.17 ± 0.02	0.30 ± 0.02
Zn	0.09 ± 0.02	0.19 ± 0.01	0.17 ± 0.01	0.04 ± 0.01

a Aluminum was excluded to eliminate the background signal from an Al substrate. The numbers in the parentheses represent the subtotal of the number of particles investigated from the sample.

#### 3.4.2 Classroom Survey

Based on the formal program assessment of learning outcomes and direct (yet deidentified) student feedback, we overall achieved our goal to (1) improve their problem-solving skills by combining multidisciplinary science and math skills and (2) be able to discuss data and results with variability and uncertainty. The survey responses of the students are generally positive and provide room for improvement in the future.

The student-participating module LOs were assessed for each module using competency and exercise questions (Table 1), and the assessment results are summarized in Table 8. Detailed assessment description is available in SI Sect. S2. Briefly, the students’ results in the numerical characterization of bulk water properties (pH, conductivity surface tension, and temperature) (LO1), as well as the data deviation for different samples (LO2), were assessed by tracking percentages of students that correctly answered a subset of exercise questions (listed in SI Sect. S2 1.1). The criteria (i.e., 90% and 80% benchmark for Lo1 and LO2, respectively) were comfortably met for given exercise questions (*N* = 10). For Module 2, the numerical skill to compute *FF*(*T*) and *C*_INP_(*T*) (LO1), as well as statistical defensibility (LO2), of students was ultimately assessed based on the pass/fail threshold. While students met the target, considerable effort was required to advise even a small number of students (*N* = 5) for math tutoring and the exercise questions. Finally, using the SEM-EDX data from Module 3, the numerical conversion of elemental weight % to atomic % (LO1) and statistical analysis of identified elements, as well as formulation of tabular data (LO2), trained students (*N* = 5) with exceeded benchmark target (i.e., the score of >80%).

**Table 8:** Summary of Learning Outcomes for Each Module (De-Identified Individual Basis)

Module 1^a^			Module 2^a^			Module 3^a^		
	LO 1 (*N* = 17^b^)	LO 2 (*N* = 18)		LO 1 (*N* = 5)	LO 2 (*N* = 5)		LO 1 (*N* = 5)	LO 2 (*N* = 5)
Student 1	100.0	87.0	Student 1	76.0	72.0	Student 1	83.3	75.0
Student 2	97.5	100.0	Student 2	79.0	77.0	Student 2	83.3	90.0
Student 3	95.0	81.6	Student 3	89.0	86.0	Student 3	100.0	94.0
Student 4	85.0	70.6	Student 4	87.0	91.0	Student 4	100.0	100.0
Student 5	95.0	65.0	Student 5	98.0	97.5	Student 5	100.0	96.5
Student 6	100.0	93.1	Avg. ± Std. Error	85.8 ± 3.9	84.7 ± 4.6	Avg. ± Std. Error	93.3 ± 4.1	91.1 ± 4.3
Student 7	100.0	99.8						
Student 8	90.0	83.9						
Student 9	97.5	84.8						
Student 10	100.0	54.1						
Student 11	58.8	79.5						
Student 12	90.0	79.5						
Student 13	97.5	90.0						
Student 14	85.0	80.0						
Student 15	100.0	90.6						
Student 16	100.0	85.7						
Student 17	100.0	81.9						
Student 18	N/A	85.0						
Avg. ± Std. Error	93.6 ± 2.5	82.9 ± 2.7						

a Competency & exercise questions used for the assessment are listed in Table 1.

b Environmental Science majors only as stated in SI Sect. 2 1.1.1.

Next, we provide the information regarding the postactivity survey questions and typical feedback responses for each module. For Module 1, the students raised concerns about uncontrolled/inconsistent experimental variables, such as the temperature of water samples exposed to the lab air and sample storage period, as well as conditions of the apparatus, which can impact the consistency and reproducibility of the desired results. Likewise, the students addressed some issues regarding inconsistency in experimental variables in Module 2. For instance, inhomogeneous size of water droplets, as well as deviation in preparation time and associated artifact (e.g., particle settling in stack suspension, exposure to air increasing the chance of including contaminants in early prepared droplets, Vaseline clogging the tips of the pipet). Other student concerns include the labor-intensive nature of the WT-CRAFT operation, subjective interpretation of droplet freezing temperature based on visual inspection (though ImageJ inspection can help), stability of the thermostat, and presence of static. Lastly, for Module 3, the students experienced issues in obtaining similar electron counts (related to the electron beam filament condition), finding submicron particles (sample pending), necessity of manually adjusting image quality for each particle and its time-consuming nature, and potential contamination during the sample preparation.

All issues can be troubleshot by the instructors. Having TAs would help faculty who wish to adopt these modules. More detailed notes from the assessment are available in SI Sect. S2 (Subsections 2.1.2, 2.2.2, and 2.3.2). All of the experimental data to generate figures presented in this study are saved in the SI Sect. S7 folder. This additional information and resources might be useful for potential adopters.

#### 3.4.3 General Remarks

The developed modules in this work are useful to advance a college curriculum in environmental science as they include (1) fundamental concepts of environmental problem solving, (2) numerical approximations to exact mathematical solutions, and (3) concepts of uncertainty and their application to earth and environmental sciences. These modules are especially meaningful to integrate research and education and comprehensively enhance students’ understanding of the importance of scientific measurements and data. While the one-to-one training is effective, increasing the number of apparatus and teaching assistants to conduct the group training in the classroom setting might improve teaching efficiency in the future.

As demonstrated during the module development, bulk water sample characterization, immersion freezing analysis, and artifact composition assessment provide meaningful curricular activities, which could be adapted to college STEM courses (e.g., fundamental environmental science, numerical methodology, and advanced graduate-level experimental chemistry lab courses). The developed modules can also provide students with a meaningful experience to understand advanced instrumentation and bulk sample analytical techniques available even at a typical primary undergraduate-teaching institution.

## 4 Conclusion

To integrate research and education in atmospheric ice nucleation, lab experiment-based modules and problems were developed. These modules were implemented in the classroom setting, and a total of 46 students (18 interns and 28 in-class students) were trained. The developed curricular products provided immediate hands-on opportunities for the student to apply aerosol measurement technologies, exchange ideas with their mentor(s), and disseminate scientific findings through renowned science conferences.

Students determined proper sample preparation and offline measurement procedures for atmospheric ice nucleation research. First, by comparing the freezing properties of DI water and HPLC water, students were able to visualize how pre-existing INPs in DI water could impact the freezing behavior of water and how reproducible the HPLC water *FF* result is when compared to the DI water one, which shows a wide range of uncertainty (i.e., standard error). Students also gained experience in characterizing freezing efficiencies with INP suspensions of known compositions (i.e., Illite NX, Snomax, and MCC). This exercise is useful to train a diverse group of students with different academic backgrounds (i.e., biology, chemistry, engineering, environmental science, and physics). Finally, a comprehensive curriculum of freezing assay calibration and droplet freezing experiments with different types of water samples (including but not limited to HPLC, tap, and DI water) using the WT-CRAFT system and complementary analyses are ideal experiments for all levels of STEM students.

Through the implemented activities, students also successfully characterized the physicochemical and freezing properties of their water samples. We examined potential artifacts in water freezing and identified some compounds that are relevant to the freezing of each water sample. When compared to ultrapure water, the tap water exhibited higher ion conductivity and pH, which is indicative of the inclusion of impurities (e.g., mineral and salt ions)_._ In fact, SEM-EDX analysis allowed students to identify the nonvolatile minerals and salts present in the tap water samples. These compounds identified may be serving as ice nucleation active impurities in the tap water samples, causing heterogeneous freezing. Complementary analysis of organic INP beyond the detection capability of the techniques used in this study may further reveal the identity of INPs in pure water. More specifically, assessing the same samples by means of other analytical techniques with higher sensitivity to water impurities (e.g., HPLC, Raman microspectroscopy) compared to the used instruments in this study may further reveal the chemical identity of INP and understand what causes heterogeneous freezing in water samples.

In the end, the outcomes of the participation by 28 in-class students in modules were assessed. Based on their input, we further improved shareable read-ahead exploration material to introduce future students to the scientific concepts and ensure that they have an understanding of the science that they convey via the prefabricated modules. In 2022, the developed modules directly impacted 28 students (18 undergraduates and 10 graduates) at West Texas A&M University. Our lesson plans and materials can be implemented at other educational organizations to teach their students about atmospheric ice nucleation in their curriculums.

## Supporting Information Available

Original data used to create the figures, modules 1–3, module assessment document, additional module instructions and assessment details, suface tension estimation, water freezing test data, freezing assay calibration procedure, and advancing freezing assay (ZIP)

## Notes

The video data used for the study can be found in the PANGAEA Data Archiving and Publication (https://doi.pangaea.de/10.1594/PANGAEA.952536)
