# Supplementary material for: Integrated Science Teaching in Atmospheric Ice Nucleation Research: Immersion Freezing Experiments
Source: J Chem Educ. 2023 Mar 8;100(4):1511–22. doi: 10.1021/acs.jchemed.2c01060 (PMC10100551; doi:10.1021/acs.jchemed.2c01060)
Supplement: Supplementary file 1 — ed2c01060_si_001.zip [file ed2c01060_si_001.zip › SI_Files/SI_Sect_S1_Module_1_BULK_PROPERTIES.docx]

**Supporting Information:**

**Integrated Science Teaching in Atmospheric Ice Nucleation Research:**

**Immersion Freezing Experiments**

Elise K. Wilbourn^1,♦^, Sarah Alrimaly^1,♦^, Holly Williams^1^, Jacob Hurst^2^, Gregory P. McGovern^2^,

Todd A. Anderson^3^, and Naruki Hiranuma^1,^*

^1^Dept. of Life, Earth, and Environmental Sciences, West Texas A&M University, Canyon, TX, 79016

^2^ Dept. of Chemistry and Physics, West Texas A&M University, Canyon, TX, 79016

^3^ Dept. of Environmental Toxicology, Texas Tech University, Lubbock, TX, 79416

^♦^These authors equally lead and contributed to this work

*Corresponding author ([nhiranuma@wtamu.edu](mailto:nhiranuma@wtamu.edu))

# **Title: Module 1 - Characterization of bulk water properties**

Estimated Completion Times: 225 min

Introduction 45 min

Experimentation 150 min

Assessment 30 min

Prerequisites: Chemistry I.

Target course level: all undergraduate-level in Environmental Science/Chemistry.

Overall Objectives:

The bulk properties of water are useful as a general measure of water quality and other environmental parameters. Any water sample has a relatively constant range of bulk properties that, once established, can be used as a baseline for comparison with measurements of other aqueous samples. You will learn about the characterization of pH, electrical conductivity, and surface tension of water and their atmospheric relevancy.

## **INTRODUCTION**

### Introduction Summary

This part contains targeted background content to prepare you for performing the bulk water characterization in this lesson.

### Learning Objectives

- Define the pH of a solution.
- Describe the conductivity of water.
- Explain the surface tension of water.

### Test Your Knowledge

1. Acid precipitation is rain and snow with a pH below [ ].

- 5.6
- 7
- 8.6
- 10

1. pH stands for potential [ ].

- hydrogen
- hydrocarbon
- hexafluoride

1. What is the physical unit of electrical conductivity measurement of an aqueous solution?

- S/cm
- m/µs
- N/m

1. What is the physical unit of surface tension measurement of an aqueous solution?

- S/cm
- m/µs
- N/m

### Answer Key: 1. 5.6 2. hydrogen 3. S/cm 4. N/m

### Subsection 1: pH

The abbreviation pH stands for potential hydrogen. pH is a measure of the acidity and basicity of a solution. The pH value can be represented by the concentration of hydrogen ions, [H^+^], while the more accurate representation is the concentration of hydronium ions, [H_3_O^+^]. The species H^+^ is known as a proton. The proton remains when a hydrogen atom loses its electron, and H_3_O^+^ forms in solution with a combination of H^+^ and H_2_O. For instance, an acid produces H_3_O^+^ in the solution by donating a proton (H^+^) to H_2_O.

The self-ionization of water molecules, the so-called autoprotolysis of water, generates H_3_O^+^ and hydroxide ion, OH^-^.

$H_{2}O+H_{2}O\underset{\Leftrightarrow}{K_{w}}H_{3}O^{+}+{OH}^{-}$ [1]

While H_3_O^+^ ions are more representative in an aqueous solution than H^+^, it is often interchangeably abbreviated as H^+^. In short, autoprotolysis of water can be expressed as an abbreviation of [1] by considering a dissociation of water into hydrogen ions and hydroxide ions.

$H_{2}O\underset{\Leftrightarrow}{K_{w}}H^{+}+{OH}^{-}$ [2]

Autoprotolysis is constantly occurring in water, and the autoprotolysis constant for pure water, *K*_w_, can be expressed as:

$K_{w}=\left[ H^{+} \right] \left[ {OH}^{-} \right]=1.0\times{10}^{-14} at-25 ^{\circ}C$ [3]


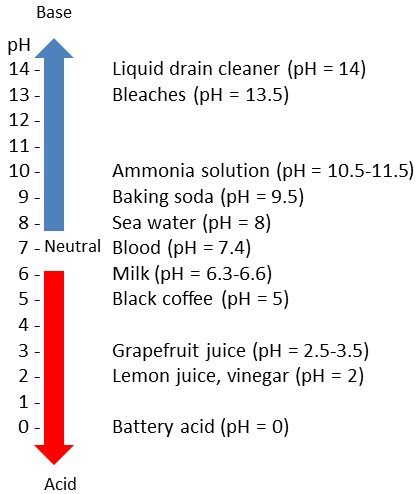
Thereby, an increase in H^+^ concentration requires a decrease in OH^-^ concentration, and vice versa. With this constant, you can find the concentration of either species if the concentration of the other is known. To simplify the writing of H^+^, you can generally define pH in water as the negative base 10 logarithm of the hydrogen ion concentration of a solution:

$pH= -log \left[ H^{+} \right]$ [4]

Thus, to calculate the pH of an aqueous solution, you only need to know the concentration of the hydrogen ion concentration in moles per liter (M, molarity).

**Figure 1** shows the pH of some solutions you can commonly find in your everyday life. A neutral solution has a pH of 7. Acidic and basic solutions have lower and higher pH values, respectively. Since the pH value is scaled logarithmically (i.e., Eqn. 4), an increase or decrease of one pH unit means a factor of ten difference in [H^+^] and [OH^-^].

According to the U.S. EPA and USGS, unpolluted precipitation water has a slightly acidic pH of about 5.6. This non-neutral pH is mainly due to carbon dioxide (CO_2_) dissolved in hydrometeors and resulting in weak carbonic acid. In general, acid rain (or, more precisely, acid precipitation) has a much lower pH (~4.3) due to the deposition of acidic components (e.g., sulfuric and nitric acids in both aerosol and gas phases) in hydrometers. As you may be aware, acid precipitation can deteriorate the conditions of natural environments (lakes and forests), biomes, ecosystems, buildings, and historical monuments.

**Figure 1**. pH scale for various natural and manmade substances. Image adapted from OpenStax College (https://commons.wikimedia.org/wiki/File:216_pH_Scale-01.jpg). License link: https://creativecommons.org/licenses/by/3.0/deed.en

### Subsection 2: pH and Atmospheric Ice Nucleation

A small amount of atmospheric aerosol particles can act as ice-nucleating particles (INPs) in mixed-phase clouds and modulate clouds, precipitation, and Earth’s radiative energy balance. Despite the importance of INPs and ongoing efforts, the current understanding of the physicochemical properties of INPs is still scarce. The bulk properties of the water samples that you will examine in this module may have inherent and non-negligible influences on ice nucleation.

The particle surface pH can impact atmospheric ice nucleation. It is known that the structure of interfacial water near the surfaces is crucial to heterogeneous ice nucleation (e.g., Li et al., 2012). More specifically, the structure of water molecules in the interfacial region near the charged surface can induce structural match/mismatch to ice and therefore impact ice nucleation. A recent experimental study shows that ice nucleation active particles can possess a high isoelectric point (i.e., the pH at which the particle surface carries zero charge potential) and high charge density (Hiranuma et al., 2014). The high isoelectric point implies that the surface of particles is basic, possibly due to the presence of a higher fraction of the basic groups and/or due to a change in the coordinative environment of the functional groups and their enhanced chemical activity at surface kinks and edges (Schindler and Stumm, 1987).

While the surface charge and the associated pH of bulk water might not exclusively explain the ice nucleation activity of a suspension, it is important to measure them as a baseline for comparison with measurements of other aqueous samples. Otherwise, experiments at a consistent neutral pH may be required.

### Subsection 3: Conductivity

Electrical conductivity is the measure of the concentration of ions that transmit an electrical current present in a solution. The measurement unit for electrical conductivity is called Siemens (S) (e.g. Micro Siemens per cm, μS/cm). Solutions containing electrolytes such as acetic acid are conductive, and the relative conductivity of acids can be directly related to their different degrees of dissociation in an aqueous solution. Hence, you can expect that 10 mM acetic acid has a tenfold higher conductivity than 1 mM acetic acid. While not all electrolytes necessarily act as INPs, a previous study by Whale et al (2018) shows that ≤150 mM concentrations of ammonium salts and alkali halides can have a substantial impact to alter the ice-nucleating efficiency of some nucleators, such as microcline feldspar and quartz.

Different types of substances have various conductivities when dissolved in water. For example, the presence of salts and other inorganic chemicals that conduct electrical current in a solution can result in an increase in conductivity. On the other hand, some organic compounds such as oil and sucrose are not good electrical conductors and therefore have a low (or negligible) conductivity when mixed with water. Furthermore, conductivity is temperature-dependent. Hence, the warmer the water, the higher the conductivity.

Water samples with elevated conductivity may also contain water-soluble and/or insoluble particles. Typically, atmospheric hydrometers (e.g., rain and snow) have a conductivity of <100 μS/cm. Your drinking water has a conductivity value of several hundred μS/cm. Typical seawater contains many ions, and the resulting electrical conductivity is much higher (~50 mS/cm) than other water sources.

###
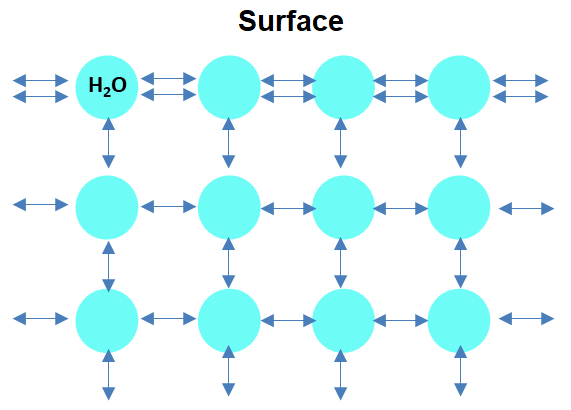
Subsection 4: Surface Tension

Surface tension represents the property of the surface of a liquid that allows it to resist an external force, due to the cohesive nature of its molecules. For instance, water molecules attract each other, leading to the formation of surface tension. At the surface of bulk water (or air-water interface), the bond between water molecules is stronger because fewer water molecules exist at the outmost layer than in the sublayers of water molecules (see **Fig. 2**), resulting in the surface tension of 72.53 dyn/cm (equivalent to mN/m) at 20 °C (Gianino, 2006). The given surface tension of water explains for instance the reason why you see rain as a spherical drop. High surface force shapes the water drop to be spherical with as little surface area as possible (spherical surface area < cubic surface area).

**Figure 2**. Surface tension in water. Due to surface tension, molecules at the surface form stronger bonds. Image resides in the Public Domain but adapted from USGS (2019).

Surface tension is a measure of force per unit length, and you will learn how to measure it using a surface tensiometer in this module (discussed more in detail in **Exercise 2 Part 2**). While surface tension is known to be time-dependent until the equilibrium status of surface-active molecules (surfactants) and water is achieved (e.g., Lin et al., 2019), you will only learn about “static” surface tension at equilibrium in this module.

In the atmosphere, the inclusion of bulk/surface partitioning of surfactants can suppress the surface tension of cloud droplets and impact cloud condensation processes. In short, the depression of droplet surface tension by surfactants is expected to increase cloud condensation efficiency via the Kelvin effect; the depletion of solute from the bulk will also decrease cloud condensation activity via the Raoult effect (e.g., Lin et al., 2019). While the specific relationship between surface tension and surface composition is typically unknown, it is key to measure surface tension and refer to it as a parameter of cloud formation properties.

### Subsection 5: Calibration and Generating a Standard Curve

A standard curve, also known as a calibration curve, can visualize the relationship between measured and standardized values. Using a standard curve, you can adjust the measured values by comparing them to a set of standard values of known samples/properties. In **Exercise 1**, you will generate a simple linear standard curve and equation with the form of *y* = *m x*_i_ + *b*, where *x*_i_ is your measured value, *y* is the adjusted (or true) value, *m* is the slope (∆y/∆x), and *b* is the y-intercept.

The least-squares method defines the process of fitting a mathematical function to a set of measured points by minimizing the sum of the squares of the distances from the points to the curve (e.g., Harris, 2000). Here, you can define the distance as a vertical deviation (*d*_i_) for each set of your measured value, *x*_i_, and pre-adjusted standard value, *y*_i_, as:

$d_{i}=y_{i}-y=y_{i}-\left( mx_{i}+b \right)$ [5]

Hence, the square of the distance can be expressed as:

$\left( d_{i} \right)^{2}=\left( y_{i}-y \right)^{2} =\left( y_{i}-mx_{i}-b \right)^{2}$ [6]

**Figure 3** illustrates a snapshot example of the standard curve and associated parameters for the given four pairs of the dataset of *x*_i_ (= 1, 3, 4, and 6) and *y*_i_ (= 2, 3, 4, and 5). For the given *d*_i_^2^, you can compute the m and b values as:

$m=\frac{n\sum\left( x_{i}y_{i} \right)-\sum x_{i}\sum y_{i}}{D}$ $\mathrm{where}D=n\sum\left( x_{i} \right)^{2}-\left( \sum x_{i} \right)^{2}$ [7]

$b=\frac{\sum\left( x_{i} \right)^{2}\sum y_{i}-\sum\left( x_{i}y_{i} \right)\sum x_{i}}{D}$ [8]

**Table 1** guides you in the calculation of $\sum x_{i}$, $\sum y_{i}$, $\sum\left( x_{i}y_{i} \right)$, and $\sum\left( x_{i} \right)^{2}$ for the given data points of *x*_i_ and *y*_i_. For the given dataset, thereby, you can compute *D* (52 = 4 x 62 - 14^2^), *m* (0.62 = (4 x 57 – 14 x 14) / 52), and *b* (1.35 = (62 x 14 – 57 x 14) / 52). Therefore, the fit equation is *y* = 0.62 *x*_i_ + 1.35.


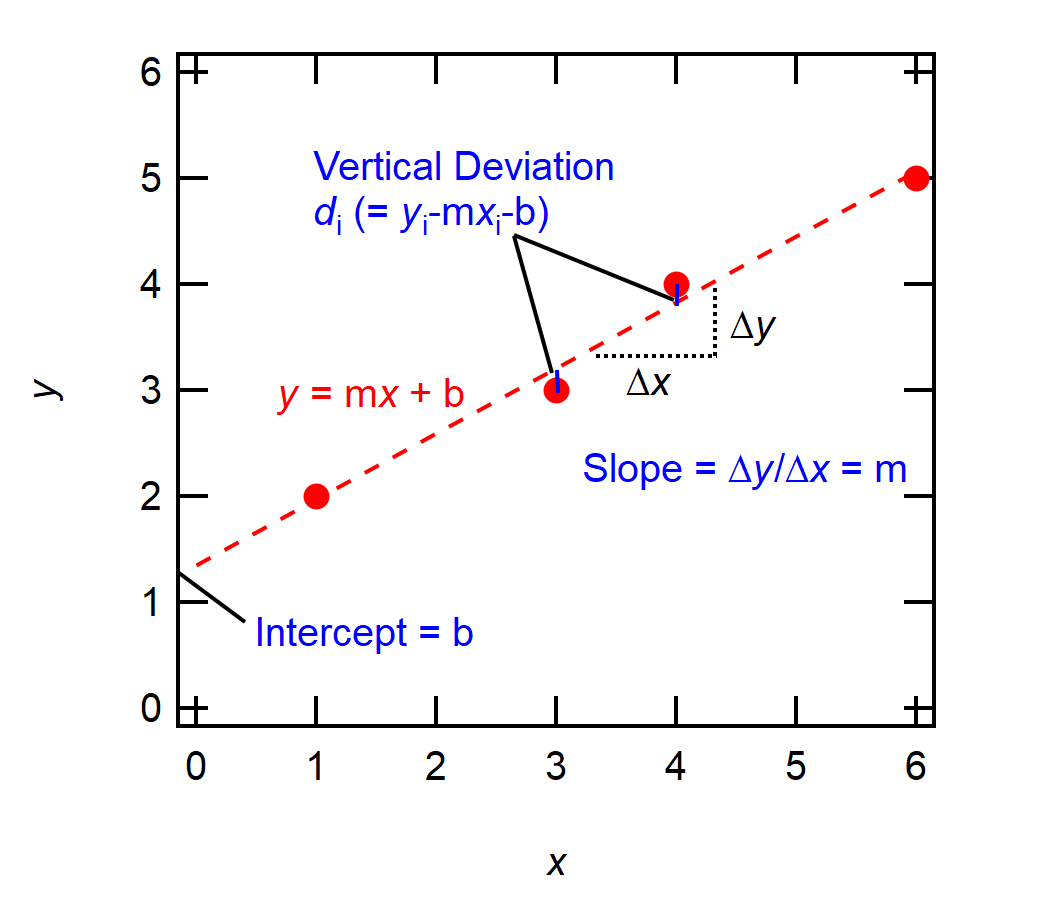


**Figure 3.** Example of the standard curve and vertical deviation.

**Table 1.** The calculation for some variables in the least-squares analysis.

|  | *x_i_* | *y_i_* | *x_i_y_i_* | *x_i_^2^* | *d_i_^2^ (=(y_i_-mx_i_-b)^2^)* |
| --- | --- | --- | --- | --- | --- |
|  | 1 | 2 | 2 | 1 | 0.0015 |
|  | 3 | 3 | 9 | 9 | 0.0370 |
|  | 4 | 4 | 16 | 16 | 0.0370 |
|  | 6 | 5 | 30 | 36 | 0.0015 |
| Σ | **14** | **14** | **57** | **62** | **0.0770** |

## **EXPERIMENTATION**

### Experimentation Summary

This section will guide you through competency-building exercises. You will be challenged with applying the knowledge you gained in the Exploration to complete the activities successfully.

### Learning Objectives

- Analyze two water samples for pH, conductivity, and surface tension.
- Learn the instrument calibration by developing a standard curve.

### Hypothesis Formulation

1. Do you expect there to be a relationship between pH and electrical conductivity?
2. Would you expect the surface tension of tap water to be lower than that of ultrapure water (based on the theory)?

### Materials

Read through the procedures listed in the exercises on the next pages before beginning. Then, gather all of the materials listed below and begin Exercise 1.

| **Qty.** | **Item** | **Manufacturer, Model, Estimate ~$1,400** |
| --- | --- | --- |
| 1 | pH/conductivity probe* | Oakton, Waterproof pH/Con 10 meter, $450 |
| 1 | Conductivity Standard (23, 100, 447, and 2764 µS) | Oakton, ZM-00653-23, -35, -47, -20, $100 |
| 1 | pH Reference Standard Buffers (pH 4, 7, and 10) | VWR, BDH5100-PK, $260 |
| 1 | Surface Tensiometer** | DWK Life Science, Model 14818, $305 |
| 1 | Sterile Syringe Filter | VWR, 28145-477, $75 |
| 1 | 50 mL Luer-Lok Syringe | VWR, 309653, $95 for 40 |
| 1 | Laboratory Bottles, High-Density Polyethylene | VWR, 414004-159, $85 for 6 |

The following materials might be available at your laboratory or the university physical plant.

| **Qty.** | **Item** |
| --- | --- |
| 4 | 250 mL borosilicate beaker |
| 1 | permanent marker |
| 1 | 250 mL graduated cylinder |
| 1 | Tap water (200 mL) |
| 1 | Distilled water |
| 1 | Ultrapure water (200 mL 18.2 MΩ•cm MilliQ water or HPLC grade water, Sigma Aldrich, 270733) |
| 4 | Pair of gloves |
| 1 | Isopropyl alcohol |
| 1 | Kimwipes |

*pH/conductivity probe:

The Oakton Waterproof pH/Con 10 meter measures electrical conductivity by conductance between two electrodes using amperometric methods. When submerged in an aqueous sample, the electrodes pass a current through the sample solution. The relative concentration of ions within the solution will determine the conductivity or the resistivity of the sample. A pH electrode consists of a reference electrode, reference solution, reference junction, and a glass bulb with a hydrated gel layer. Once the electrode is submerged in a sample this creates a current that measures the charge of the reference solution inside the bulb compared to the solution on the outside of the gel layer. The result is a pH measurement.

**Surface Tensiometer:

The employed surface tension analyzer consists of a glass capillary tube (0.5 mm nominal inner diameter) and an outer glass cylinder with tubulation covered by a rubber cap. As surface tension leads to the phenomena of suspension capillary rise or depression, positive (or negative) pressure can be introduced into the tensiometer to estimate the surface tension based on the measurement of capillary depression or rise (See **Exercise 2 Part 2**).

### Safety

- Safety is everyone’s responsibility in the lab. You must always act responsibly and be able to identify potential dangers and take the proper precautions in the event of an accident. In the lab, the most important tools you have to avoid potential issues are your brain and the ability to plan out the future. After previewing Exercise 1, consider what safety precautions you need to conduct a safe experiment and how to put those precautions into action.
- Safety Data Sheets (SDSs) will be provided for all chemicals used in this module. SDSs provide information about physical properties, health risks, fire explosion data, and other important information associated with these chemicals. Before handling or using a chemical, you should refer to the SDS for that chemical.
- It is your responsibility to inform the instructor in writing of any health conditions that may prevent you from safely using a chemical (pregnancy, autoimmune deficiency, etc.). It is also the responsibility of the student to report any spill or problems found while storing or using a chemical. If you are unsure about a chemical, always ask. If you see any unsafe conditions, notify your instructor immediately. If you are unsure about the proper and safe operation of any piece of equipment, ask your instructor for proper instructions. All injuries, spill of materials, and unsafe conditions must be reported to the instructor immediately.
- Any pregnant students, or students planning to become pregnant, should consult their health care provider to determine what, if any, additional precautions are needed based on their individual situation. While the university cannot mandate that the student notify that they are pregnant or are planning to become pregnant, the university strongly recommends that students provide notification so appropriate steps can be taken to ensure the health of both parent and child.
- Eyesight is one of our most important senses in science and should be protected at all times from potential chemical spills and splashes, flying objects and dust, and specific light spectrums. It is important to always wear safety goggles when participating in experiments.
- Food, drinks, and smoking are never allowed in the lab area.
- Always be sure to protect your body from potential harm by keeping your hair tied back, wearing clothing that covers your exposed skin, and by wearing closed-toe shoes.
- Always be sure to protect your work areas from damage caused by experimentation. This means covering your surface in paper towels or plastic when necessary.
- Experiments may also require physical activity so be sure to consider these hazards.
- Be sure to contact your physician first or ask a partner for help in cases that require rigorous physical activity.
- If you need to climb while taking measurements, be sure to use a sturdy stool, chair, or ladder and take the necessary precautions to prevent falling. If you need to climb it is wise to have a partner with you to stabilize what you are climbing. If you are working with moving equipment act cautiously to ensure that the equipment doesn’t lose control and cause injury.
- Almost all chemicals found in a laboratory can be toxic to the human body. To be certain that there is no accidental consumption be sure to never taste, eat, or drink anything in the lab. All labs should be thoroughly cleaned after experimentation to prevent accidental consumption. In the event of chemical ingestion, contact the National Poison Control Center and talk to a physician
- Be certain to wrap all non-chemical experimental items in paper towels or newspapers and throw them into the garbage. Be certain that the container is secured and inaccessible to children and animals.
- If you wear eyeglasses, it is still important to wear goggles over the top of the eyeglasses. This will protect your eyes from chemical spills, shattered glass, and flying objects. Safety goggles must always be worn when conducting experiments.
- Chemical spill protection checklist:
  - Long-sleeved shirts
  - Full-length pants
  - Closed-toe Shoes
  - Protected nitrile gloves
  - Face masks if directed
- Always be sure to pull back or pin down hair to avoid sources of flames, chemicals, or other lab components.
- In the event of an accident always know the location of first aid kits and make sure that they are stocked and easily accessible.
- All laboratories should also have access to eyewash stations that look like water fountains with two upward water faucets that look like they match the distance between eyes. In the event of an accident, the victim's head should be placed close to the fountain. The victim’s eyes should then be held open while the faucets are turned on to spray water into their eyes and clean the chemicals out of their eyes. In non-traditional labs like kitchens, you can also wash out your eyes with a sink faucet or hand-held shower wand. After using an eyewash be sure to contact a physician after washing your eyes.
- A safety shower is also used in all laboratories to put out fires or remove chemicals from the body.

## **Exercise 1 - pH and Conductivity Calibration**

In this exercise, you will examine three water samples (i.e., tap, filtered, and ultrapure water) to characterize their pH, conductivity, and surface tension. **Note: Test all samples as soon as possible after they have been gathered.**

### Part 1: Calibration of your pH and Conductivity Meter (45 min)

1. Put on your gloves.
2. Use the permanent marker to label three beakers pH 4, pH 7, and pH 10.
3. Use the graduated cylinder to pour 100 mL of each pH standard solution into the associated labeled beaker (be sure to rinse the cylinder with deionized water when switching standards).
4. Dip the pH and conductivity sensor tip into the pH 10 beaker as shown in **Fig. 1**.
5. Once the pH number on the sensor monitor stabilizes (you will see “READY” on the screen),
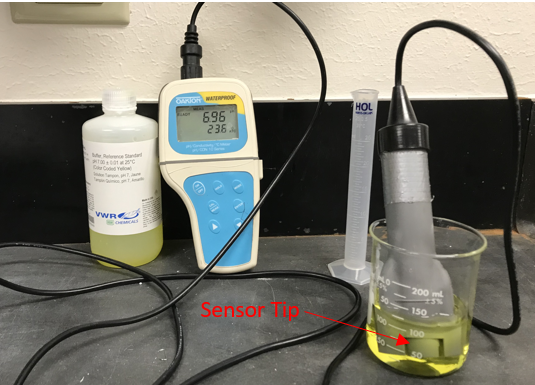
record the measured pH in **Data Table 1** in the pH 10 row.
6. Rinse the sensor tip with distilled water.
7. Repeat Step 4-6 for the pH 7 standard-containing beaker.
8. Repeat Step 4-6 for the pH 4 standard-containing beaker.
9. Use a soft cloth or test tube cleaning brush, Isopropyl alcohol, and distilled water to loosen solids or oils from all laboratory equipment after the completion of an experiment. Thoroughly rinse the items with distilled water and allow them to air dry on clean kimwipes.

**Figure 1**. Experimental setup for the pH calibration

**Data Table 1**. pH calibration table.

| Standard pH (*y*_i_) | Measured pH (*x*_i_) | *Measured Temperature (°C) |
| --- | --- | --- |
| pH 10 |  |  |
| pH 7 |  |  |
| pH 4 |  |  |

*Please be sure to conduct the measurements at stable temperature conditions.

1. Use the permanent marker to label four beakers Conductivity 23 μS/cm, 100 μS/cm, 447 μS/cm, and 2764 μS/cm.
2. Use the graduated cylinder to pour 100 mL of each conductivity standard solution into the corresponding labeled beaker (be sure to rinse the cylinder with deionized water when switching standards).
3. Dip the pH and conductivity sensor tip into the 23 μS/cm beaker.
4. Once the conductivity number on the sensor monitor stabilizes (you will see “READY” on the screen), record the measured pH in **Data Table 2** in the 23 μS/cm row.
5. Rinse the sensor tip with distilled water.
6. Repeat Step 12-14 for the 100 μS/cm standard-containing beaker.
7. Repeat Step 12-14 for the 447 μS/cm standard-containing beaker.
8. Repeat Step 12-14 for the 2764 μS/cm standard-containing beaker.
9. Cleanup: Use a soft cloth or test tube cleaning brush, Isopropyl alcohol, and distilled water to loosen solids or oils from all laboratory equipment after the completion of an experiment. Thoroughly rinse the items with distilled water and allow them to air dry on clean kimwipes.

**Data Table 2**. Conductivity calibration table.

| Standard Conductivity (*y*_i_, μS/cm) | Measured Conductivity (*x*_i_, μS/cm) | *Measured Temperature (°C) |
| --- | --- | --- |
| 23 |  |  |
| 100 |  |  |
| 447 |  |  |
| 2764 |  |  |

*Please be sure to conduct the measurements at stable temperature conditions.

### Part 2: Generating Standard Curves (45 min)

1. From **Data Table 1**, plot measured pH as *x*_i_ and standard pH as *y*_i_.
2. Compute the standard curve fit and compute the true *y* values.

| *x*_i_, measured | *y*_i_, standard | *y*, true/adjusted |
| --- | --- | --- |
|  | 4 |  |
|  | 7 |  |
|  | 10 |  |

Model Answer: y = 0.97_9_ x + 0.08_0_

| *x*_i_, measured | *y*_i_, standard | *y*, true/adjusted |
| --- | --- | --- |
| 4.01 | 4 | 4.00_4_ |
| 7.06 | 7 | 6.99_0_ |
| 10.14 | 10 | 10.00_5_ |

1. From **Data Table 2**, plot measured conductivity as *x*_i_ and standard conductivity as *y*_i_.
2. Compute the standard curve fit and compute the true *y* values.

| *x*_i_, measured | *y*_i_, standard | *y*, true/adjusted |
| --- | --- | --- |
|  | 23 |  |
|  | 100 |  |
|  | 447 |  |
|  | 2764 |  |

**Model Answer = y = 0.91_2_ – 2.83_8_**

| *x*_i_, measured | *y*_i_, standard | *y*, true/adjusted |
| --- | --- | --- |
| 58 | 23 | 55.71_1_ |
| 114 | 100 | 106.67_0_ |
| 458 | 447 | 420.35_1_ |
| 3040 | 2764 | 2774.10_0_ |

## **Exercise 2 - pH and Conductivity Assessment on Water Samples**

### Sample Preparation (10 min)

Two water samples will be examined in this exercise: ultrapure water and tap water. Be sure to stock at least 100 mL of each water sample by following the steps below.

1. Put on your gloves.
2. Use the permanent marker to label one polyethylene laboratory bottle Tap Water and another bottle Ultrapure Water.
3. Write your name and date on **Data Table 3**.
4. Fill the Tap Water tube with cold water from the faucet.
5. Record the source, well or municipal, and the area where collected as the description in **Data Table 3.**

NOTE: You can prepare the filtered tap water sample by filtering the tap water through a sterile syringe connected to a sterile 25 mm diameter polycarbonate filter with 0.2 µm pore size (VWR, 28145-477 and 309653). Please prepare ≥ 100 mL of filtered tap water.

1. Gather ultrapure water, fill the bottle, and record the brand and area where manufactured as the description in **Data Table 3**.
2. Add any observations regarding the samples (if any).

| Your Name: __________________  Date: _______________________  **Data Table 3: Source of Water Samples** | |  |
| --- | --- | --- |
|  | Tap Water (Sample 1) | Ultrapure Water (Sample 2) |
| Source |  |  |
| Description |  |  |
| Observation |  |  |

**Note: Test all samples as soon as possible after they have been gathered. All remaining samples can be used for other complementary analyses if needed.**

### Part 1: pH and Conductivity Tests (20 min)

1. Put on your gloves.
2. Use the permanent marker to label two beakers **Tap Water** and **Ultrapure Water**.
3. Use the graduated cylinder to pour 100 mL of your **Tap Water** sample into the associated labeled beaker (be sure to rinse the cylinder with deionized water when switching standards).
4. Dip the pH and conductivity sensor tip into the **Tap Water** beaker.
5. Once the pH and conductivity numbers on the sensor monitor stabilize (you will see “READY” on the screen), record the measured numbers, including pH, conductivity, and temperature, in **Data Table 4** in the **Tap Water** row. If necessary, you can apply the standard curve adjustment to your values.
6. Rinse the sensor tip with distilled water.
7. Repeat Step 3-6 for the ultrapure water-containing beaker.
8. Cleanup: Use a soft cloth or test tube cleaning brush, Isopropyl alcohol, and distilled water to loosen solids or oils from all laboratory equipment after the completion of an experiment. Thoroughly rinse the items with distilled water and allow them to air dry on clean kimwipes.

**Data Table 4. Bulk water properties**

| Sample | pH (*y*_i_) | Conductivity (*y*_i_, µS cm^-1^) | *Measured Temperature (°C) |
| --- | --- | --- | --- |
| Tap water |  |  |  |
| Ultrapure water |  |  |  |

*Please be sure to conduct the measurements at stable temperature conditions.

###
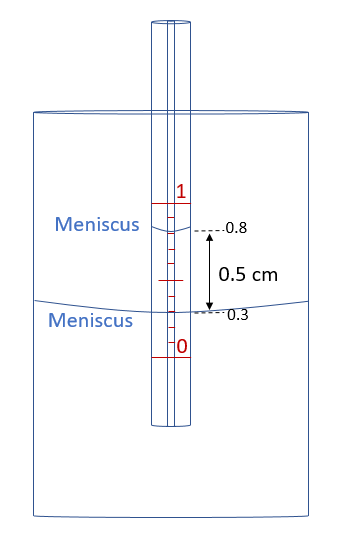
Part 2: Surface Tension Test (30 min)

1. Put on your gloves.
2. Clean the capillary thoroughly with isopropyl alcohol and rinse with distilled water.
3. Place 100 mL of **Tap Water** sample in the cylinder.
4. Wet the hole in the rubber stopper and the outside of the capillary tube with a small amount of sample to be analyzed.
5. Insert the capillary into the rubber stopper and push the rubber cap straight down until approximately 1” of the capillary extends beyond the top of the cap.
6. Place the rubber cap with capillary into the cylinder and adjust the position of the capillary into the aqueous sample to locate the surface of the sample between the “0” line and the “1” line.
7. Use the syringe pump to draw air out of the cylinder until you observe that air bubbles are pulled out of the capillary.
8. Removed the syringe from the tubing to allow the sample liquid to reach equilibrium inside the capillary tube.

**Figure 2**. Distance between menisci.

1. Measure the distance between the meniscus inside the capillary tube and the meniscus of the cylinder as illustrated in **Fig. 2**. This process should be repeated three times, followed by creating a positive pressure inside the capillary tube to pull liquid from the top of the tube.
2. The distance between the meniscus inside the tube and the meniscus inside the cylinder should be again measured after the syringe is disconnected. This process should also be repeated three times.
3. Use the average from Step 9 and 10 to calculate the surface tension using Equation 10 as follows:

$y= \frac{1}{2}\cdot h\cdot r\cdot d\cdot g$ [10]

y = surface tension (dyn cm^-1^ or mN m^-1^)

h = distance between menisci (cm)

r = radius of capillary (= 0.025 cm)

d = density of sample at measuring temperature (≈ 0.998 g cm^-3^)

g = acceleration due to gravity at the location of measurement (≈ 980.7 cm s^-2^)

1. Record the calculated number in **Data Table 5** in the **Tap Water** row.
2. Repeat Step 2-12 for the ultrapure water sample.
3. Cleanup: Use a soft cloth or test tube cleaning brush, Isopropyl alcohol, and distilled water to loosen solids or oils from all laboratory equipment after the completion of an experiment. Thoroughly rinse the items with distilled water and allow them to air dry on clean kimwipes.

**Data Table 5. Surface Tension properties**

|  | Surface Tension (*y*_i_, dyn cm^-1^) | *Measured Temperature (°C) |
| --- | --- | --- |
| Tap water |  |  |
| Ultrapure water |  |  |

*Please be sure to conduct the measurements at stable temperature conditions.

## **ASSESSMENT**

### Before You Proceed

- Did you complete all of the required exercises in this lesson? If not, please return to the previous section to finalize your work.
- Are you confident that you’ve achieved the learning objectives listed below? If not, please review the INTRODUCTION content and your responses to the exercise activities.

### Learning Objectives

- Describe the bulk properties of water samples.
- Explain pH numerically
- Relate water properties to atmospheric ice nucleation.

### Exercise Review Questions

#### Question 1

**Is there a relationship between pH and electrical conductivity?**

Model Answer: The students’ answers will vary. As discussed, pH is the measurement of a specific ion (i.e. hydrogen). In contrast, electrical conductivity is a non-specific measurement of the concentration of both positively and negatively charged ions within a sample. Thus, the relationship between pH and conductivity is that the presence of hydrogen ions present may impact the pH level of a substance, and the presence of these ions will probably influence the conductivity level. However, hydrogen ions make up only a small part of the ion concentration measured by a conductivity meter. So given this, the relevance of the relationship depends on the presence of hydrogen in comparison with other concentrations of non-pH determining ions.

#### Question 2

**Discuss what bulk properties have an influence on ice nucleation activity by looking at the table below.**

|  | **pH** | **Conductivity (µS cm^-1^)** | **Surface Tension (dynes cm^-1^)** | ***T* (ᵒC)** | ***IN activity*** |
| --- | --- | --- | --- | --- | --- |
| **Unfiltered-tap water** | 7.5 ± 0.1 | 859.8 ± 23.7 | 73.8 ± 0.5 | 19.3 ± 0.9 | **High** |
| **Filtered-tap water** | 8.0 ± 0.3 | 1006.3 ± 47.6 | 73.0 ± 0.7 | 18.7 ± 1.5 | **Middle** |
| **HPLC water** | 5.6 ± 0.3 | 22.4 ± 16.4 | 75.9 ± 2.1 | 18.9 ± 1.1 | **Low** |

Model Answer: The students’ answers will vary. See SI Sect. S2 - 2.1 for discussion of the student feedback.

#### Question 3

**How do your Data Tables 4 and 5 compare to the model results provided in Question 2? If the difference is observed, explain what could be the source of the data deviation. If necessary, use the table below to fill in your outcome for the comparison.**

| **Sample** | **pH** | **Conductivity (µS cm^-1^)** | **Surface Tension (dynes cm^-1^)** | ***T* (ᵒC)** |
| --- | --- | --- | --- | --- |
| **Sample 1** |  |  |  |  |
| **Sample 2** |  |  |  |  |

Model Answer: The students’ answers will vary. See SI Sect. S2 – 1.1 for discussion of the student answers.

#### Question 4

**Do you observe the suppression of surface tension in tap water as compared to ultrapure water? If yes, explain what this suppression tells you.**

Model Answer: The students’ answers will vary. See SI Sect. S2 – 1.1 for discussion of the student answers.

### Competency Review Questions

#### Question 1

**Calculate the pH for a specific [H^+^]. Calculate pH given [H^+^] = 3.5 x 10^-5^ M.**

Model Answer:

pH = -log_10_[H^+^]
pH = -log_10_(3.5 x 10^-5^)
pH = 4.46

#### Question 2

**Find the pH of a 0.0012 M HCl (hydrochloric acid) solution.**

Model Answer: Hydrochloric acid is a strong acid that dissociates according to a 1:1 molar ratio into hydrogen cations and chloride anions (i.e., 100% ionization). Hence, the concentration of hydrogen ions is exactly the same as the concentration of the acid solution. Here, the hydronium ion concentration is 0.0012 M, and therefore we get:

pH  =  - log (0.0012) = - ( - 2.92) = 2.92

#### Question 3

**What is the hydrogen ion concentration in a solution that has a pH of 9.31?**

Model Answer:

9.31 = - log [H^+^]
- 9.31 = log [H^+^]
[H^+^] = 10^-9.31^ = 4.90 x 10^-9^ M

#### Question 4

**Calculate the concentration of hydroxides in the water of pH = 7 at 25 °C.**

Model Answer:

In neutral pH water, the ratio of H+ and OH- is 1:1.

[H+] = 1.0 x 10^-7 M

$K_{w}=\left[ H^{+} \right] \left[ {OH}^{-} \right]=1.0\times{10}^{-7}\left[ {OH}^{-} \right] at-25 ^{\circ}C$

[OH-] = 1.0 x 10^-7 M.

#### Question 5

**Which of the following factor affect the conductivity in the water?**

- temperature
- the concentration of ions in the solution
- type of substance dissolved in water
- all of these

The correct answer is all of these.

#### Question 6

**What is the average conductivity of seawater? Seawater has a high conductivity because salts act as a [ ].**

The correct answer is 50 mS/cm; an electrical conductor (not an insulator).

## **REFERENCES**

- Gianino, C.: Measurement of surface tension by the dripping from a needle, Phys. Educ. 2006, 41, 440–444,  https://doi.org/10.1088/0031-9120/41/5/010, 2006.
- Harris, D.C.: Exploring Chemical Analysis Second Edition, W. H. Freeman Company, New York, NY, pp. 73–90, 2000.
- Hiranuma, N. et al.: Influence of surface morphology on the immersion mode ice nucleation efficiency of hematite particles, Atmos. Chem. Phys., 14, 2315–2324, https://doi.org/10.5194/acp-14-2315-2014, 2014.
- Li, K. et al.: Investigating the effects of solid surfaces on ice nucleation, Langmuir, 28, 10749–10754, https://doi.org/10.1021/la3014915, 2012.
- Lin, J. J. et al.: Effects of surface tension time-evolution for CCN activation of a complex organic surfactant. Environ. Sci.: Process. Impacts, 22, 271–284, https://doi.org/10.1039/c9em00426b, 2020.
- OpenStax College, pH Scale, availbale at

https://commons.wikimedia.org/wiki/File:216_pH_Scale-01.jpg, 2013, last visited on February 13, 2023.

- Schindler, P. W. and Stumm, W.: The Surface Chemistry of Oxides; Hydroxides, and Oxide Minerals in Aquatic Surface Chemistry: Chemical Processes at the Particle-Water Interface John Wiley and Sons, New York, NY, pp.83–110, 1987.
- USGS: Surface Tension and Water, 2019, available at https://www.usgs.gov/special-topics/water-science-school/science/surface-tension-and-water, last visited on June 2, 2022.
- Whale, T. F. et al.: The enhancement and suppression of immersion mode heterogeneous ice-nucleation by solutes, Chem. Sci., 9, 4142–4151, https://doi.org/10.1039/C7SC05421A, 2018.

**Copyright Statement** Characterization of bulk water properties by Elise K. Wilbourn, Sarah Alrimaly, Holly Williams, Jacob Hurst, Gregory P. McGovern, Todd A. Anderson, and Naruki Hiranuma is marked with CC0 1.0 Universal Creative Commons license. To view a copy of this license, visit http://creativecommons.org/publicdomain/zero/1.0
