# Supplementary material for: Integrated Science Teaching in Atmospheric Ice Nucleation Research: Immersion Freezing Experiments
Source: J Chem Educ. 2023 Mar 8;100(4):1511–22. doi: 10.1021/acs.jchemed.2c01060 (PMC10100551; doi:10.1021/acs.jchemed.2c01060)
Supplement: Supplementary file 1 — ed2c01060_si_001.zip [file ed2c01060_si_001.zip › SI_Files/SI_Sect_S1_Module_2_WT_CRAFT.docx]

**Supporting Information:**

**Integrated Science Teaching in Atmospheric Ice Nucleation Research:**

**Immersion Freezing Experiments**

Elise K. Wilbourn^1,♦^, Sarah Alrimaly^1,♦^, Holly Williams^1^, Jacob Hurst^2^, Gregory P. McGovern^2^,

Todd A. Anderson^3^, and Naruki Hiranuma^1,^*

^1^Dept. of Life, Earth, and Environmental Sciences, West Texas A&M University, Canyon, TX, 79016

^2^ Dept. of Chemistry and Physics, West Texas A&M University, Canyon, TX, 79016

^3^ Dept. of Environmental Toxicology, Texas Tech University, Lubbock, TX, 79416

^♦^These authors equally lead and contributed to this work

*Corresponding author ([nhiranuma@wtamu.edu](mailto:nhiranuma@wtamu.edu))

# **Title: Module 2 - Freezing of water droplets and ice-nucleating particles**

Estimated Completion Times: 480 min

Introduction 45 min

Experimentation 360 min

Assessment 75 min

Prerequisites: Chemistry I, College Statistics, and College Algebra (and/or Pre-Calculus).

Target course level: an upper-level undergraduate or graduate-level course in Environmental Science/Chemistry.

## **INTRODUCTION**

### Introduction Summary

The introduction section contains background reading materials to prepare you for performing the WT-CRAFT exercises in this lesson.

### Learning Objectives

- Define homogeneous and heterogeneous freezing and their atmospheric relevance.
- Describe how temperature impacts ice nucleation.
- Explain the source, abundance, and spatial distribution of ice-nucleating particles (INPs) in the atmosphere.
- Understand the basic theory of homogeneous freezing of water droplets.

### Test Your Knowledge

1. Higher tropospheric altitude typically has [ ] temperature; thereby, more ice nucleation generally occurs in air aloft than near-ground altitude when sufficient water vapor is available.
   - Lower
   - Higher
2. Without ice-nucleating particles, [ ] can promote ice cloud formation in the atmosphere.
   - Homogeneous freezing
   - Heterogeneous freezing
3. True or False: Atmospheric ice-nucleating particle concentrations measured over continental sites are in general higher than marine-predominant sites.
   - True
   - False
4. [ ] is one of the major sources of INPs due to its abundant yearly emission rate and relatively high ice nucleation efficiency.
   - Pollen
   - Volcanic ash
   - Soil dust
   - Soot

### Answer Key: 1. Lower 2. Homogeneous freezing 3. True 4. Soil dust

### Subsection 1: Ice nucleation mechanisms

INPs provide a surface on which water vapor and/or cloud droplets deposit and freeze. This type of ice formation in the presence of INPs is known as **heterogeneous freezing**. In the absence of INPs, the formation of atmospheric ice particles follows the process of **homogeneous freezing**, which requires cloud droplets to be supercooled to the temperature (*T*) of -32 °C and below (depending on the pure water droplet size) to form ice crystals. Though our knowledge regarding INPs remains insufficient, there have been advances in understanding the different modes of heterogeneous ice nucleation (IN) in the atmosphere in the last few decades. **Figure 1** illustrates the current understanding of known ice nucleation mechanisms in the atmosphere. As seen, INPs can change the altitude of ice cloud formation and nucleation/freezing pathways. For instance, **deposition nucleation** is induced by the direct deposition of water vapor onto an INP’s surface and ice embryo formation on the surface under ice supersaturation conditions. Recently, some studies have argued that deposition nucleation could be interpreted as **pore condensation and freezing**. The presence of water in pores of mineral materials and the resulting inverse Kelvin effect cause an instantaneous water saturation condition in the confined space, allowing the water to freeze even at water sub-saturated ambient conditions. Amongst various IN paths, perhaps the most important mode is **immersion freezing**, accounting for more than 85% of atmospheric heterogeneous freezing. This process starts with the formation of a cloud droplet followed by freezing due to an INP immersed in the supercooled droplet. In addition, past studies have identified other modes of heterogeneous nucleation, such as **condensation freezing** (i.e., ice forms as water vapors condense on an INP at subzero), **contact freezing** (i.e., an INP promotes freezing when it contacts a supercooled droplet from the outside), and **inside-out evaporation freezing** (i.e., freezing of an immersed INP in contact with the droplet surface from the inside). These modes are relatively less relevant in mixed-phase clouds.


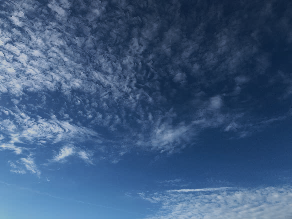

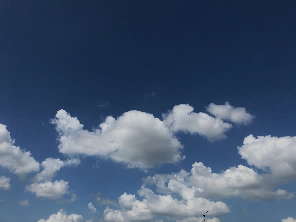

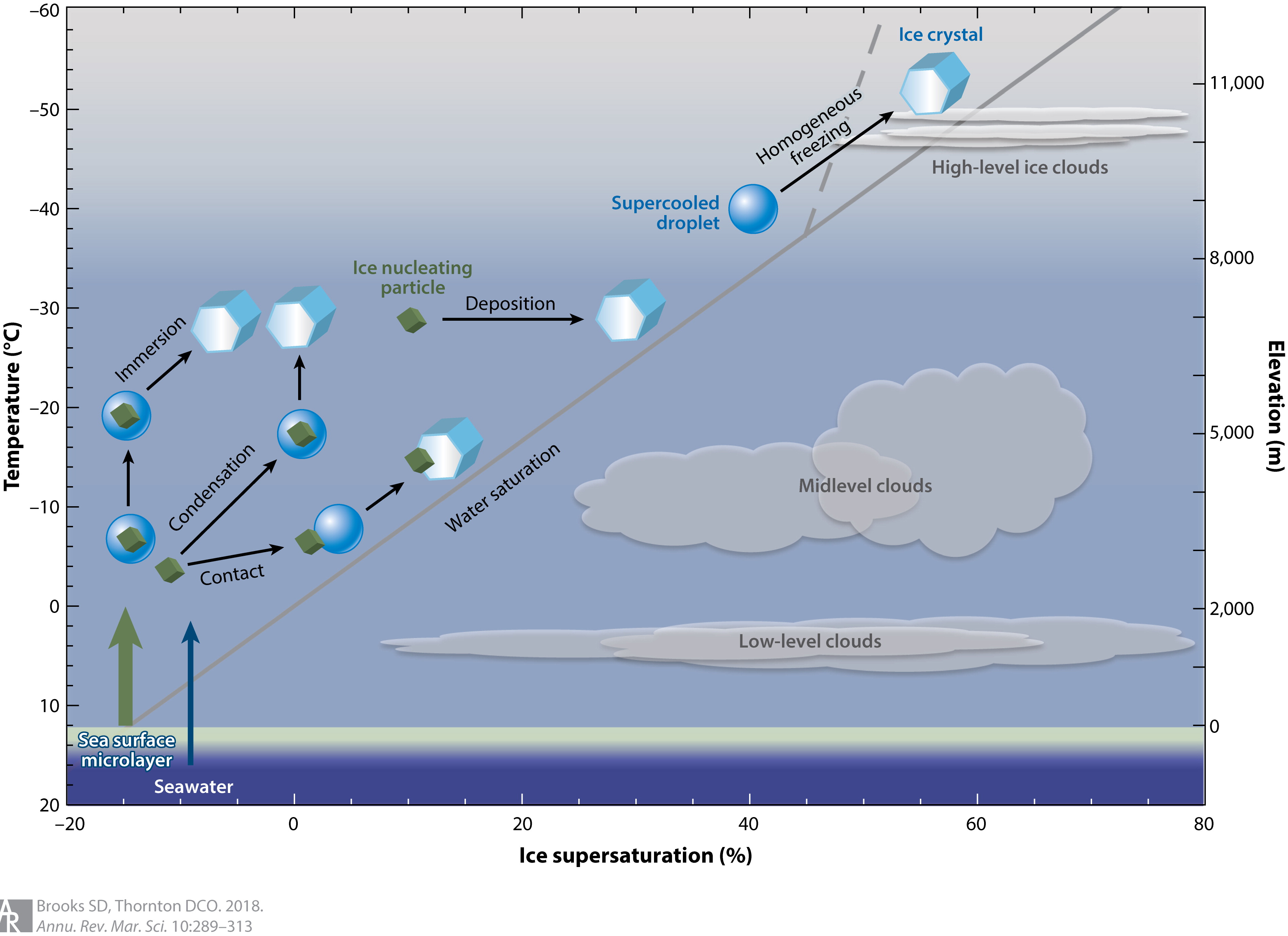


**Figure 1**. Atmospheric ice nucleation mechanisms as a function of temperature and elevation. Adapted from Brooks and Thornton (2018).

### Subsection 2: Importance of immersion freezing

INPs greatly influence cloud properties, especially in mixed-phase clouds, which are typically observed in the altitude range of 2000 m to 9000 m above ground level. Out of all heterogeneous ice-nucleation modes, immersion freezing is the most dominant mode of ice formation in mixed-phase clouds. Previous field studies showed that the formation of cloud droplets is a prerequisite for ice formation in mixed-phase clouds (de Boer et al., 2011), thus highlighting the importance of immersion nucleation. In one model simulation study, different cloud types such as orographic, stratiform, and deep-convective systems were simulated and analyzed for different freezing modes under various polluted conditions (Hande and Hoose, 2017). This study demonstrates that immersion freezing is predominant IN mode under various simulated circumstances, accounting for 85 to 99%, while other IN paths play a less significant role. Similarly, the importance and predominance of supercooled liquid droplets as a prerequisite of atmospheric ice formation is reported in one field observation study based on radar and lidar observations of clouds over the U.K. at temperatures relevant to immersion freezing (Westbrook and Illingworth, 2011). Another study led by Cui et al. (2006) also showed that immersion freezing is the primary mode of ice formation with minor amounts of deposition nucleation in the early stages of cloud development. While contact freezing may be a highly efficient ice formation path, a previous study led by Phillips et al. (2007) showed that it is a negligible mode in the simulated mixed-phase cloud conditions. Due to the importance and dominance of immersion freezing, the instrument that will be used in this lesson essentially simulates immersion freezing.

### Subsection 3: Ice-nucleating particle abundance

Ambient INPs represent a small population of aerosol particles deriving from either marine or continental sources and catalyze ice crystal formation in supercooled clouds (i.e., ice supersaturated and subzero temperature conditions). Below **Fig. 2** displays a compilation of previous immersion-mode INP concentration (*n*_INP_) data over the major oceans. **Figure 3** shows a map of all previous marine *n*_INP_ measurements published through early 2021. The associated data for INP ranges as a function of temperature and study locations, as well as references, are summarized in **SI** **Sect. S7** along with the data readme files. As seen, immersion-active INP concentrations can range from 10^-5^ to 10^3^ L^-1^ in marine-predominant sites across the world at temperatures above approximately -35 °C. Likewise, **Fig. 4** shows the previously reported ambient *n*_INP_ of soil dust and a compilation of other continental field-measured *n*_INP_ from across the world in the temperature range between -5 °C and -25 °C. The global reference field *n*_INP_ data from Kanji et al. (2017) are also shown at their temperature points (i.e., -15, -20, and -25 °C). It is apparent that the continental *n*_INP_ spectra below -15 °C are located above or overlapping with the upper bound of *n*_INP_ spectra from previous marine-predominant sites (**Fig. 2**). The highest *n*_INP_ observed in the continental site is ≈ 10^4^ L^-1^, which is about an order magnitude higher than the upper range of marine *n*_INP_, indicating that absolute INPs per unit volume at continental sites are much higher than previously investigated marine INP sources.


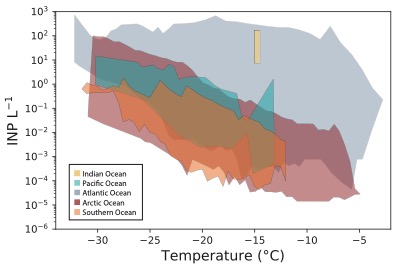


**Figure 2.** The measured range of INP concentrations for each of the five major oceans, based on studies of immersion freezing capabilities of marine-derived INPs. A reference list of studies and locations is available in **SI Sect. S7**.


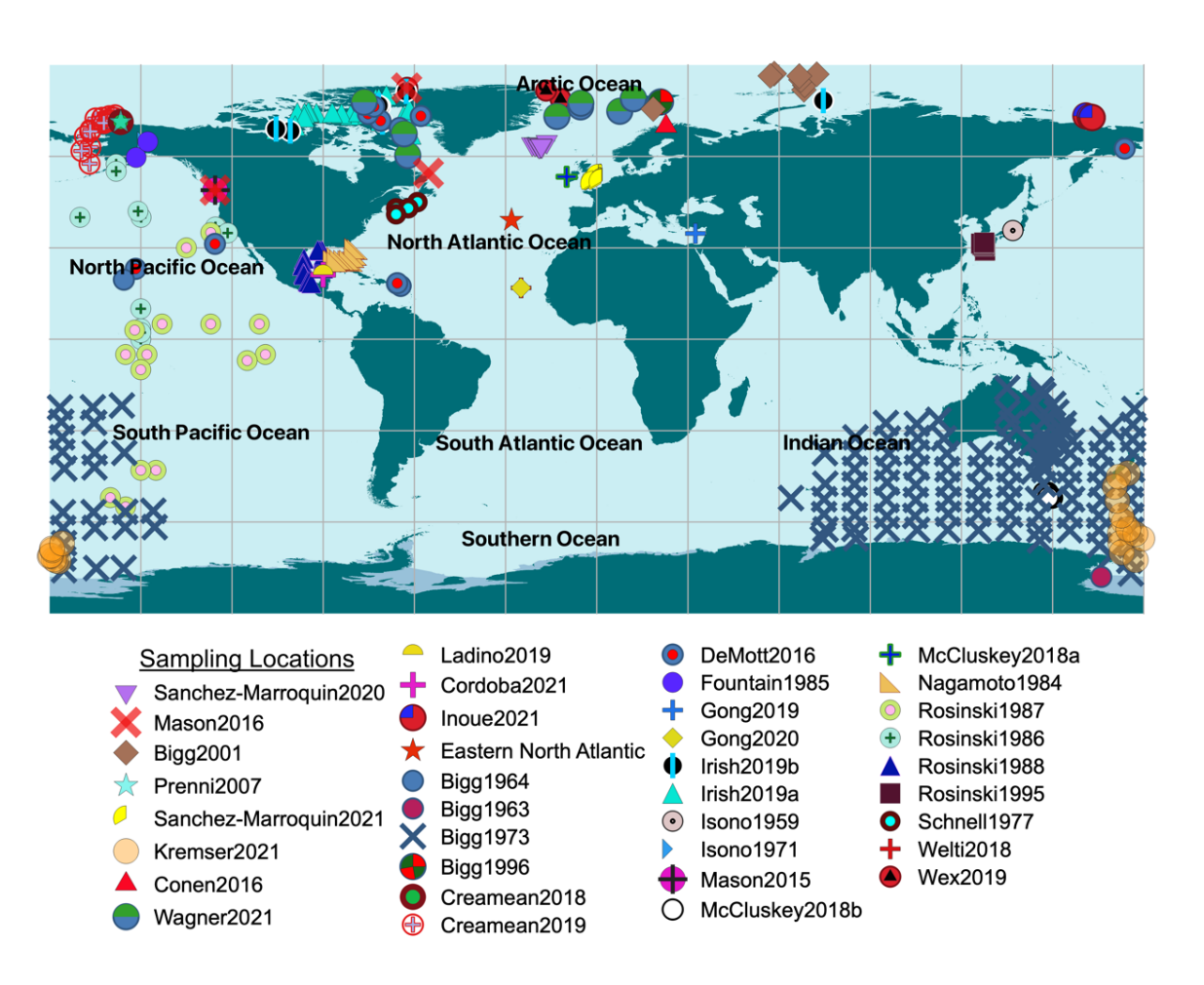


**Figure 3.** All locations where ambient marine INPs were sampled (legend and reference list match **Fig. 2**).


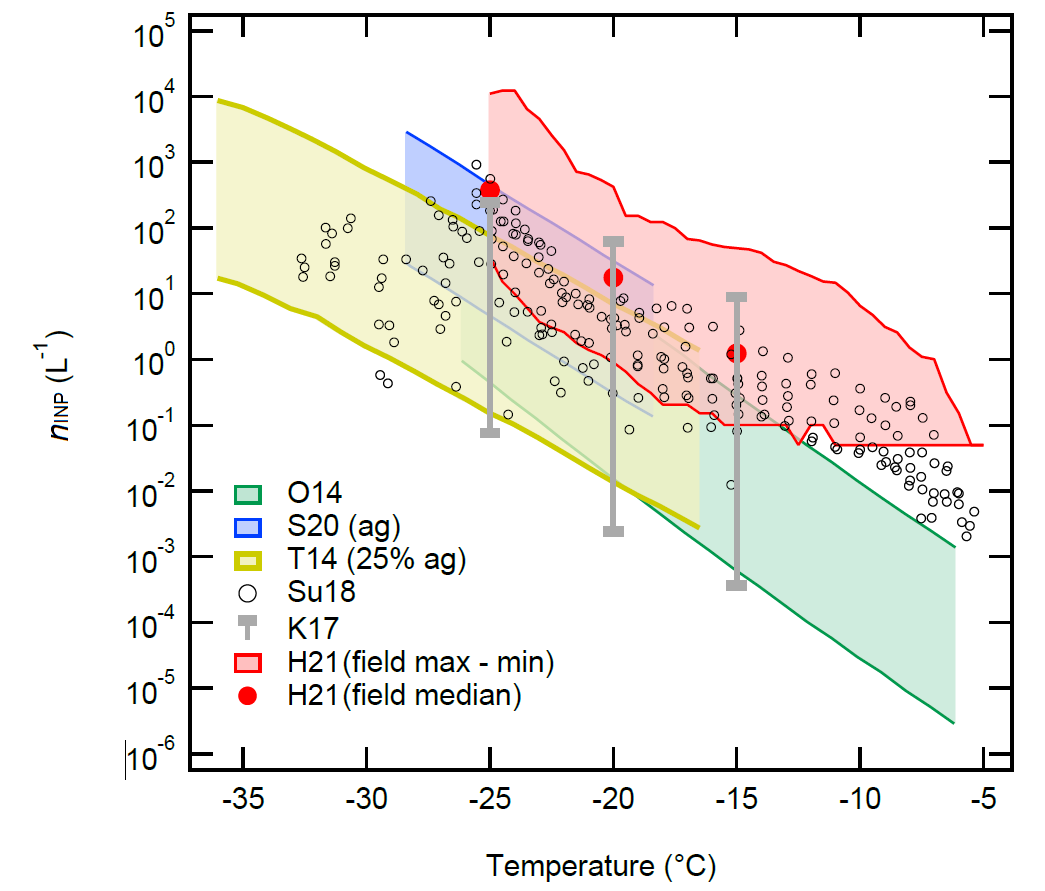


**Figure 4**. The n_INP_(T) spectra of soil dust and continental aerosol particles as a function of temperature. This figure is adapted from Hiranuma et al. (2021) - [CC BY license](https://creativecommons.org/licenses/by/4.0/) (Creative Commons Attribution 4.0 International license). Reference data include O’Sullivan et al. (2014 Fig. 9; O14), Steinke et al. (2020 Fig. 3; S20), Tobo et al. (2014 Fig. 6b; T14), Suski et al. (2018 Fig. 1a-d; Su18), Kanji et al. (2017 Fig. 1-10; K17), and Hiranuma et al. (2021 Fig. 9). Note that we display the maximum and minimum at -15, -20, and -25 °C of K17 in comparison to our estimation.

### Subsection 4: Source of ice-nucleating particles

One of the biggest challenges in the INP research field is the fact that we do not yet understand all INP sources and the ambient abundance of INPs worldwide. Thus, it is an imperative task to identify and characterize any perturbation sources that alter INP abundance and cloud-phase feedback. The sources of atmospheric INPs are diverse as they emerge naturally and also through human activities, adding complexities to our comprehensive understanding of their impacts. **Figure 5** shows an overview of both anthropogenic and natural INP sources.

In particular, with annual emission rates of 1000 to 4000 teragrams, soil dust represents a major source of both total particle loading and INPs in the atmosphere. The resulting radiative forcing directly exerted by mineral dust is estimated to range from -0.3 to +0.1Wm^-2^. Therefore, dust slightly contributes to the direct cooling effect of aerosols. However, our understanding of the influence of the dust burden upon overall climate forcing, including its secondary effect on cloud albedo, remains highly uncertain, in part due to the absence of accurate INP representations in atmospheric models. Thus, the effective radiative forcing effect of airborne dust on current climate predictions remains unresolved. Furthermore, a large amount of INPs is globally anticipated from agricultural activities because agricultural practices represent a substantial dust emission source, accounting for up to 25% of total global dust emission.

During jet spray and wave breaking, marine sea spray aerosols (SSAs) can be produced. SSAs are known to act as both cloud condensation nuclei and INPs. The bubble-bursting process then aerosolizes the organic material found in the underlying seawater and the sea surface microlayer, forming organic- and salt-rich SSA. SSAs containing marine organic material are a well-known but globally minor source of INPs.

Proteinaceous particulate matter from biological sources is known to act as very active INPs with freezing temperatures as high as -1.8 °C. Thus, ice nucleation measured/observed at > -15 °C is generally attributed to proteinaceous INPs. However, the ambient quantity of biological INPs is substantially sparse as compared to other INP types, and their overall impact on cloud and precipitation modulations is still under debate.


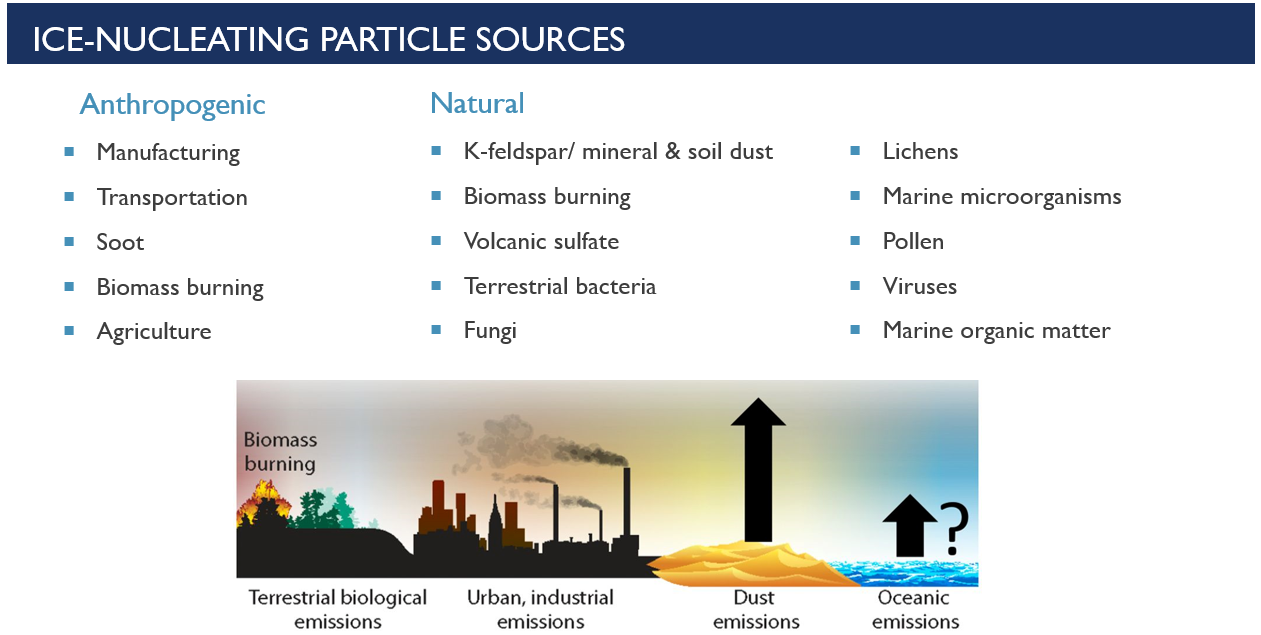


**Figure 5**. Overview of potential ice-nucleating particle sources, adapted from Seinfeld et al. (2016).

### Subsection 5: Homogeneous freezing of monodisperse and polydisperse droplets

Atmospheric freezing of droplets is a stochastic process (Möhler et al., 2013; Duft and Leisner, 2004). Assuming all droplets have the same freezing probability per unit time, the freezing rate of droplets ($\frac{dn}{dt}$) can be expressed:

$\frac{dn}{dt}=-nj_{freeze}=-nJ_{v}V$ [1]

$\frac{dn}{n}=-J_{v}V dt$ [2]

in which, *n* is the number concentration of droplets, *V* is the volume of each **monodisperse** droplet, *j*_freeze_ is the rate coefficient for droplet freezing (i.e., the fraction of droplets to freeze per unit time), and *J*_v_ is the volume nucleation coefficient (often simply termed nucleation rate). Next, the number concentration of droplets at time *t*, *n*(*t*), can be estimated by integrating Eqn. [2]. Starting with *n*_0_ droplets at time *t*_0_, the *n*(*t*) value decreases with time according to:

$\int_{t_{0}}^{t} \frac{dn}{n}=-\int_{t_{0}}^{t} J_{v}Vdt$ [3]

$\ln\frac{n(t)}{n_{0}}=-J_{v}V(t-t_{0})$ [4]

or, with *t*_0_ = 0

$n(t)=n_{0}\exp\left(-J_{v}Vt\right)$ [5]

If each droplet that freezes forms one ice crystal, we get:

 [6]

where *n*_ice_(*t*) is the number concentration of ice crystals at time t, *J*_v_ acts as a constant according to Duft and Leisner (2004, See **Fig. 6** below).

For **polydisperse** droplets, one has to sum up ice formation in size bins i:

$n_{ice}(t)=\sum_{i} n_{ice,i}(t)=\sum_{i} n_{0,i}\left(1-\exp\left(-J_{v}V_{i}t\right)\right)$ [7]

With linear approximation for short time periods ∆*t* with low freezing probability (i.e., *J_v_V_i_*D*t* <<1), we find $\exp\left( -J_{v}V_{i}\Delta t \right)\approx1-J_{v}V_{i}\Delta t$. Therefore, the increase in ice crystal concentrations (∆*n*_ice_) can be defined as:

$\Delta n_{ice}=\sum_{i} n_{i}V_{i}J_{v}\Delta t=v J_{v} \Delta t$ [8]

In which *ν* is the total liquid droplet volume concentration at the time of nucleation.


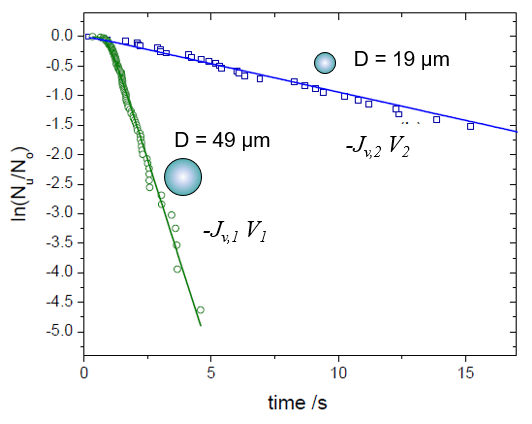


**Figure 6.** Relationship between ln($\frac{n(t)}{n_{0}}$) and *t* to define *J*_v_ *V*. Note *J*_v,1_ ((2.75 ± 0.15) × 10^6^ cm^-3^ s^-1^) ≈ *J*_v,2_ ((2.85 ± 0.15) × 10^6^ cm^-3^ s^-1^). This figure is in the courtesy of Ottmar Möhler. N_u_ denotes number of unfrozen droplets (= *n*(*t*)) and N_0_ is equivalent to *n*_0_.

## **EXPERIMENTATION**

### Experimentation Summary

This section will guide you through competency-building exercises. You will be challenged with applying the knowledge you gained through the introduction section to complete the activities successfully.

### Learning Objectives

- Analyze two types of water samples for freezing ability and efficiency as a function of temperature.
- Relate results to abundance/sources of contamination.

### Hypothesis Formulation

1. Do you expect the deionized (DI) water or tap water to freeze more efficiently at a higher temperature? Explain your hypothesis.
2. What would you expect to see with the frozen fraction curve and associated error if we employed the 0.05 °C min^-1^ cooling instead of the 1 °C min^-1^ cooling?

### Materials

Read through the procedures listed in the exercises on the next pages before beginning. Then, purchase and gather all of the materials listed below and begin Exercise 1. Materials required to complete Exercise 1 include:

| **Qty.** | **Item** | **Manufacturer, Model, Estimate $ (Total ~$3,500)** |
| --- | --- | --- |
| 1 | [A] Cryogenic Refrigerator* | Scinics, CP-80CP, *$2,620.00* |
| 1 | [B] 12” LED Ring Light | e.g., Sunpak, VL-LED-162-12RL, *$50* |
| 1 | [C] Webcam | Logitech, C270, *$50.00* |
| 1 | [D] Laptop PC** | e.g., Dell Inspiron 13-7000, 8GB RAM, *$450* |
| 1 | [E] Surface Temperature Digital Thermometer | e.g., Circuit Specialists, DTM0501, *<$5* |
| 1 | Micropipette | Eppendorf, EW-24505-18, *$296.00* |
| 2 | Micropipette Tip | Eppendorf, 022491415, *$78.00* for 480 tips |
| 1 | Vaseline Petroleum Jelly | Covidien, 8884430300, *$1.95* ($ 9.75 for 5 sets) |
| 1 | Chemically Inert Spatula | VWR, 82027-512, *$28.90* |
| 1 | Sterile Syringe Filter | VWR, 28145-477, $75 |
| 1 | 50 mL Luer-Lok Syringe | VWR, 309653, $95 for 40 |
| 2 | Sterile polypropylene tube with cap <15 mL volume | e.g., VWR, 10026-076, *$1.20* ($200 for 500 tubes) |

*can be substituted with any one of similar cryocooler systems if necessary - see Table 1 of Miller et al. (2021); **can be an ordinary laptop but one compatible with Logitech C270 webcam and the associated video recording function

The following materials might be available at your laboratory or the university physical plant. Note that the WT-CRAFT system can be easily constructed by simply assembling Items [A] to [H] as explained in Exercise 1.

| **Qty.** | **Item** |
| --- | --- |
| 1 | [F] Acryl board (W x L x H = 20 cm x 20 cm x <0.5 cm) |
| 1 | [G] Aluminum board (W x L x H = 11 cm x 9.2 cm x <0.5 cm) |
| 1 | [H] Aluminum board (W x L x H = 1.5 cm x 9.2 cm x <0.5 cm) |
| 1 | Tap water (<15 mL) |
| 1 | Distilled water |
| 1 | Laboratory grade DI water |
| 3 | Pair of gloves |
| 1 | Isopropyl alcohol |
| 1 | Kimwipes |
| 1 | Aluminum tape |


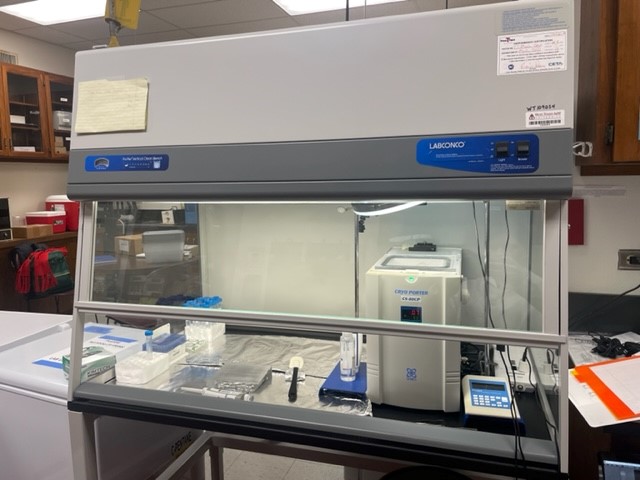

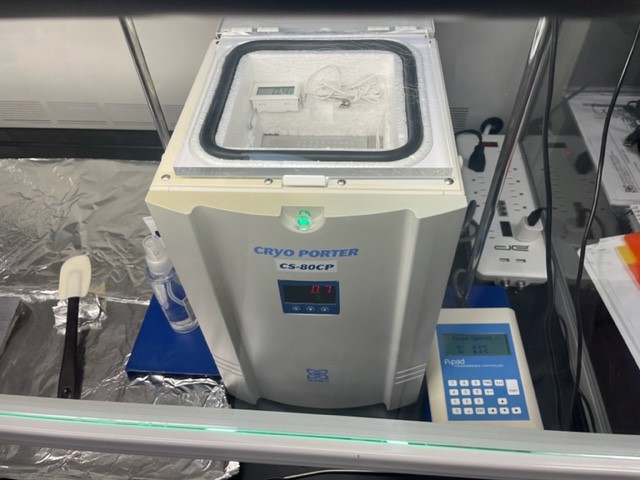


OVERVIEW OF THE APPARATUS IN A CLEAN HOOD

CRYO-CHAMBER

- Cryogenic refrigerator with an inset program control pad [A]
- Acrylic board [F]

**F**

**A**


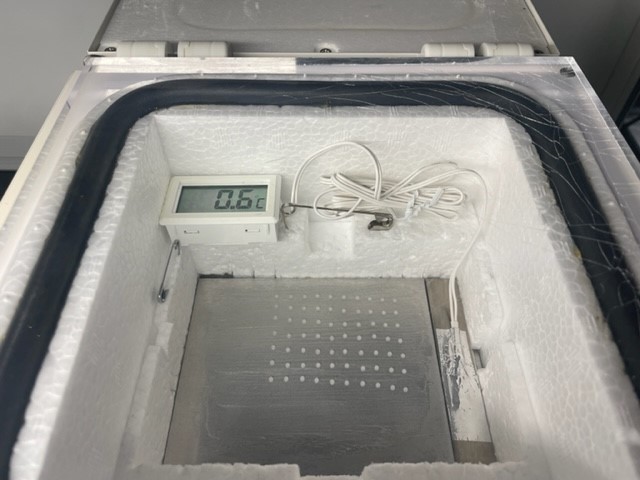

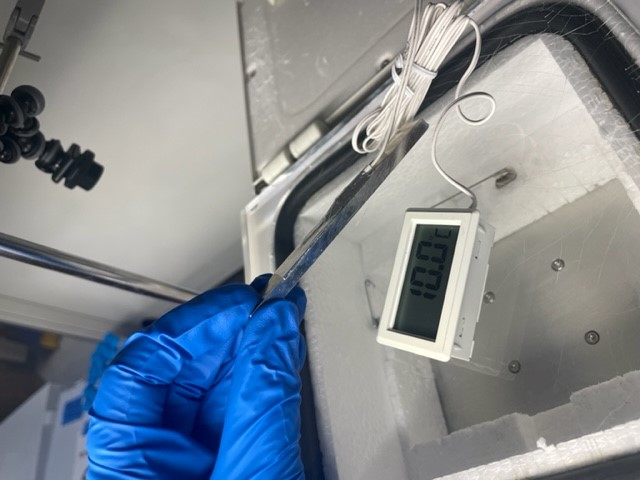


INTERIOR SETUP

- Temperature display [E] and cord secured by safety pins to prevent the display to fall onto aluminum plate during cooling.
- Temperature probe affixed to plate using aluminum tape [H] – NOTE: Crucial to completely seal probe tip as any air infiltration will provide inaccurate readings.
- Aluminum board to host 70 droplets for the immersion freeing experiment [G].

TEMPERATURE PROBE ON SMALL ALUMINUM PLATE [E], [H]

**G**

**H**

**E**


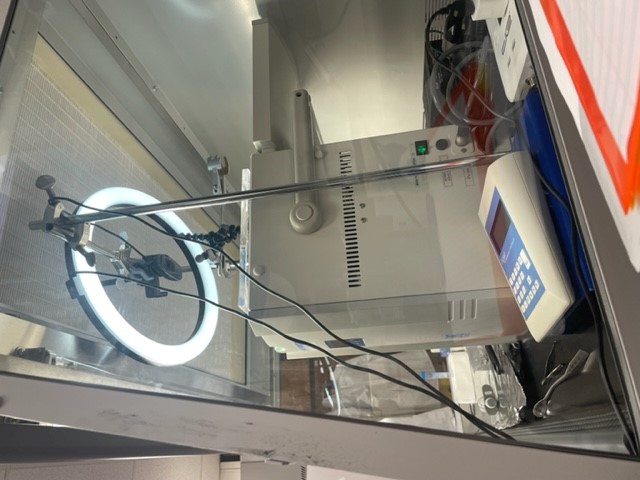

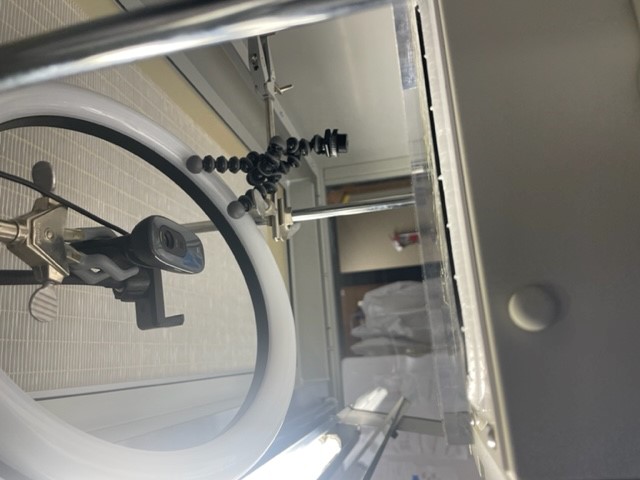

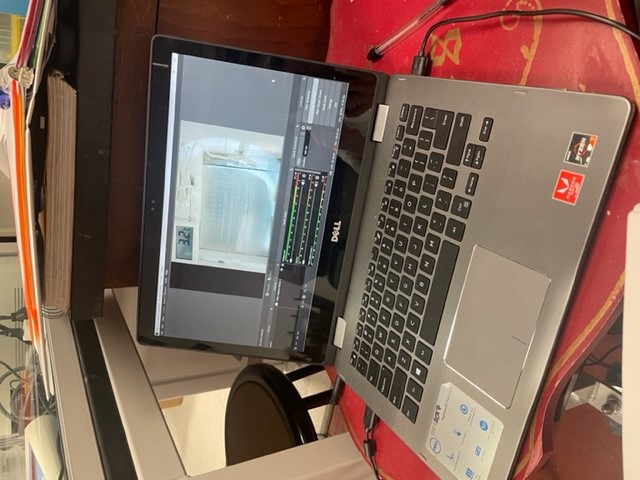


**C**

**B**

RING LIGHT [B] WEB CAMERA [C] VIDEO RECORDING LAPTOP [D]


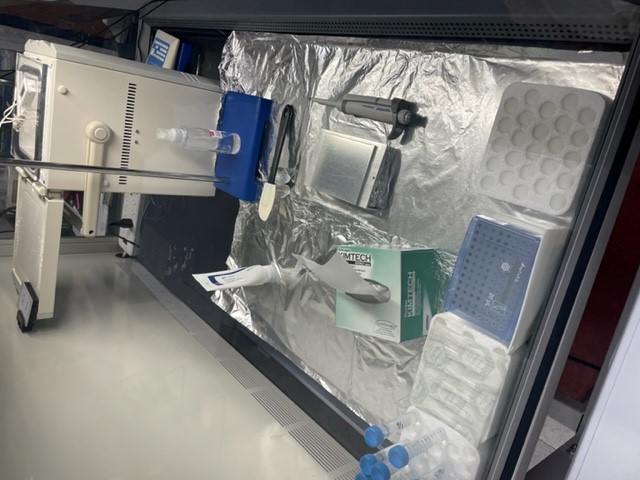

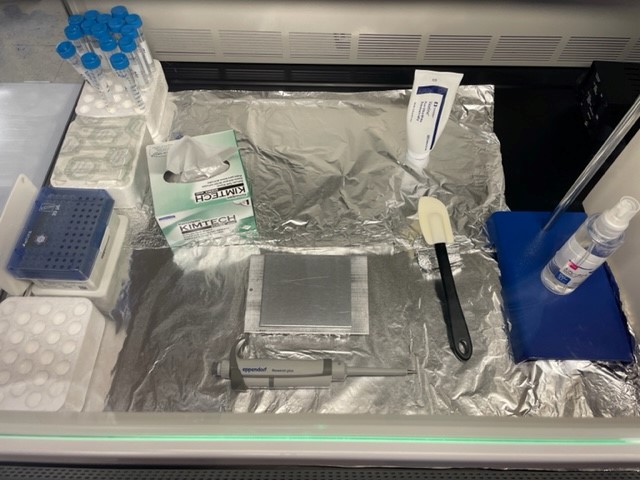


CLEAN HOOD INTERIOR OVERVIEW

WORK SURFACE

Supplies:

- Bench scraper (upright next to pipette tips), pipette tips, test tube Styrofoam rack, kimwipes, pipetting stage, aluminum plate, pipettor, spatula with ‘kickstand’ (made of folded aluminum foil; to prevent contaminating work surface with Vaseline), tube of Vaseline, alcohol spray

Notes:

- Work surface has been protected with sheets of aluminum foil
- We replace foil about every 2 weeks as it gets torn from pipetting stage being adjusted

### Safety

- Safety in a lab is the first priority and everyone’s responsibility. You should always follow safety protocols, be responsible, take precautions, and you must be able to recognize hazards and potential dangers. Plan and think ahead when conducting experiments to be aware of any safety concerns at all times. Carefully assess your situation during experiments to anticipate and avoid potential hazards and dangers. Putting those precautions into practice in a lab is important to practice a safe work environment.
- Safety Data Sheets (SDSs) will be provided for all chemicals used in this module. SDSs provide information about physical properties, health risks, fire explosion data, and other important information associated with these chemicals. Before handling or using a chemical, you should refer to the SDS for that chemical.
- It is your responsibility to inform the instructor in writing of any health conditions that may prevent you from safely using a chemical (pregnancy, autoimmune deficiency, etc.). It is also the responsibility of the student to report any spill or problems found while storing or using a chemical. If you are unsure about a chemical, always ask. If you see any unsafe conditions, notify your instructor immediately. If you are unsure about the proper and safe operation of any piece of equipment, ask your instructor for proper instructions. All injuries, spill of materials, and unsafe conditions must be reported to the instructor immediately.
- Any pregnant students, or students planning to become pregnant, should consult their health care provider to determine what, if any, additional precautions are needed based on their individual situation. While the university cannot mandate that the student notify that they are pregnant or are planning to become pregnant, the university strongly recommends that students provide notification so appropriate steps can be taken to ensure the health of both parent and child.
- ALWAYS wear appropriate PPE including safety goggles. Eyesight protection will help prevent harm from chemical spills, splashes, debris, as well as flying objects while conducting scientific experiments. If glasses are worn make sure your glasses fit in the safety goggles. Everyday glasses are NOT a substitution for safety goggles due to there being no protection due can still enter your eye from the side.
- Protect yourself with appropriate lab attire: Wear appropriate fitting clothes that fully cover your arms and legs, a lab coat, closed-toed shoes that fully cover your feet, and keep hair tied back away from your face, flames, chemicals, and other experiment instruments.
- NEVER leave an ongoing experiment unattended.
- A clean and neat workspace is important for safety. Protect work surfaces by using the appropriate tools to prevent spillages, use fume hoods with certain chemicals, paper towels easily accessible for cleanup, and organize the area.
- Being aware of your surroundings in a lab is important for physical activity. Danger can occur when not aware of your surroundings. Consult your physician before any physical activity is done in the lab i.e. climbing ladders, moving heavy materials, and using heavy machinery. **Always** ask for help if the task needs more than one person to complete. Have an ample amount of space so that objects are not falling off shelves or counters.
- Laboratories carry different types of chemicals some are potentially toxic. DO NOT ingest any chemicals in the lab at any time. Eating, drinking, or tasting any lab chemicals is not permitted especially in home kitchens used as a lab.
- A local regulatory agency can be contacted to follow the correct procedure in handling disposal waste and how to discard any chemicals (reagents) or dissection specimens.
- Newspaper and paper towels can be useful to dispose of non-chemical experiment items in a household garbage can after an experiment is complete. Make sure all garbage cans are secured and covered so they cannot be accessed by children or animals.
- A first Aid kit will be available and easily accessible in the lab for emergency treatment of minor injuries. The kit should be well-stocked with formal and informal laboratories. Replacement kits can be purchased at any grocery or pharmacy store.
- Lab safety equipment will be in all labs. Eye washing stations are used to flush the face and eye areas only. The rule of thumb is to flush your eyes (hold eyelids open) or face when the contamination is unknown for 20 minutes. 15-20 minutes for moderate to severe irritation and chemicals that may cause acute toxicity through the face or eyes. 30 minutes for corrosives, and 60 minutes for (sodium, potassium, or calcium hydroxide) strong alkalis. If irritation persists seek medical attention immediately from a physician. An informal laboratory may use the eyewash station as a substitute for a hand-held wand or sink faucet.
- Formal labs have safety showers that are designed to wash your complete body in the event of coming in contact with a chemical spill on your skin. Turn on the shower and undress to make sure the water is reaching every part of your body. This is not the time to be modest. You want to make sure that all clothing that has the chemicals isn’t still on your skin. Don’t rub! Rubbing your skin can lead to the chemicals going deeper into your skin causing more damage. Flush yourself for 15 minutes and make sure you seek medical attention from a physician for severe burns etc.

## **Exercise 1 - Testing Water Suspensions**

In this exercise, you will test the freezing efficiency of two water suspensions. You will analyze each sample for frozen fraction and associated 95% confidence interval (CI95%).

### WT-CRAFT Setup and Start-up (50 min)

1. Using Items [A] to [H] listed in the materials section, assemble the WT-CRAFT system. A general schematic overview of the WT-CRAFT is shown in **Fig. 7**. You can also refer to the video instruction of WT-CRAFT (https://www.youtube.com/watch?v=DU0ImxbgE_U&t=1s) to reproduce the experimental setup.
2. Cover Cryogenic Refrigerator with an acrylic top plate.
3. Switch on the Main Power Switch of the Cryogenic Refrigerator.
4. On the refrigerator control panel menu, select 2: Fixed Program and press [ENT]. Note: ‘Fixed Program’ means that the machine will stay at a constant temperature based on what is typed into the system.
5. Type 5.0 on the control panel keypad for the SV control, and press [START]. This process will cool WT-CRAFT to 5.0 ℃ in 15-20 min (varying depending on ambient temperature).
6. The temperature of WT-CRAFT should be resting at 5.0 ℃ prior to any experiments.
7. Turn on an LED ring light.
8. Open/Turn on Laptop PC.
9. Start the video recording software (e.g., OBS Studio). Check webcam visuals for clear imaging.


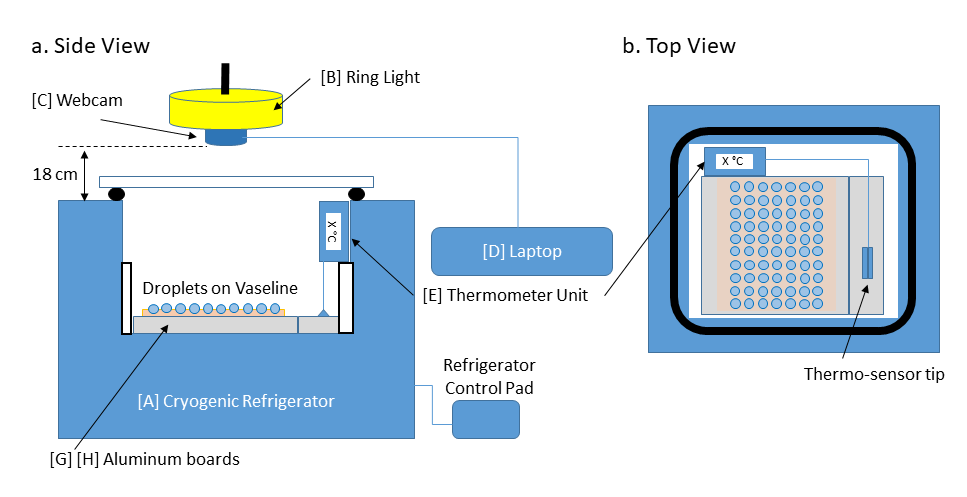


**Figure 7**. Schematic layout of the WT-CRAFT system used for immersion freezing (a. Top & b. Side View). This figure is adapted from Tobo (2016) - [CC BY license](https://creativecommons.org/licenses/by/4.0/) (Creative Commons Attribution 4.0 International license).

### Sample Preparation (10 min)

1. Put on your gloves.
2. Use the permanent marker to label one 15 mL polypropylene tube Tap Water and a second tube DI Water.
3. Write your name and date on **Data Table 1**.
4. Fill the Tap Water tube with cold water from the faucet.

NOTE: You can alternatively prepare the filtered tap water sample by filtering the tap water through a sterile syringe connected to a sterile 25 mm diameter polycarbonate filter with a 0.2 µm pore size (VWR, 28145-477 and 309653). Please prepare 15 mL of filtered tap water.

1. Record the source, well or municipal, and the area where collected as the description in **Data Table 1**.
2. Gather deionized and tap water samples, fill the tubes, and record the brand and area where manufactured as the description in **Data Table 1**.
3. You will add more information in the experiment/observation section later on, so leave this row blank for now.

**Note: Test all samples as soon as possible after they have been gathered. All remaining samples can be used for other complementary analyses if needed.**

| Your Name: __________________  Date: _______________________  **Data Table 1: Source of Water Samples** | |  |  | |
| --- | --- | --- | --- | --- |
|  | Tap Water | DI Water | |  |
| Source |  |  | |  |
| Description |  |  | |  |
| Experiment/Observation |  |  | |  |

### Part 1: Tap Water Freezing Experiment (60 min)

1. Grab your **tap water sample** tube.
2. Put on your gloves.
3. Clean the aluminum base plate thoroughly with Isopropyl alcohol and Kimwipes.
   1. Spray Isopropyl onto Kimwipe and proceed to clean the plate.
   2. Always end with wiping in one direction (like you would clean your car window).
   3. Be sure to check for any left-over artifacts, such as Vaseline or Kimwipe debris.
4. Clean plastic spatula with Isopropyl alcohol and Kimwipe.
   1. Spray Isopropyl to cover the spatula.
   2. Use Kimwipe to clean in one direction – making sure to clean around the raised edges.
   3. Inspect spatula for any leftover Vaseline.
5. Using the spatula evenly spread Vaseline on an aluminum base plate. Tip: Avoid spreading to the edge of the plate – leave ~1/2”.
6. Adjust the micropipette using the top turn dial for desired droplet volume (3 µL). Press down on the appropriate pipette tip with a gentle force to engage the tip to the pipette.
7. Using a micropipette, transfer 70 of 3 µL droplets from your water sample tube onto the aluminum plate. Tip: Inspect droplets from a distance to make sure of even distribution, droplet size, and amount of droplets. When placing droplets, be sure to space appropriately where droplets do not contact each other (~ 1 mm apart).
8. Remove the WT-CRAFT acrylic cover.
9. Carefully place the aluminum base plate, hosting 70 droplets, at an angle into CRAFT (~ 45-60°) then align to the left edge.
10. Using an aluminum tape, place the thermometer on a small aluminum board [H], and the sensor bar is placed parallel to the bottom on Styrofoam (**Fig. 6**).
11. Fit the aluminum strip with the thermometer attachment on the right edge pressing down along the sensor to confirm complete contact.
12. Rotate the temperature sensor gauge to face upward towards the web camera.
13. Cover the WT-CRAFT with an acrylic cover to isolate the air in the refrigerator from ambient air. This process also helps minimize ambient contamination and droplet evaporation during the immersion freezing experiment.
14. Allow for the aluminum plate and sensors to cool to [5.0 ℃].
15. Check the camera [B] and the LED light [C] on a computer screen to confirm proper focus on 70 droplets.
16. On the refrigerator control panel menu, press [ESC] to return to the main menu. The main menu will have two options:

1: Program Control

2: Fixed Program

1. Choose ‘1: Select Program’ and [ENT].
2. Use the cursor to choose the desired program (i.e., 1 °C per min cooling from 5 °C to -35 °C) and press [ENT]. Note: With a cooling rate of 1 °C min^-1^, this experiment will take **40 min**.
3. Press [START] to begin the pre-programmed cooling experiment.
   1. Green flashing dot will appear, indicating cooling on Cryogenic Refrigerator.
   2. Green number on Cryogenic Refrigerator is the set temperature.
   3. Red number on Cryogenic Refrigerator is the current temperature.
4. On your laptop PC [D], start recording the webcam video and run until all droplets get frozen or the refrigerator temperature reaches -35 °C.

After the experiment is completed, stop recording the video and follow the procedures below.

1. Label the recorded video file with the date and in letter sequence (e.g., 20180315b – means the second video recorded on the 15^th^ of March in 2018). Note: the letter after represents the video of the day (e.g., a = 1st, b = 2nd, etc.).
2. Record the measurement time stamp (date and time of starting to ending), the volume of droplets, number of droplets, examined temperature range, and any notable experimental artifacts (e.g., droplet size inconsistent etc.) in the experiment/observation section of **Data Table 1**.
3. Press [ESC] on the refrigerator control panel to return to the main menu.
4. Select 2: Fixed Control [ENT].
5. Type 5 on the keypad for the SV control, and press [START].
6. Wait until the aluminum plate warms to 5.0 ℃ and All droplets are unfrozen.
7. Remove the acrylic WT-CRAFT cover.
8. Pick up the aluminum plate with the temperature sensor.
9. Slide your aluminum base plate to left and pick it up from WT-CRAFT.
10. Cover CRAFT with an acrylic plate.

### Part 2: DI Water Freezing Experiment (60 min)

Grab your **DI water sample** tube, and repeat Step 2-30 for it.

## **Exercise 2 – Data Analysis of Freezing Properties of Water Suspensions**

### Part 1: Tap Water Freezing Data Analysis (30 min)

1. Find your tap water experiment video file, then you will conduct the video analysis to fill out **Data Table 2**.
2. Open the video file of your water experiment, go to the video, and press play.
3. From 0 °C onward, stop the video every 0.5 °C to record the number of the frozen droplet (*n*_frozen_).
   1. In the second column starting from the left, the numbers you will be inputting will go here.
   2. The numbers you will record in this column are the number of droplets frozen as a function of temperature at a temperature step of 0.5 °C. This should coordinate with the temperature on the left of it. Insert zeros if you see none of the droplets frozen.
   3. **Data Table 2** is set up for every half a degree. To get the entirety of the degree, there will be an overshoot. The initial start of half a degree will start a 0.1 of the temperature you are on. Then you continue to watch until the very end of 0.5 - right up until it turns to 0.6.
4. Repeat the *n*_frozen_ data entry until the end of the video or you see *n*_frozen_ = 70.
5. Fill in the next two columns of **Data Table 2**. You will need to calculate the Frozen Fraction (*FF*) and the nucleus concentration in suspension (*C*_INP_(*T*), L*^−^*^1^ water) as a function of temperature.
   1. *FF* represents a number of frozen droplets at a given temperature, *n*_frozen_(*T*), scaled to a total number of examined droplets in a single experiment (*n* = 70) for every 0.5 °C.
   2. The *C*_INP_(*T*) value can be computed to represent the ice nucleus concentration in suspension (L*^−^*^1^ water) as a function of temperature as:

$$C_{INP}\left( T \right)= - \frac{\ln\left( 1-FF\left( T \right) \right)}{V_{d}} [9]$$

in which, *V_d_* is the volume of the sample in each droplet (3 *µ*L or 0.003 mL) for WT-CRAFT.

1. Fill in the next two columns of **Data Table 2**. You will need to calculate upper/lower 95% binomial sampling confidence intervals (CI95%).
   1. *FF* represents a number of frozen droplets at a given temperature, *n*_frozen_(*T*), scaled to a total number of examined droplets in a single experiment (*n* = 70) for every 0.5 °C.
   2. The *C*_INP_(*T*) value can be computed to represent the nucleus concentration in suspension (L*^−^*^1^ water) as a function of temperature as:

$$Upper CI95\%\left( T \right)= \frac{1}{1+\frac{z_{\alpha/2}^{2}}{n}}\left( FF+\frac{z_{\alpha/2}^{2}}{2n}+z_{\alpha/2} \sqrt{\frac{FF\left( 1-FF \right)}{n}+\frac{z_{\alpha/2}^{2}}{4n^{2}}} \right) [10]$$

$$Lower CI95\%\left( T \right)= \frac{1}{1+\frac{z_{\alpha/2}^{2}}{n}}\left( FF+\frac{z_{\alpha/2}^{2}}{2n}-z_{\alpha/2} \sqrt{\frac{FF\left( 1-FF \right)}{n}+\frac{z_{\alpha/2}^{2}}{4n^{2}}} \right) [11]$$

in which, The $z_{\alpha/2}$ value is 1.96 for a 95% confidence interval, which is determined by the (1-α/2) quantile of the standard normal distribution (a=0.05 for a 95% CI).

### Part 2: DI Water Freezing Data Analysis (30 min)

Repeat 2-6 using your DI water video/data.

**Data Table 2: Freezing properties of water samples**

| ***T*** | Tap Water | | | | |  | DI Water | | | | |
| --- | --- | --- | --- | --- | --- | --- | --- | --- | --- | --- | --- |
|  | ***n*_frozen_** | **FF** | **C_INP_** | **Upper CI95%** | **Lower CI95%** |  | ***n*_frozen_** | **FF** | **C_INP_** | **Upper CI95%** | **Lower CI95%** |
| °C | # | Unitless | L^-1^ | + L^-1^ | - L^-1^ |  | # | Unitless | L^-1^ | + L^-1^ | - L^-1^ |
| 0.0 |  |  |  |  |  |  |  |  |  |  |  |
| -0.5 |  |  |  |  |  |  |  |  |  |  |  |
| -1.0 |  |  |  |  |  |  |  |  |  |  |  |
| -1.5 |  |  |  |  |  |  |  |  |  |  |  |
| -2.0 |  |  |  |  |  |  |  |  |  |  |  |
| -2.5 |  |  |  |  |  |  |  |  |  |  |  |
| -3.0 |  |  |  |  |  |  |  |  |  |  |  |
| -3.5 |  |  |  |  |  |  |  |  |  |  |  |
| -4.0 |  |  |  |  |  |  |  |  |  |  |  |
| -4.5 |  |  |  |  |  |  |  |  |  |  |  |
| -5.0 |  |  |  |  |  |  |  |  |  |  |  |
| -5.5 |  |  |  |  |  |  |  |  |  |  |  |
| -6.0 |  |  |  |  |  |  |  |  |  |  |  |
| -6.5 |  |  |  |  |  |  |  |  |  |  |  |
| -7.0 |  |  |  |  |  |  |  |  |  |  |  |
| -7.5 |  |  |  |  |  |  |  |  |  |  |  |
| -8.0 |  |  |  |  |  |  |  |  |  |  |  |
| -8.5 |  |  |  |  |  |  |  |  |  |  |  |
| -9.0 |  |  |  |  |  |  |  |  |  |  |  |
| -9.5 |  |  |  |  |  |  |  |  |  |  |  |
| -10.0 |  |  |  |  |  |  |  |  |  |  |  |
| -10.5 |  |  |  |  |  |  |  |  |  |  |  |
| -11.0 |  |  |  |  |  |  |  |  |  |  |  |
| -11.5 |  |  |  |  |  |  |  |  |  |  |  |
| -12.0 |  |  |  |  |  |  |  |  |  |  |  |
| -12.5 |  |  |  |  |  |  |  |  |  |  |  |
| -13.0 |  |  |  |  |  |  |  |  |  |  |  |
| -13.5 |  |  |  |  |  |  |  |  |  |  |  |
| -14.0 |  |  |  |  |  |  |  |  |  |  |  |
| -14.5 |  |  |  |  |  |  |  |  |  |  |  |
| -15.0 |  |  |  |  |  |  |  |  |  |  |  |
| -15.5 |  |  |  |  |  |  |  |  |  |  |  |
| -16.0 |  |  |  |  |  |  |  |  |  |  |  |
| -16.5 |  |  |  |  |  |  |  |  |  |  |  |
| -17.0 |  |  |  |  |  |  |  |  |  |  |  |
| -17.5 |  |  |  |  |  |  |  |  |  |  |  |
| -18.0 |  |  |  |  |  |  |  |  |  |  |  |
| -18.5 |  |  |  |  |  |  |  |  |  |  |  |
| -19.0 |  |  |  |  |  |  |  |  |  |  |  |
| -19.5 |  |  |  |  |  |  |  |  |  |  |  |
| -20.0 |  |  |  |  |  |  |  |  |  |  |  |
| -20.5 |  |  |  |  |  |  |  |  |  |  |  |
| -21.0 |  |  |  |  |  |  |  |  |  |  |  |
| -21.5 |  |  |  |  |  |  |  |  |  |  |  |
| -22.0 |  |  |  |  |  |  |  |  |  |  |  |
| -22.5 |  |  |  |  |  |  |  |  |  |  |  |
| -23.0 |  |  |  |  |  |  |  |  |  |  |  |
| -23.5 |  |  |  |  |  |  |  |  |  |  |  |
| -24.0 |  |  |  |  |  |  |  |  |  |  |  |
| -24.5 |  |  |  |  |  |  |  |  |  |  |  |
| -25.0 |  |  |  |  |  |  |  |  |  |  |  |
| -25.5 |  |  |  |  |  |  |  |  |  |  |  |
| -26.0 |  |  |  |  |  |  |  |  |  |  |  |
| -26.5 |  |  |  |  |  |  |  |  |  |  |  |
| -27.0 |  |  |  |  |  |  |  |  |  |  |  |
| -27.5 |  |  |  |  |  |  |  |  |  |  |  |
| -28.0 |  |  |  |  |  |  |  |  |  |  |  |
| -28.5 |  |  |  |  |  |  |  |  |  |  |  |
| -29.0 |  |  |  |  |  |  |  |  |  |  |  |
| -29.5 |  |  |  |  |  |  |  |  |  |  |  |
| -30.0 |  |  |  |  |  |  |  |  |  |  |  |
| -30.5 |  |  |  |  |  |  |  |  |  |  |  |
| -31.0 |  |  |  |  |  |  |  |  |  |  |  |
| -31.5 |  |  |  |  |  |  |  |  |  |  |  |
| -32.0 |  |  |  |  |  |  |  |  |  |  |  |
| -32.5 |  |  |  |  |  |  |  |  |  |  |  |
| -33.0 |  |  |  |  |  |  |  |  |  |  |  |
| -33.5 |  |  |  |  |  |  |  |  |  |  |  |
| -34.0 |  |  |  |  |  |  |  |  |  |  |  |
| -34.5 |  |  |  |  |  |  |  |  |  |  |  |
| -35.0 |  |  |  |  |  |  |  |  |  |  |  |

### Cleanup

- Dispose of the used pipets and falcon tubes in the trash.
- Use a soft cloth or test tube cleaning brush, mild dishwashing detergent, and warm water to loosen solids or oils from all laboratory equipment after the completion of an experiment. Thoroughly rinse the items with distilled water and allow them to air dry on clean paper towels or a clean dishtowel.
- Store all clean, dry equipment in a safe location for future use.

## **ASSESSMENT**

### Before You Proceed

- Did you complete all of the required exercises in this lesson? If not, please return to the previous section to finalize your work.
- Are you confident that you’ve achieved the learning objectives listed below? If not, please review the INTRODUCTION content and your responses to the exercise activities.

### Learning Objectives

- Visualizing the freezing ability of two water samples and the associated uncertainties.
- Define ice nucleation mechanisms.
- Describe why immersion freezing is atmospherically relevant.
- Explain the abundance and source of ice-nucleating particles.
- Relate water quality to sources of the experimental artifact.

### Exercise Review Questions

#### Question 1

1. **Based on your Data Table 2, plot your *FF* and *C*_INP_ ± CI95% as a function of temperature for two water samples. Find *FF*_50%_ and *T_FF_*_50_. HINT:** frozen fraction (*FF*) represents a number of frozen droplets at a given temperature, *n*_frozen_(*T*), scaled to a total number of examined droplets in a single experiment (*n* = 70) for every 0.5 °C. You can use a sigmoidal curve fitting on your data or visually inspect the curves to find a 50% frozen fraction (*FF*_50_) and corresponding temperature (*T_FF_*_50_) for each water type.
2. **Why is it important to represent the experimental uncertainty of the immersion freezing experiment with binomial (statistical) errors rather than a consistent systematic error?**

Model Answer: The student answers will vary but should address the points discussed in SI Sect. S2 – 1.2.2.

#### Question 2

**How do the test results compare for your tap water and the other water sample? Explain which sample you would consider “pure” based on Data Table 2 and your plot from Question 1.**

Model Answer: The student answers will vary but should address the points discussed in SI Sect. S2 – 1.2.2.

#### Question 3

**Is your tap water containing more INPs than illite NX suspension? Explain why.**

Model Answer: The student answers will vary but should correlate their data in Data Table 2 with the main manuscript Figure 4. For instance, if the temperature corresponding to a 50% frozen fraction (*T_FF_*_50_) of a tap water sample is higher than that of illite NX, the student's answer should address the possibility of more INP in tap water than the illite NX suspension.

#### Question 4

**List three potential sources of an experimental artifact based on your data recorded in Data Table 1. What can we do to minimize these potential contaminations?**

Model Answer: The student answers will vary but should address the points discussed in SI Sect. S2 – 2.2.

### Competency Review Questions

#### Question 5

Explain the difference between homogeneous freezing and heterogeneous freezing.

Model Answer:

- **heterogeneous freezing:** Ambient ice formation in the presence of INPs - INPs provide a surface on which water vapor and/or cloud droplets deposit and freeze.
- **homogeneous freezing:** Ambient ice formation in the absence of INPs - the formation of atmospheric ice particles requires cloud droplets to be supercooled to the temperature (*T*) of -32 °C and below (depending on the pure water droplet size) to form ice crystals.

#### Question 6

Define at least four major pathways of ambient heterogeneous freezing.

Model Answer:

- **Deposition nucleation**: the deposition nucleation is induced by the direct deposition of water vapor onto an INP’s surface and ice embryo formation on the surface under ice supersaturation conditions.
- **Pore condensation and freezing**: The presence of water in pores of mineral materials and the resulting inverse Kelvin effect cause an instantaneous water saturation condition in the confined space, allowing the water to freeze even at water sub-saturated ambient conditions.
- **Immersion freezing**: This process starts with the formation of a cloud droplet followed by freezing due to an INP immersed in the supercooled droplet.
- **Condensation freezing**: ice forms as water vapors condense on an INP at subzero.
- **Contact freezing**: an INP promotes freezing when it contacts a supercooled droplet from the outside.
- **Inside-out evaporation freezing**: freezing of an immersed INP in contact with the droplet surface from the inside.

#### Question 7

Discuss the importance of immersion freezing as compared to other ice nucleation pathways.

Model Answer: See Introduction Subsection 2. The student answers vary but they should reference that one model simulation study (Hande and Hoose, 2017) demonstrates that immersion freezing is predominant IN mode under various simulated circumstances, accounting for 85 to 99%, while other IN paths play a less significant role.

Reference

Hande, L. B. and Hoose, C.: Partitioning the primary ice formation modes in large eddy simulations of mixed-phase clouds, Atmos. Chem. Phys., 17, 14105–14118, <https://doi.org/10.5194/acp-17-14105-2017>, 2017.

#### Question 8

Fill in the blank: Immersion-active INP concentrations can range from [ ] L^-1^ in marine-predominant sites across the world at temperatures above approximately -35 °C.

1. 10^-5^ to 10^3^
2. 10^-5^ to 10^4^
3. 1 to 100
4. 0.1 to 10^3^

Model Answer: A

#### Question 9

Looking at **Figs. 2 and 3**, it is apparent that there is still a lack of marine *n*_INP_ data regardless of the effort made over 70 years. Identify the region where future work should focus on increasing the spatial spread of sampling.

The student answers may vary, but the model answer is e.g., Northern part of Indian Ocean, South Atlantic Ocean, Oligotrophic ocean in general.

#### Question 10

Compare **Fig. 2** and **Fig. 4**, and (a) describe general trends of continental and marine n_INP_(*T*); (b) discuss what a compelling source of ice-nucleating particles is?

Model Answer:

1. It is apparent that the continental *n*_INP_ spectra below -15 °C (**Fig. 4**) are located above or overlapping with the upper bound of *n*_INP_ spectra from previous marine-predominant sites (**Fig. 2**). The highest *n*_INP_ observed in the continental site is ≈ 10^4^ L^-1^, which is about an order magnitude higher than the upper range of marine *n*_INP_, indicating that absolute INPs per unit volume at continental sites are much higher than previously investigated marine INP sources.
2. Yearly emission rates of soil dust are 1000 to 4000 teragrams, accounting for a compelling source of both total particle loading and INPs in the atmosphere. Thus, the continental dust might be responsible for the gap between continental *n*_INP_ (up to 10,000 L^-1^) and marine *n*_INP_ (< 1000 L^-1^). As inferred in **Figs. 2 and 4**, Above -15 °C, Atlantic *n*_INP_ exceeds continental *n*_INP_, suggesting marine-derived INPs can be highly active and as compelling as continental INPs at relatively high-temperature regions.

#### Question 11

Calculate the ice nucleus concentration in 5 µL suspension droplets at a frozen fraction of 0.5 at -20 °C.

Model Answer: $C_{INP}\left( T \right)= - \frac{\ln\left( 1-FF\left( T \right) \right)}{V_{d}} = - \frac{\ln\left( 1-0.5 \right)}{5 \times{10}^{-6}} =1.39 \times{10}^{5} L^{-1}$

#### Question 12

Calculate the upper/lower 95% binomial sampling confidence intervals of ice nucleus concentration in a total of 70 suspension droplets at a frozen fraction of 0.5 at -20 °C.

Model Answer:

Upper CI95% =(1/(1+((1.96^2)/70)))*(0.5 + ((1.96^2)/140)+(1.96*(SQRT((0.5*0.5/70)+((1.96^2)/4*70^2))))) = +127.96

Lower CI95% =(1/(1+((1.96^2)/70)))*(0.5 + ((1.96^2)/140)-(1.96*(SQRT((0.5*0.5/70)+((1.96^2)/4*70^2))))) = -126.96

#### Question 13

Calculate the homogeneously formed ice crystal concentration of 20 µm diameter droplet at t = 10 s. Assume the initial concentration of droplets (*n*_0_) of 100 cm^-3^. Presume the volume nucleation rate of 2.85 × 10^6^ cm^-3^ s^-1^.

Model Answer:

A volume of 20 µm diameter droplet is 4.19 x 10^-9^ cm^-3^ ($=\frac{4}{3} \pi{0.001}^{3}$). Using Eqn. 6, the *n*_ice_(*t*) value at t = 10 s is 11.25 cm^-3^ (= 100*(1-EXP((-2.85*10^6)* 4.19 x 10^-9^ *10)))

#### Question 14

For polydisperse droplets, show that you can calculate the volume nucleation rate from the measured ice formation rate.

Model Answer:

Eqn. 8 can be converted to:

$\Delta n_{ice}=\sum_{i} n_{i}V_{i}J_{v}\Delta t=v J_{v} \Delta t$ 🡪

in which, $\frac{\Delta n_{ice}}{\Delta t}$ represents the ice formation rate.

## **REFERENCES**

- Brooks, S. D., and Thornton, D. C. O.: Marine Aerosols and Clouds, Annual Review of Marine Science, 10, 289–313. doi:10.1146/annurev-marine-121916-063148, 2018.
- Cui, Z., Carslaw, K. S., Yin, Y., and Davies, S.: A numerical study of aerosol effects on the dynamics and microphysics of a deep convective cloud in a continental environment, J. Geophys. Res., 111, D05201, 2006.
- de Boer, G., Morrison, H., Shupe, M., and Hildner, R.: Evidence of liquid dependent ice nucleation in high‐latitude stratiform clouds from surface remote sensors, Geophysical Research Letters, 38, L01803, 2011.
- Duft, D. and Leisner, T.: Laboratory evidence for volume-dominated nucleation of ice in supercooled water microdroplets, Atmos. Chem. Phys., 4, 1997–2000, https://doi.org/10.5194/acp-4-1997-2004, 2004
- Field, P. R., Heymsfield, A. J., Shipway, B. J., DeMott, P. J., Pratt, K. A., Rogers, D. C., Stith, J., and Prather, K. A.: Ice in clouds experiment–layer clouds. Part II: Testing characteristics of heterogeneous ice formation in lee wave clouds, Journal of the Atmospheric Sciences, 69, 1066-1079, 2012.
- Hande, L. B. and Hoose, C.: Partitioning the primary ice formation modes in large eddy simulations of mixed-phase clouds, Atmos. Chem. Phys., 17, 14105–14118, 2017.
- Hiranuma, N., Auvermann, B. W., Belosi, F., Bush, J., Cory, K. M., Georgakopoulos, D. G., Höhler, K., Hou, Y., Lacher, L., Saathoff, H., Santachiara, G., Shen, X., Steinke, I., Ullrich, R., Umo, N. S., Vepuri, H. S. K., Vogel, F., and Möhler, O.: Laboratory and field studies of ice-nucleating particles from open-lot livestock facilities in Texas, Atmos. Chem. Phys., 21, 14215–14234, https://doi.org/10.5194/acp-21-14215-2021, 2021.
- Kanji, Z. A., Ladino, L. A., Wex, H., Boose, Y., Burkert-Kohn, M., Cziczo, D. J., and Krämer, M.: Overview of ice nucleating particles, Meteorological Monographs, 58, 1.1–1.33, 2017.
- Miller, A. J., Brennan, K. P., Mignani, C., Wieder, J., David, R. O., and Borduas-Dedekind, N.: Development of the drop Freezing Ice Nuclei Counter (FINC), intercomparison of droplet freezing techniques, and use of soluble lignin as an atmospheric ice nucleation standard, Atmos. Meas. Tech., 14, 3131–3151, https://doi.org/10.5194/amt-14-3131-2021, 2021.
- Möhler, O., Hiranuma, N., Höhler, K., Hoose, C., Hummel, M., Niemand, M., Oehm, C., Schmitt, T., Steinke, I., and Wagner, R.: Parameterizations of ice formation derived from AIDA cloud simulation experiments, in: Nucleation and Atmospheric Aerosols, 19th International Conference, edited by: DeMott, P. J. and O’Dowd, C. D., AIP Publishing, Melville, New York, 851-858, 2013.
- O'Sullivan, D., Murray, B. J., Malkin, T. L., Whale, T. F., Umo, N. S., Atkinson, J. D., Price, H. C., Baustian, K. J., Browse, J., and Webb, M. E.: Ice nucleation by fertile soil dusts: relative importance of mineral and biogenic components, Atmos. Chem. Phys., 14, 1853–1867, 2014.
- Seinfeld, J. H., Bretherton, C., Carslaw, K. S., Coe, H., DeMott, P. J., Dunlea, E. J., . . . Wood, R.: Improving our fundamental understanding of the role of aerosol-cloud interactions in the climate system, Proc Natl Acad Sci U S A, 113, 5781–5790. doi:10.1073/pnas.1514043113, 2016.
- Steinke, I., Hiranuma, N., Funk, R., Höhler, K., Tüllmann, N., Umo, N. S., Weidler, P. G., Möhler, O., and Leisner, T.: Complex plant-derived organic aerosol as ice-nucleating particles – more than the sums of their parts?, Atmos. Chem. Phys., 20, 11387–11397, 2020.
- Suski, K. J., Hill, T. C. J., Levin, E. J. T., Miller, A., DeMott, P. J., and Kreidenweis, S. M.: Agricultural harvesting emissions of ice-nucleating particles, Atmos. Chem. Phys., 18, 13755–13771, 2018.
- Tobo, Y., DeMott, P. J., Hill, T. C. J., Prenni, A. J., Swoboda-Colberg, N. G., Franc, G. D., and Kreidenweis, S. M.: Organic matter matters for ice nuclei of agricultural soil origin, Atmos. Chem. Phys., 14, 8521–8531, 2014.
- Tobo, Y.: An improved approach for measuring immersion freezing in large droplets over a wide temperature range, Sci. Rep., 6, 32930, <https://doi.org/10.1038/srep32930>, 2016.
- Phillips, V. T. J., Donner, L. J., and Garner, S. T.: Nucleation processes in deep convection simulated by a cloud-system-resolving model with double-moment bulk microphysics, Journal of the Atmospheric Sciences, 64, 738-761, 2007.
- Westbrook, C. D. and Illingworth, A. J.: Evidence that ice forms primarily in supercooled liquid clouds at temperatures > −27°C, Geophys. Res. Lett., 38, L14808, <https://doi.org/10.1029/2011GL048021>, 2011.
- Wilbourn, E. K. et al. (current manuscript): **Integrated Science Teaching in Atmospheric Ice Nucleation Research: Immersion Freezing Experiments, Submitted to Journal of Chemical Education, 2022.**

**Copyright Statement** Freezing of water droplets and ice-nucleating particles by Elise K. Wilbourn, Sarah Alrimaly, Holly Williams, Jacob Hurst, Gregory P. McGovern, Todd A. Anderson, and Naruki Hiranuma is marked with CC0 1.0 Universal Creative Commons license. To view a copy of this license, visit http://creativecommons.org/publicdomain/zero/1.0
