# Supplementary material for: Integrated Science Teaching in Atmospheric Ice Nucleation Research: Immersion Freezing Experiments
Source: J Chem Educ. 2023 Mar 8;100(4):1511–22. doi: 10.1021/acs.jchemed.2c01060 (PMC10100551; doi:10.1021/acs.jchemed.2c01060)
Supplement: Supplementary file 1 — ed2c01060_si_001.zip [file ed2c01060_si_001.zip › SI_Files/SI_Sect_S1_Module_3_SEM_EDX.docx]

**Supporting Information:**

**Integrated Science Teaching in Atmospheric Ice Nucleation Research:**

**Immersion Freezing Experiments**

Elise K. Wilbourn^1,♦^, Sarah Alrimaly^1,♦^, Holly Williams^1^, Jacob Hurst^2^, Gregory P. McGovern^2^,

Todd A. Anderson^3^, and Naruki Hiranuma^1,^*

^1^Dept. of Life, Earth, and Environmental Sciences, West Texas A&M University, Canyon, TX, 79016

^2^ Dept. of Chemistry and Physics, West Texas A&M University, Canyon, TX, 79016

^3^ Dept. of Environmental Toxicology, Texas Tech University, Lubbock, TX, 79416

^♦^These authors equally lead and contributed to this work

*Corresponding author ([nhiranuma@wtamu.edu](mailto:nhiranuma@wtamu.edu))

# **Title: Module 3 - Elemental composition analysis of water residual particles by SEM-EDX**

Estimated Completion Times: 400 min

Introduction 30 min

Experimentation 330 min

Assessment 40 min

Prerequisites: Chemistry I, Chemistry II [and Analytical Chemistry (optional)]. Pre-completion of the WT-CRAFT module will help enhance learning efficiency.

Target course level: an upper-level undergraduate or graduate-level course in Environmental Science/Chemistry.

## **INTRODUCTION**

### Introduction Summary

This section contains targeted background content to prepare you for performing the scanning electron microscopy and energy-dispersive X-ray spectroscopy exercises to identify possible catalysts of heterogeneous freezing in the water in this lesson.

### Learning Objectives

- Learn the basics of scanning electron microscopy and energy-dispersive X-ray spectroscopy.
- Describe how to compute the atomic percentage (Atomic %) of elements.
- Discuss complementary chemical analysis to characterize the organics in water samples.

### Hypothesis Formulation

Do you expect to see any relationship between residual particle size, shape, and composition? If so, describe your hypothesis.

### Test Your Knowledge

1. An electron microscope and the associated chemical speciation function have a wide variety of applications in many different disciplines. It is not limited to chemistry and the atmospheric science field. List and discuss three specific applications in, for example, biology, medical science, and art.
2. Modern scanning electron microscopes can resolve the size of the specimen to the [ ] diameter range.

- <1 µm
- <1 nm
- <1 Å

1. What are the atomic weights of sodium, potassium, and chloride? Provide numbers with two decimal digits.
2. What kind of chemical analytical techniques would be useful to characterize the particles in water samples?

- Chromatography
- Mass spectrometry
- Spectroscopy
- All of above

### Answer Key:

1. The students’ answers will vary. Some examples include:

- Forensic investigation. Numerous crime-scene micro-traces, including glass and paint fragments, tool marks, drugs, explosives, and gunshot residue (GSR) can be visually and chemically analyzed with SEM.
- Material science: Investigations into nanotubes and nanofibres, high-temperature superconductors, mesoporous architectures, and alloy strength, all rely heavily on the use of SEMs for research and investigation.
- Microbiology: SEM has been widely used in environmental microbiology to characterize the surface structure of biomaterials and to measure cell attachment and changes in the morphology of bacteria.
- Art: Micrographs produced by SEMs have been used to create digital artworks. High-resolution 3D images of various materials create a range of diverse landscapes, image subjects are both alien and familiar.
- Medical science: SEMs are used in medical science to compare blood and tissue samples in determining the cause of illness and measuring the effects of treatments on patients (while contributing to the design of new treatments).

1. <1 µm
2. Na: 22.99 g/mol K: 39.10 g/mol Cl: 35.45 g/mol
3. All of above

### Subsection 1: Scanning Electron Microscope

The Scanning Electron Microscope (SEM) was first developed in the 1930s, followed by commercialization in the 1960s. A summary of the initial SEM development history is shown in **Fig. 1**. Knoll, an inventor of SEM and a collaborator with Ruska for the development of transmission electron microscope (TEM) development, first utilized an electron beam with a diameter of 100 µm in a vacuum-sealed glass tube to obtain an SEM image in the early 1930s. von Ardenne later consolidated the idea of the modern electron optical system with an electron gun, a condenser lens, and an objective lens to produce a fine electron beam of 4 nm in diameter. In the 1940s-1950s, many developments were made to improve the quality of secondary electron detectors and images by applying an electronic amplifier to produce accelerating voltages of <20 keV. These efforts led to the first commercialization of the SEM in 1965. Nowadays, some SEMs are equipped with transmitted electron (TE) and backscattered electron (BSE) detectors. During image processing, the TE signal can be mixed with the BSE signal to obtain high-quality images of the particles and stable signals over a prolonged period of computer-controlled operation.


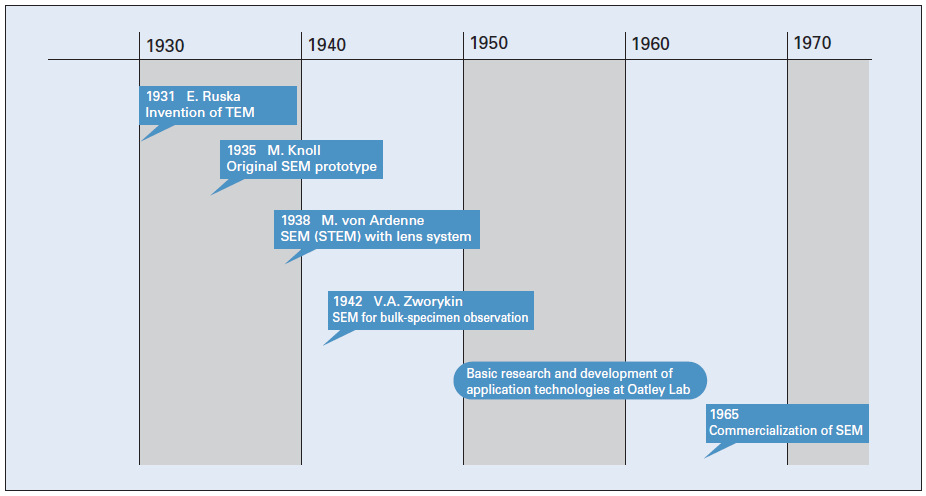


**Figure 1.** History of the SEM development at the early stage. Adapted from JEOL (Fig. 54, No.1101B972C(Ks)).

### Subsection 2: Principle of Energy Dispersive X-ray Spectroscopy

Modern yet affordable SEMs, such as JSM-6010LA (JOEL), allow users to obtain a microscopic image of individual submicron particles. By coupling with an Energy Dispersive X-ray spectroscopy function (EDX), the SEM-EDX combination enables speciation of particle elemental composition. In general, EDX quantifies the inelastic interaction between an electron beam and atoms in a particle. **Figure 2** illustrates the theory of this inelastic interaction. More specifically, when incoming electron radiation hits an atom, an inner shell electron of the atom (typically in the K-shell electron orbital) is removed, and then an outer electron fills the inner vacancy. Since outer shell electrons have a higher energy state, the excess energy per transfer of an electron is emitted as a photon. In this case, the proton has a wavelength in the X-ray region of the electromagnetic spectrum (0.01-10 nm). The weight percentage of each element is estimated by counting characteristic x-rays emitted by measured elements via a replacement of electrons. This inelastic interaction and associated photon energy/wavelength are very characteristic of each element, thus EDX can identify the elemental composition. Aerosol particle characterization using SEM-EDX has been widely applied to a variety of field-collected particles from both ground sites and research aircraft.


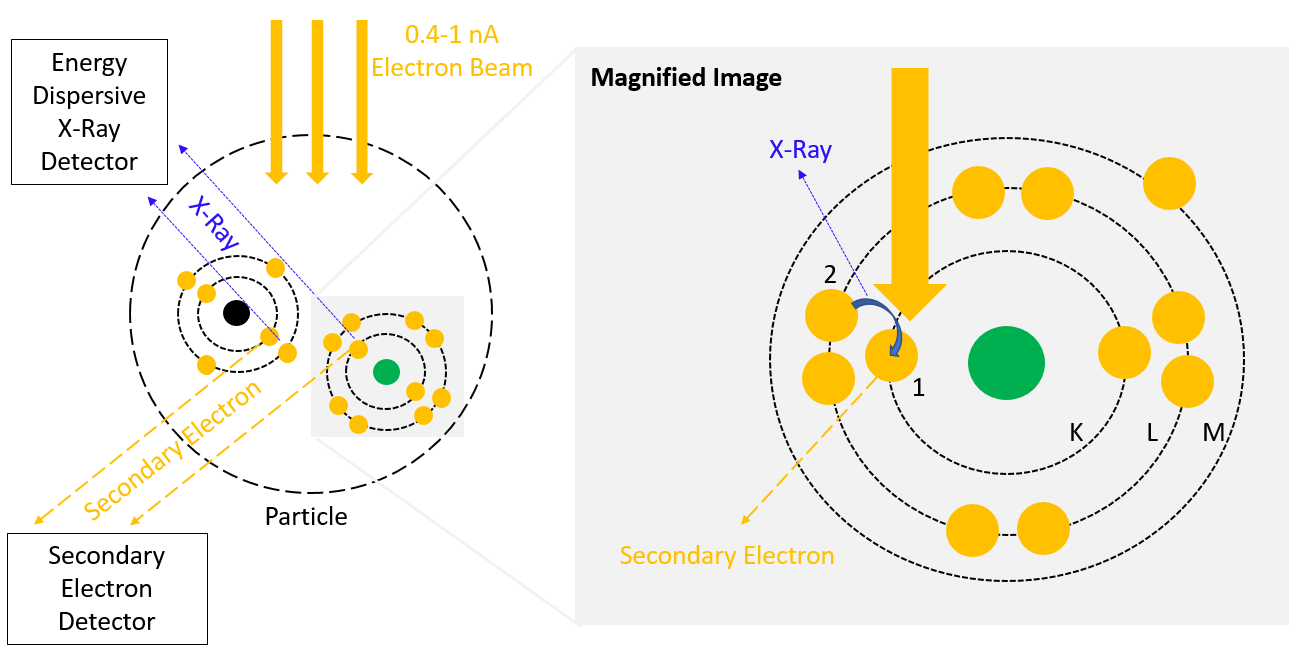


**Figure 2.** Concept of EDX’s inelastic interaction and interaction between the electron beam and emitted x-rays from the specimen. 1. An electron in the K-shell orbital is pushed out by the incoming electron beam, and 2. An electron in an outer shell replaces an inner one, emitting excess X-ray energy (Moon, 2010).

### Subsection 3: Description of Atomic Percentage (Atomic %)

One advantage of the EDX technique is that the measurements of weight percentage and Atomic % for specified elements can be obtained on a single particle basis. The Atomic % value represents the number of atoms of that element, at that weight percentage, divided by the total number of atoms in the sample. An initial step to acquiring Atomic % is to compute the atomic proportion, which is a ratio of each element's weight percentage (i.e., the weight of that element measured in the sample divided by the weight of all measured elements in the sample multiplied by 100) to its atomic weight [*atomic proportion = element weight % / atomic weight*]. By estimating this for all elements in the sample, it is possible to a list of atomic proportions. Then, summing these together results in obtaining a total atomic weight proportion [*TAWP = Σ atomic proportion*]. Finally, Atomic % can be calculated for each element in the sample by dividing its atomic proportion by TAWP [*Atomic % = atomic proportion / TAWP*].

Here is an example using sodium chloride. Let’s say EDX gives you:


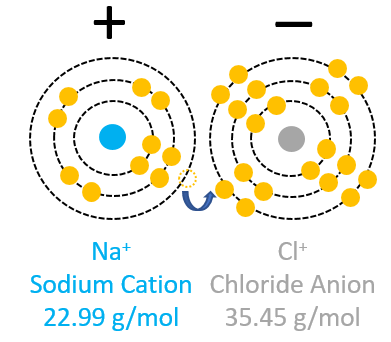


Na: element weight % = 60 %

Cl: element weight % = 40 %

You can divide each weight % by the atomic weights of Na (22.99 g/mol) and Cl (35.45 g/mol) to compute the atomic proportion:

Na atomic proportion: 60 / 22.99 = 2.61

Cl atomic proportion: 40 / 35.45 = 1.13

Then you divide each by a total atomic weight proportion (TAWP: 3.74 = 2.61 + 1.13) to find the atomic percentages:

**Figure 3.** Sodium chloride and ionic bonding between atoms. Transfer of one electron on the M shell of sodium to that of chloride atom forms sodium chloride.

Na Atomic %: 2.61 / 3.74 * 100 = 69.79 %

Cl Atomic %: 1.13 / 3.74 * 100 = 30.21 %

Thus, you have the atomic % for Na and Cl.

### Subsection 4: Complementary Chemical Analyses: What Can Act as an Ice-Nucleating Particle (INP) in Water?

Besides the SEM-EDX analysis, which will be conducted in this module, other complementary chemical analysis techniques can be applied to characterize the composition of potential INPs. Here you will see example applications of a gas chromatography system coupled with a mass selective detector (GC-MS) and a nuclear magnetic resonance spectroscopy (NMR) instrument to pre-assess possible INPs in the tap and HPLC water samples (description of each instrument is given below). Some impurities were identified in the instructor’s samples. In particular, a non-negligible amount of methyl/alkyl organic compounds were found in the tap water sample. Sub-sections below describe each analytical procedure and result.

#### 4.1. GC-MS results

**Figure 4** shows the GC-MS total ion chromatograms (retention time vs. MS signal) of a GC-MS extraction blank and water samples, as well as identified volatile organic compound/semi-volatile organic compound (VOC/SVOC) peaks. While the HPLC water spectrum shows no notable peaks other than the internal standard peak at a retention time (RT) of 20 min, the tap water sample appears to contain organics with partial matches in the National Institute of Standards and Technology (NIST) spectral library. For instance, the methyl group of alkyl organic compounds (trimethyl pentane at RT of 10 min) identified in the water samples may act as INPs in tap water. The chemical contaminants identified in the water samples may be native to the water or could come from contamination during the experiment or sampling. This emphasizes the importance of good laboratory technique, including minimizing the use of personal care products (colognes, perfumes, lotions) during collection, as these are a known source of the identified contaminants. It is noteworthy that the solid-phase extraction (SPE) cartridges used in this GC-MS measurement appear to contain impurities that could be mistakenly identified as coming from the experimental samples. This emphasizes the importance of a thorough activation step (in our case, sequentially hexane, acetone, and deionized water) in SPE procedures prior to the addition of samples.


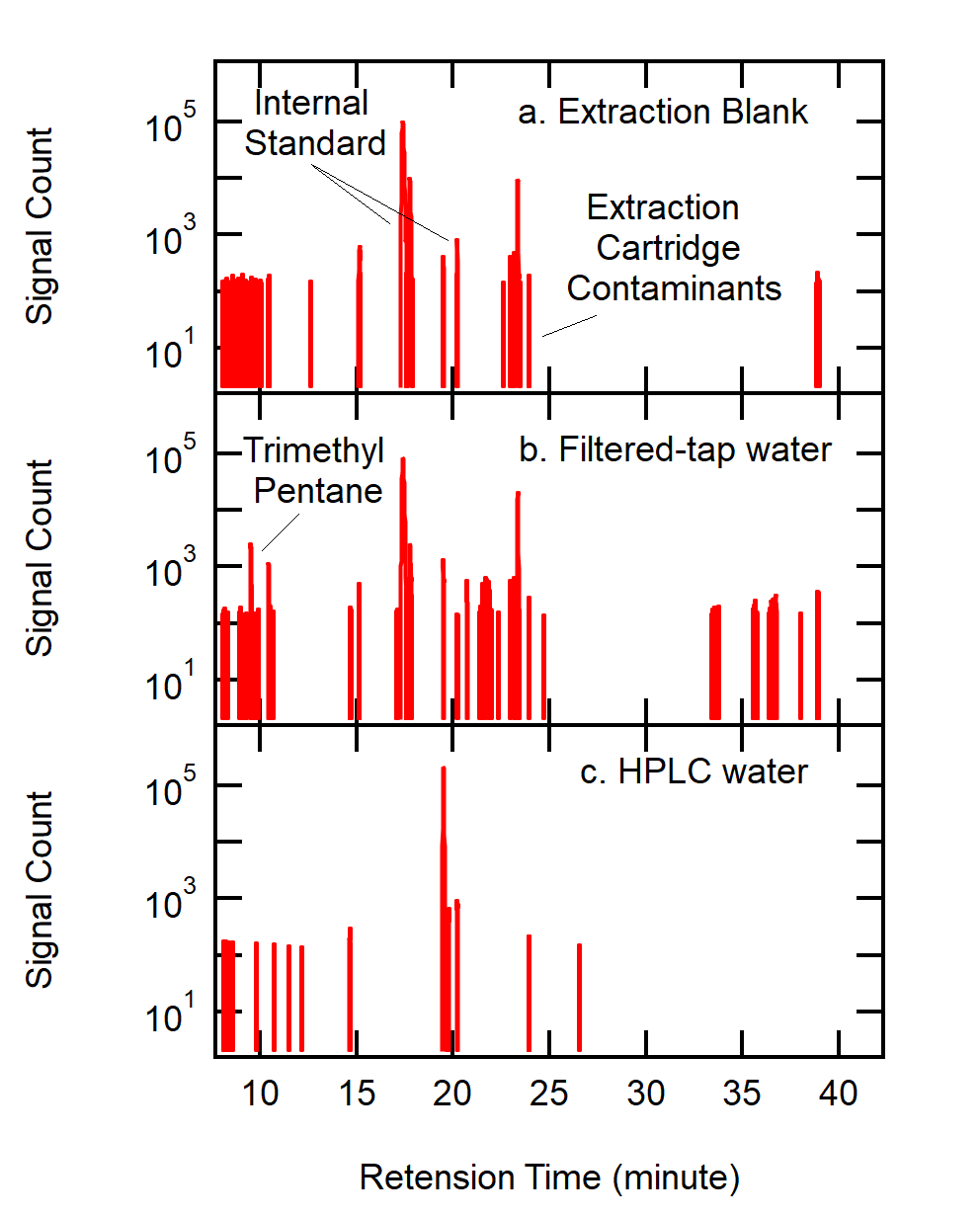


### ***Characterization methodology of VOC/SVOC by GC-MS:*** Vaporized water of each sample was individually assessed for VOC/SVOC by means of GC-MS. Water samples (250 mL) were processed using SPE cartridges (C_18_; 1000 mg/8-mL) after spiking with a surrogate/internal standard (desethyl atrazine or atrazine). SPE cartridges were eluted with 0.5 mL hexane (4X), filtered (0.2 µ PTFE syringe filters), and placed in auto sample vials. Quality control samples included blanks (hexane), extraction blanks (SPE cartridges eluted with hexane), and samples of H_2_O_2_ extracted by SPE. A GC-MS was used to screen water sample extracts for analytes of interest. More specifically, an Agilent 6890 series GC system was coupled with 5973 mass selective detector in the scan mode (m/z = 50 – 450 amu). A detailed description of the utilized system is addressed in Heintzman et al. (2015) and Chase et al. (2012), so only a short description is provided in this manuscript. Briefly, a capillary GC column (30 m x 0.25 mm; 0.25 µm film thickness) with He as the carrier gas was heated with a gentle temperature ramp and held at the final temperature (310° C) for an extended time. The goal of this GC-MS assessment was to assure elution of all potential contaminants in the extracted water. Chromatographic peaks in samples were searched against a NIST mass spectral library.

**Figure 4.** Volatile and semi-volatile organic compounds were identified by means of GC-MS. We show GC-MS spectra of extraction blank (a), filtered-tap water (b), and HPLC water (c).

#### 4.2. NMR results

**Figure 5** shows the NMR results. The chemical shift (x-axis) describes the chemical environment of the proton. Different functional groups appear in characteristic regions of the spectrum which helps in the identification of the protons generating the signal. Many different regions overlap or are wide, requiring additional methods to aid in chemical determination. The y-axis shows intensity, and by correlation, approximates the relative number of protons in that specific chemical environment. As shown in **Fig. 5a**, only two impurities were observed in the stock deuterated water (D_2_O); nondeuterated H_2_O / HDO and a very small broad peak that does not interfere with the analysis of the water samples. The presence of many common laboratory impurities, such as solvents, can be ruled out by comparing the spectrum with their known chemical shifts (Fulmer et al., 2010). The tap water sample showed a few peaks in the alkyl region (~3.5 ppm). These peaks may correspond to ketone [-CH_2_-OR (R= alkyl or hydrogen)] or an alkyl halide [-CH_2_-X  (X=halogen)], such as clionasterol (Silverstein and Bassler, 1962). It is possible that in water, which is a highly polar solvent, esters (like those present in stearate) may appear in this region of the NMR spectrum. The HPLC water sample did not show significant peaks in the same position (**Fig. 5c**). However, the sharp peak at ~3.2 ppm may also derive from ketone. It is worth noting that the exact identity of the contaminants in the water sample was not identified due to the lack of a library of relevant compounds analyzed using D_2_O. Since peak position is partially dependent on solvent, spectra can only be compared when the solvents are identical. This also hampered our ability to create our own library, due to the limited solubility of the relevant compounds in D_2_O. It is also worth noting that the pulse sequences were not set up for the quantification of potential contaminants for this study. Therefore, it is important to keep in mind that our NMR spectral peak results provide only qualitative information regarding contaminants.


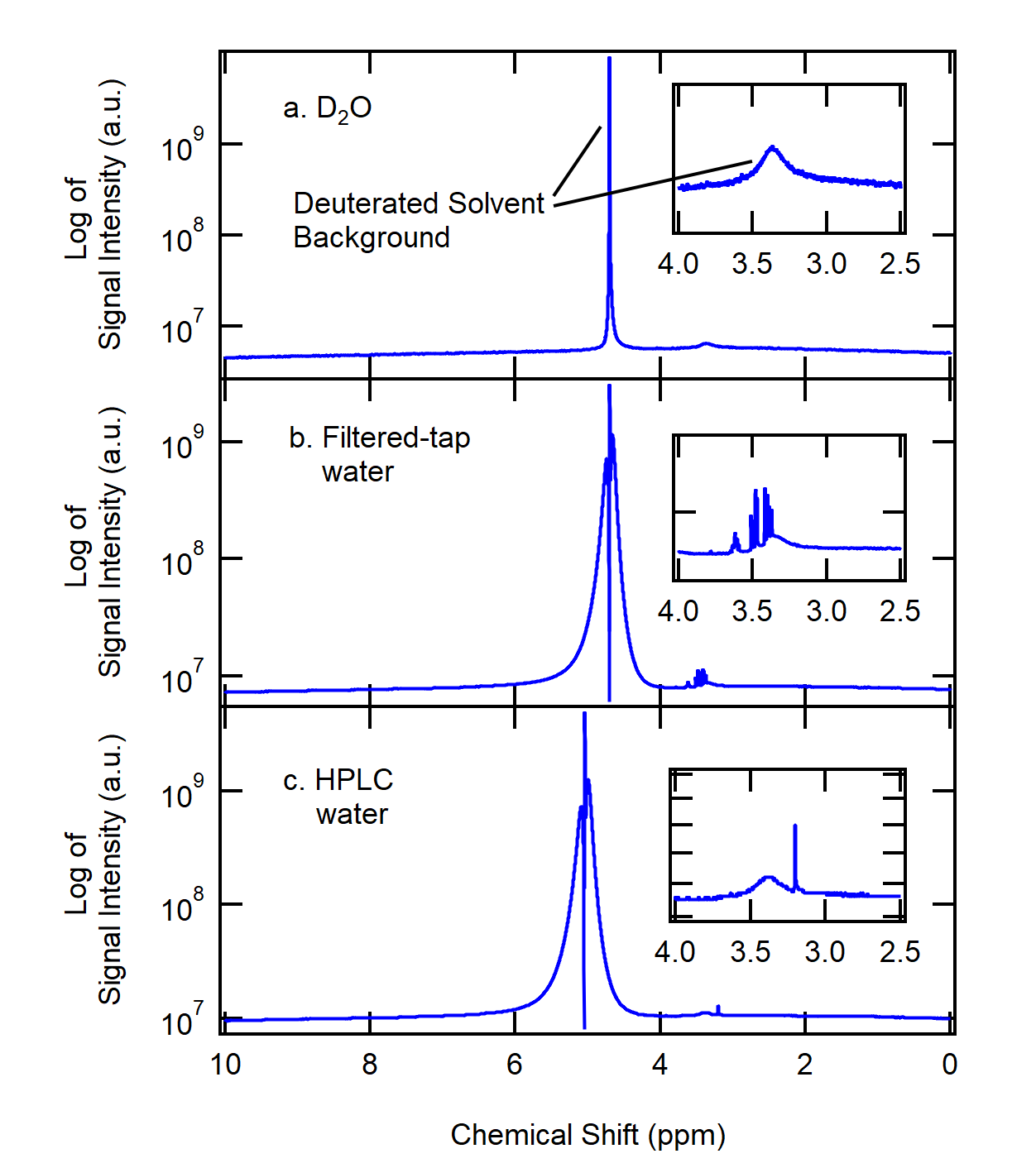


### *Method of identifying non-volatile organics by NMR*

NMR was used to determine the purity and molecular identity of insoluble organic matter in our water samples (Giraudeau et al., 2015). In this study, the water samples were dissolved in a deuterated solvent for analysis by NMR. D_2_O was chosen for our solvent because it is chemically identical to the bulk of the sample. Therefore, the sample completely dissolved in the solvent and did not interfere with the interpretation of the resulting spectra (Fulmer, 2010). Prior to sample analysis, the impurity content of neat deuterated water solvent was analyzed using a related experimental method as sample analysis. Approximately 0.75 mL of stock D_2_O was pipetted into a 5mm NMR tube. The sample was analyzed using a Bruker Avance III 400 MHz NMR spectrometer (Liu et al., 2017). The sample was analyzed for ^1^H using a zg30 proton detect pulse program for 8192 scans.

Water samples were prepared for analysis by using a Gilson micropipette to add 0.35 mL D_2_O and 0.40mL water sample into a 5 mm NMR tube. The sample was mixed then loaded into the same NMR spectrometer that was used to analyze neat D_2_O. Since NMR spectroscopy is an isotope-specific method of analysis, the amount of proteo water would swamp out the signal from the contaminants (Fulmer et al., 2010). To compensate for this, the sample was analyzed for ^1^H and then a water suppression pulse program, zgpr, was used to minimize the signal from H_2_O for 8192 scans.

**Figure 5.** NMR spectra of D_2_O (a), filtered-tap water (b), and HPLC water (c). Sub-panels are the magnified section of the characteristic chemical shift range (2.5-4.0 ppm) for each sample.

## **EXPERIMENTATION**

### Experimentation Summary

This section will guide you through competency-building exercises. You will be challenged with applying the knowledge you gained in the introduction section to complete the activities successfully.

### Learning Objectives

- Analyze two contrasting water samples (tap vs. pure) for their residual particle compositions.
- Relate water quality to observed contamination.

### Materials

Read through the procedures listed in the exercises on the next pages before beginning. Then, gather all of the materials listed below and begin Exercise 1.

| **Qty.** | **Item** | **Manufacturer, Model, Estimate ~$12,800** |
| --- | --- | --- |
| 1 | SEM-EDX system* | JOEL, JSM-6010LA, $12,000 |
| 1 | SEM Filament, K Type | JEOL, 417004, $37.50 ($450 for 12) |
| 1 | 9.5 x 9.5mm diameter cylinder-style SEM specimen stub, aluminium | JEOL, 10-005110-50, $20 |
| 1 | Multiple stub holder | JEOL, 12-000515, $35 |
| 1 | SEM stage adaptor | EM-Tec, JV50 11-000554, $150 |
| 1 | Carbon conductive tape, 9 mm diameter | TED PELLA, INC., PELCO Tabs 16084-3, $14.50 |
| 1 | Sterile Syringe Filter | VWR, 28145-477, $75 |
| 1 | 50 mL Luer-Lok Syringe | VWR, 309653, $95 for 40 |
| 1 | Tweezer/forceps | VWR, 82027-386, $30 |
| 1 | Dissecting scissors | VWR, 82027-578, $25 |
| 1 | Hotplate | Bipee, SH-3, $275 |
| 1 | ImageJ software** | National Institutes of Health, Free |
| 2 | Sterile polypropylene tube with cap <15 mL volume | e.g., VWR, 10026-076, *$1.20* ($200 for 500 tubes) |

*no autonomous particle detection function and motored-stage are required. The system should include the inset analytical software, such as InTouchScope JSM-6010PLUS (Ver. 3.01); **Install this in the SEM system computer.

The following materials might be available at your laboratory or the university physical plant.

| **Qty.** | **Item** |
| --- | --- |
| 1 | Aluminum foil |
| 1 | Tap water (<15 mL) |
| 1 | Distilled water |
| 1 | Ultrapure water (<15 mL 18.2 MΩ•cm MilliQ water or HPLC grade water, Sigma Aldrich, 270733) |
| 3 | Pair of gloves |
| 1 | Isopropyl alcohol |
| 1 | Kimwipes |
| 1 | Aluminum tape |
| 2 | 10 mL disposable pipette |

### Safety

- Safety is of the utmost importance in the lab. You must always act responsibly and be able to identify potential dangers and take the proper precautions in the event of an accident. In the lab, the most important tools you have to avoid potential issues are your brain and the ability to plan out the future. After previewing Exercise 1, consider what safety precautions you need to conduct a safe experiment and how to put those precautions into action.
- Be sure that the SEM-EDX system, which is a radiation-generating device, is well-shielded. Radiation-generating devices should be regularly inventoried by the health and safety office. It is important that the integrity of the shielding is maintained, that all existing interlocks are functioning, and that you are aware of radiation safety considerations and emergency contact information.
- You should be trained for safely operating SEM or conducting SEM measurements with the attendance of your instructor.
- Safety Data Sheets (SDSs) will be provided for all chemicals used in this module. SDSs provide information about physical properties, health risks, fire explosion data, and other important information associated with these chemicals. Before handling or using a chemical, you should refer to the SDS for that chemical.
- It is your responsibility to inform the instructor in writing of any health conditions that may prevent you from safely using a chemical (pregnancy, autoimmune deficiency, etc.). It is also the responsibility of the student to report any spill or problems found while storing or using a chemical. If you are unsure about a chemical, always ask. If you see any unsafe conditions, notify your instructor immediately. If you are unsure about the proper and safe operation of any piece of equipment, ask your instructor for proper instructions. All injuries, spill of materials, and unsafe conditions must be reported to the instructor immediately.
- Any pregnant students, or students planning to become pregnant, should consult their health care provider to determine what, if any, additional precautions are needed based on their individual situation. While the university cannot mandate that the student notify that they are pregnant or are planning to become pregnant, the university strongly recommends that students provide notification so appropriate steps can be taken to ensure the health of both parent and child.
- Eyesight is one of our most important senses in science and should be protected at all times from potential chemical spills and splashes, flying objects and dust, and specific light spectrums. It is important to always wear safety goggles when participating in experiments.
- Food, drinks, and smoking are never allowed in the lab area.
- Always be sure to protect your body from potential harm by keeping your hair tied back, wearing clothing that covers your exposed skin, and by wearing closed-toe shoes.
- Always be sure to protect your work areas from damage caused by experimentation. This means covering your surface in paper towels or plastic when necessary.
- Experiments may also require physical activity so be sure to consider these hazards.
- Be sure to contact your physician first or ask a partner for help in cases that require rigorous physical activity.
- If you need to climb while taking measurements, be sure to use a sturdy stool, chair, or ladder and take the necessary precautions to prevent falling. If you need to climb it is wise to have a partner with you to stabilize what you are climbing. If you are working with moving equipment act cautiously to ensure that the equipment doesn’t lose control and cause injury.
- Almost all chemicals found in a laboratory can be toxic to the human body. To be certain that there is no accidental consumption be sure to never taste, eat, or drink anything in the lab. All labs should be thoroughly cleaned after experimentation to prevent accidental consumption. In the event of chemical ingestion, contact the National Poison Control Center and talk to a physician
- Be certain to wrap all non-chemical experimental items in paper towels or newspapers and throw them into the garbage. Be certain that the container is secured and inaccessible to children and animals.
- If you wear eyeglasses, it is still important to wear goggles over the top of the eyeglasses. This will protect your eyes from chemical spills, shattered glass, and flying objects. Safety goggles must always be worn when conducting experiments.
- Chemical spill protection checklist:
  - Long-sleeved shirts
  - Full-length pants
  - Closed-toe Shoes
  - Protected nitrile gloves
  - Face masks if directed
- Always be sure to pull back or pin down hair to avoid sources of flames, chemicals, or other lab components.
- In the event of an accident always know the location of first aid kits and make sure that they are stocked and easily accessible.
- All laboratories should also have access to eyewash stations that look like water fountains with two upward water faucets that look like they match the distance between eyes. In the event of an accident, the victim's head should be placed close to the fountain. The victim’s eyes should then be held open while the faucets are turned on to spray water into their eyes and clean the chemicals out of their eyes. In non-traditional labs like kitchens, you can also wash out your eyes with a sink faucet or hand-held shower wand. After using an eyewash be sure to contact a physician after washing your eyes.
- A safety shower is also used in all laboratories to put out fires or remove chemicals from the body.

## **Exercise 1 - Testing Water Quality**

In this exercise, you will examine two water samples (i.e., tap and ultrapure water) to characterize their residual particle elemental composition on a particle-by-particle basis.

###
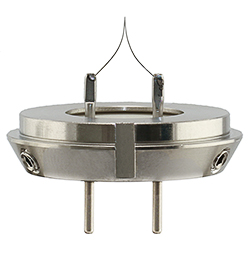
SEM-EDX Setup – be sure that your instructor is present to support you (15 min)

1. Ventilate the SEM chamber.
2. Inspect if the filament (**Fig. 6**) in the electron gun (**Fig. 7**) is in good condition (i.e., not burned out or worn off) and placed correctly. If necessary, go through the automatic gun control process via the InTouchScope JSM-6010PLUS software to optimize the gun axis alignment to control brightness changes.
3. Insert a blank aluminum stub half-covered with a thin copper plate in a specimen stage.
4. Via the InTouchScope JSM-6010PLUS software, put the SEM system under vacuum condition (~10^-4^ Pa). Note: While decreasing pressure in the specimen chamber, residual gas molecules are ionized by electrons and reach the specimen as positive ions to neutralize the charging.

**Figure 6.** JEOL K-Type Base Tungsten Filaments with Metal Ring.

1. After you confirm the chamber is under vacuum conditions, be sure to check with your instructor to confirm the aperture between the condenser lens (narrowing the electron probe) and the objective lens is placed correctly and its angle is optimized to adequately pass the electron beam through the center of the objective lens (**Fig. 7**). The aperture must be placed exactly on the optical axis of the electron beam.
2. Go through the electron beam energy calibration process to optimize the measurement conditions for EDX. The peak positions of the EDX spectrometer should be calibrated. This allows users to find the positions of characteristic X-ray lines within their systematic error range. A miscalibrated system will shift X-ray peaks to improper energies, increasing the risk of misidentification of elements. Because of the possible drift of the electronics of the EDX systems, the calibration should be carried out monthly to ensure the quality of the EDS measurements. Calibration can usually be done on pairs of two widely separated X-ray lines, for example:

i) Overlapping Al Kα lines at ~1.486 keV (namely, with Kα2 at 1.486 keV and Kα1 at 1.487 keV) and overlapping Cu Kα lines at ~ 8.037 keV (namely, with Kα2 at 8.027 keV and Kα1 at 8.047 keV).

ii) Overlapping Cu Lα2,1 at 0.928 keV and overlapping Cu Kα lines at ~ 8.037 keV.

iii) Overlapping Cu Kα lines at ~ 8.037 keV and the zero-strobe peak.

The calibration may require increasing the accelerating voltage and spot size of the electron beam (to 20 keV and spot size 65), and thus the higher energy X-rays can be generated and recorded. Be sure to conduct the energy calibration with the condition of electron beam counts of 10,000 - 40,000 cps. Using the magnification of ~x500 is recommended to detect reasonable electron counts.

1. Ventilate the SEM chamber and remove the aluminum-copper sample stub from the specimen stage. **NOTE: SEM should be kept under vacuum conditions while it is not in use.**


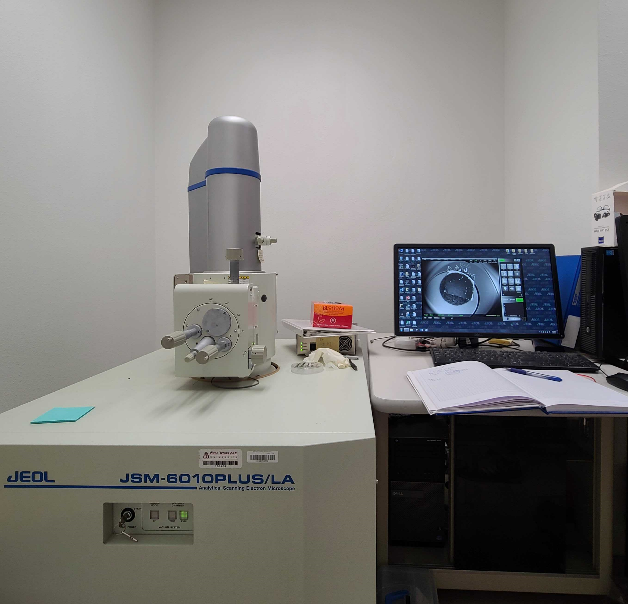

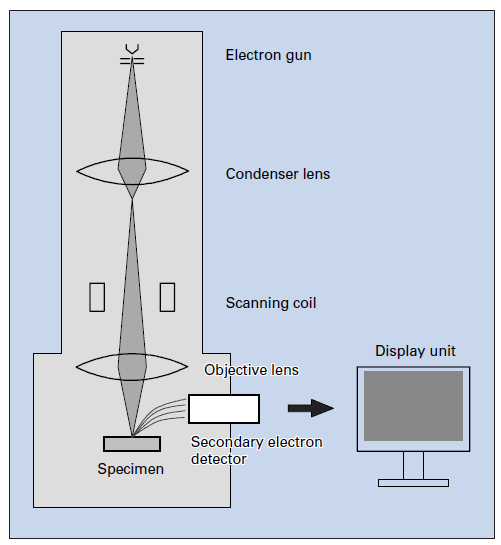


**Figure 7.** Image and basic construction of an SEM system. The schematic is adapted from JEOL (Fig. 54, No.1101B972C(Ks)).

### Sample Preparation (15 min)

Two water samples will be examined in this exercise: Ultrapure water and tap water. Be sure to stock at least 15 mL of each water sample by following the steps below.

1. Put on your gloves.
2. Use the permanent marker to label one 15 mL polypropylene tube Tap Water and a second tube Ultrapure Water.
3. Write your name and date on **Data Table 1**.
4. Fill the Tap Water tube with cold water from the faucet.

NOTE: You can alternatively prepare the filtered tap water sample by filtering the tap water through a sterile syringe connected to a sterile 25 mm diameter polycarbonate filter with 0.2 µm pore size (VWR, 28145-477 and 309653). Please prepare 15 mL of filtered tap water.

1. Record the source, well or municipal, and the area where collected as the description in **Data Table 1**.
2. Gather filtered and ultrapure water, fill the tube, and record the brand and area where manufactured as the description in **Data Table 1**.
3. You will add more information in the experiment/observation section later on, so leave this row blank for now.

**Note: Test all samples as soon as possible after they have been gathered. All remaining samples can be used for other complementary analyses if needed.**

| Your Name: __________________  Date: _______________________  **Data Table 1: Source of Water Samples** | |  |
| --- | --- | --- |
|  | Tap Water | Ultrapure Water |
| Source |  |  |
| Description |  |  |
| Experiment/Observation |  |  |

### Part 1: Tap Water Freezing Experiment (150 min)

1. Grab your **tap water sample** tube.
2. Put on your gloves.
3. Pre-clean a 15 cm x 20 cm aluminum foil sheet with 70% reagent alcohol and Kimwipes.
4. Create a roughly 5 cm x 5 cm x 5 cm aluminum cube using aluminum foil, and punch some holes on the side as shown in **Fig. 8**. If necessary, reinforce the cube with aluminum tape.


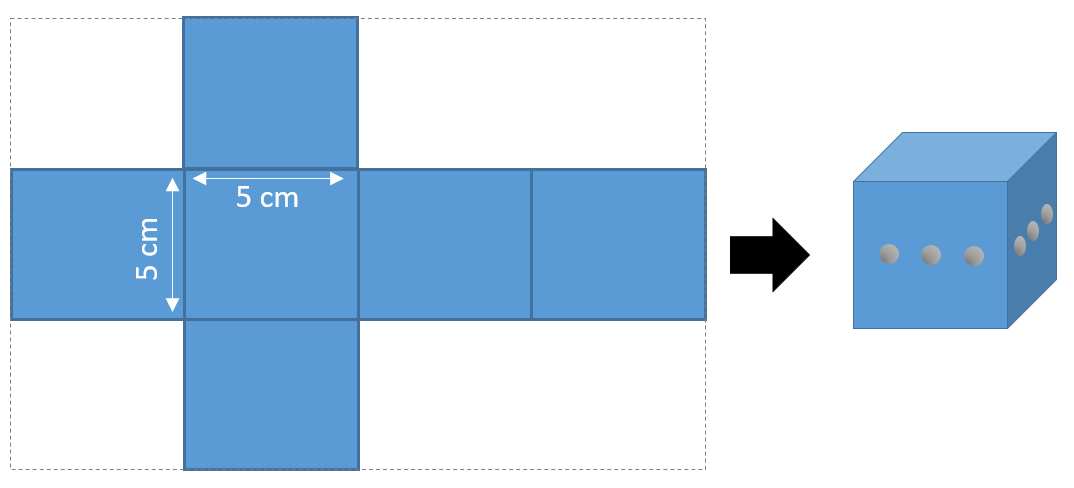


**Figure 8.** Aluminum cube as a water sample container.

1. Pipette 10 mL of water sample into the aluminum container made in Step (4) through a hole.
2. Place the aluminum container on a hotplate and evaporate all water.
3. After evaporating water and cooling the aluminum cube down, cut an 8 mm x 8 mm piece of the container bottom where you see extracted non-volatile residuals.
4. Place the cut aluminum piece from Step (7) on an SEM stub (9.5 mm) using carbon tape as seen in **Fig. 9**.
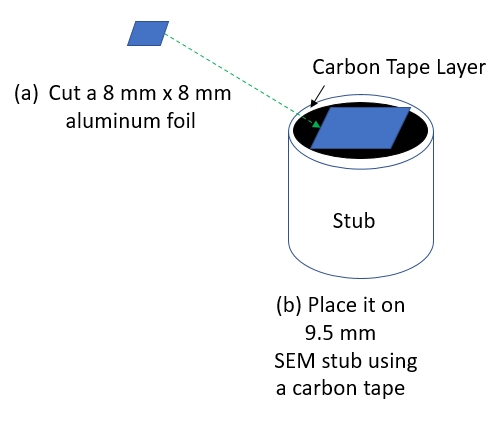

5. Ventilate the SEM chamber.
6. Once the chamber is ventilated, open the chamber door and place an SEM stub containing your water residuals on an SEM specimen stage.
7. Via the InTouchScope JSM-6010PLUS software, put the SEM system under vacuum condition (~10^-4^ Pa).
8. You will investigate 4 quadrat regions on an 8 mm x 8 mm specimen (i.e., Quad 1-4 in **Fig. 10**). During the measurement, a large area of multiple quadrats can be analyzed on each substrate. A large area is ideal because the spatial distribution of residual particles is typically inhomogeneous on the substrate and SEM/EDX requires analysis across a wide range of deposition spots. You will first use low microscopic magnification (~x10-30) to find an intra-quad area (roughly the center of the quadrat) in Quad 1. During microscopy operation, a working distance of 10 mm, an electron accelerating voltage of 20 keV with a beam current of 0.4-1 nA, and an irradiation time of 60 seconds can be used. Adjust focus, brightness, contrast, and stigmation of the image through InTouchScope JSM-6010PLUS as needed.

**Figure 9.** Sample stub setup.


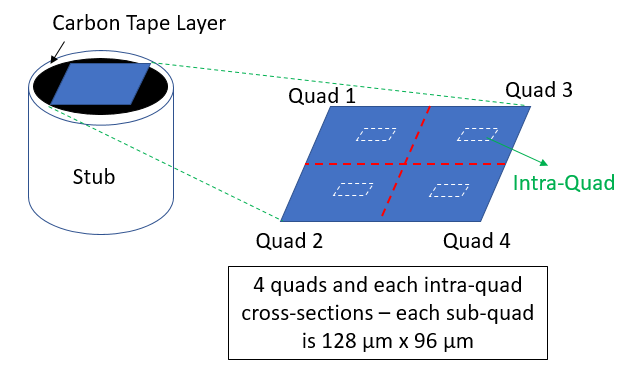
NOTE: the conditions may be different depending on the specimen. Increasing the accelerating voltage and spot size of the electron beam may be required, and thus higher energy X-rays can be generated and recorded.

1. Using the optimized conditions from Step 12, magnify the SEM image to ~ x1000 to capture the 128 µm x 96 µm area of one quadrat (e.g., Quad 1) on the specimen.

**Figure 10.** Quadrat assignment on the substrate.

1. Once you find an intra-quad, you will fine-tune focus, brightness, contrast, and stigmation of the image through InTouchScope JSM-6010PLUS. Be sure to record any notable observations in **Data Table 1**.
2. Using ImageJ, capture an overview image of at least 5 residual particles. Be sure to set the right scale by converting distance in pixels to the designated length unit (e.g., µm). The 8-bit grayscale images can be assessed on the ImageJ software to determine the size of captured residual particles by varying the minimum threshold gray value of 155-175 at the fixed maximum threshold value of 255 (**Fig. 11**).


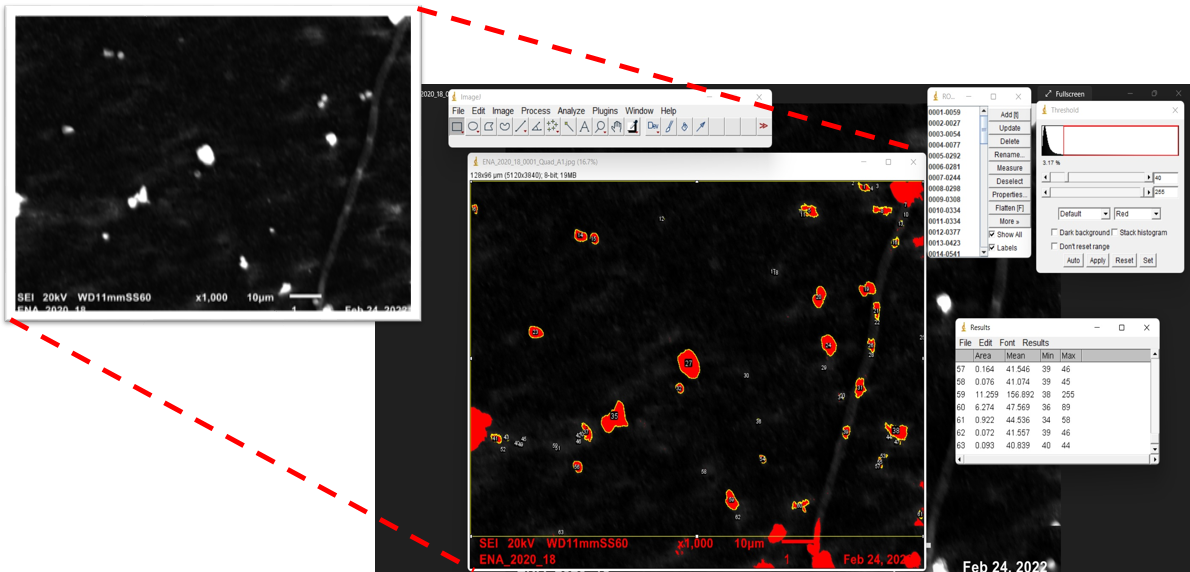


**Figure 11.** Snapshot example of ImageJ semi-automated particle detection and

size distribution analysis via the inset “analyze particles” function.

1. Using ImageJ, measure horizontal/vertical cross-section diameters and compute a cross-section average diameter of each residual particle as seen in **Fig. 12**. Be sure to select the residual particles in the size range of 0.3 to 10 µm in diameter. You can use the magnification of ~x10,000. Fill in sizes in **Data Table 2**. Also, record the detection rate of secondary scattered electron counts (cps, counts per second) in **Data Table 2**. This record will be useful to assess the condition of the electron filament and the timing of replacing it.


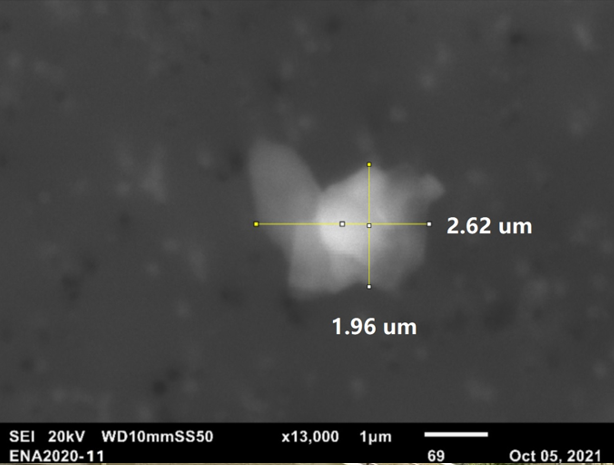


**Figure 12.** SEM single particle detection and size characterization.

1. Zoom in on a single particle and do EDX. Both X-ray spectra and images from the particles are recorded via computer software, such as InTouchScope JSM-6010PLUS (Ver. 3.01), which automatically enables tracking spectra on a particle-by-particle basis. On average, a student should be able to manually analyze 5 particles per 30 min. NOTE: To minimize background noise, high magnifications can be employed. Specifically, 3x10^5^ times for the particles that have diameters of smaller than 2.5 µm and 1.5 x10^5^ times for the particles that are larger than 2.5 µm in diameter are recommended. You will investigate the atomic percentage (Atomic %) abundance of 14 elements that can be found in natural water; C, N, O, Na, Mg, Si, P, S, Cl, K, Ca, Mn, Fe, and Zn. You will exclude the background signal of aluminum from the substrate, which was used for SEM-EDX.
   1. Save EDX spectrum (see the main manuscript Fig. 8).
   2. The inset quantitative analysis tool of InTouchScope JSM-6010PLUS will automatically compute weight % and atomic % of specified elements.
   3. Record ‘atomic %’ of 14 elements in **Data Table 2**.
2. Repeat Steps 13-17 for Quad 2.
3. Repeat Steps 13-17 for Quad 3.
4. Repeat Steps 13-17 for Quad 4.

### Part 2: Ultrapure Water Composition Characterization (90 min)

Grab your **ultrapure water sample** tube, and repeat Steps 2-20 for it. You probably will not be able to identify 20 residual particles. If so put N/A for any missing data in **Data Table 2**.

**Data Table 2.** Summary data of SEM-EDX.

Tap Water

| Quad | Particle ID |  | Cross-section size (µm) | | |  | Electron counts |  | Atomic % | | | | | | | | | | | | | |
| --- | --- | --- | --- | --- | --- | --- | --- | --- | --- | --- | --- | --- | --- | --- | --- | --- | --- | --- | --- | --- | --- | --- |
|  |  |  | Horizontal | Vertical | Average |  | (cps) |  | C | N | O | Na | Mg | Si | P | S | Cl | K | Ca | Mn | Fe | Zn |
| 1 | 1 |  |  |  |  |  |  |  |  |  |  |  |  |  |  |  |  |  |  |  |  |  |
|  | 2 |  |  |  |  |  |  |  |  |  |  |  |  |  |  |  |  |  |  |  |  |  |
|  | 3 |  |  |  |  |  |  |  |  |  |  |  |  |  |  |  |  |  |  |  |  |  |
|  | 4 |  |  |  |  |  |  |  |  |  |  |  |  |  |  |  |  |  |  |  |  |  |
|  | 5 |  |  |  |  |  |  |  |  |  |  |  |  |  |  |  |  |  |  |  |  |  |
| 2 | 6 |  |  |  |  |  |  |  |  |  |  |  |  |  |  |  |  |  |  |  |  |  |
|  | 7 |  |  |  |  |  |  |  |  |  |  |  |  |  |  |  |  |  |  |  |  |  |
|  | 8 |  |  |  |  |  |  |  |  |  |  |  |  |  |  |  |  |  |  |  |  |  |
|  | 9 |  |  |  |  |  |  |  |  |  |  |  |  |  |  |  |  |  |  |  |  |  |
|  | 10 |  |  |  |  |  |  |  |  |  |  |  |  |  |  |  |  |  |  |  |  |  |
| 3 | 11 |  |  |  |  |  |  |  |  |  |  |  |  |  |  |  |  |  |  |  |  |  |
|  | 12 |  |  |  |  |  |  |  |  |  |  |  |  |  |  |  |  |  |  |  |  |  |
|  | 13 |  |  |  |  |  |  |  |  |  |  |  |  |  |  |  |  |  |  |  |  |  |
|  | 14 |  |  |  |  |  |  |  |  |  |  |  |  |  |  |  |  |  |  |  |  |  |
|  | 15 |  |  |  |  |  |  |  |  |  |  |  |  |  |  |  |  |  |  |  |  |  |
| 4 | 16 |  |  |  |  |  |  |  |  |  |  |  |  |  |  |  |  |  |  |  |  |  |
|  | 17 |  |  |  |  |  |  |  |  |  |  |  |  |  |  |  |  |  |  |  |  |  |
|  | 18 |  |  |  |  |  |  |  |  |  |  |  |  |  |  |  |  |  |  |  |  |  |
|  | 19 |  |  |  |  |  |  |  |  |  |  |  |  |  |  |  |  |  |  |  |  |  |
|  | 20 |  |  |  |  |  |  |  |  |  |  |  |  |  |  |  |  |  |  |  |  |  |

Ultrapure Water

| Quad | Particle ID |  | Cross-section size (µm) | | |  | Electron counts |  | Atomic % | | | | | | | | | | | | | |
| --- | --- | --- | --- | --- | --- | --- | --- | --- | --- | --- | --- | --- | --- | --- | --- | --- | --- | --- | --- | --- | --- | --- |
|  |  |  | Horizontal | Vertical | Average |  | (cps) |  | C | N | O | Na | Mg | Si | P | S | Cl | K | Ca | Mn | Fe | Zn |
| 1 | 1 |  |  |  |  |  |  |  |  |  |  |  |  |  |  |  |  |  |  |  |  |  |
|  | 2 |  |  |  |  |  |  |  |  |  |  |  |  |  |  |  |  |  |  |  |  |  |
|  | 3 |  |  |  |  |  |  |  |  |  |  |  |  |  |  |  |  |  |  |  |  |  |
|  | 4 |  |  |  |  |  |  |  |  |  |  |  |  |  |  |  |  |  |  |  |  |  |
|  | 5 |  |  |  |  |  |  |  |  |  |  |  |  |  |  |  |  |  |  |  |  |  |
| 2 | 6 |  |  |  |  |  |  |  |  |  |  |  |  |  |  |  |  |  |  |  |  |  |
|  | 7 |  |  |  |  |  |  |  |  |  |  |  |  |  |  |  |  |  |  |  |  |  |
|  | 8 |  |  |  |  |  |  |  |  |  |  |  |  |  |  |  |  |  |  |  |  |  |
|  | 9 |  |  |  |  |  |  |  |  |  |  |  |  |  |  |  |  |  |  |  |  |  |
|  | 10 |  |  |  |  |  |  |  |  |  |  |  |  |  |  |  |  |  |  |  |  |  |
| 3 | 11 |  |  |  |  |  |  |  |  |  |  |  |  |  |  |  |  |  |  |  |  |  |
|  | 12 |  |  |  |  |  |  |  |  |  |  |  |  |  |  |  |  |  |  |  |  |  |
|  | 13 |  |  |  |  |  |  |  |  |  |  |  |  |  |  |  |  |  |  |  |  |  |
|  | 14 |  |  |  |  |  |  |  |  |  |  |  |  |  |  |  |  |  |  |  |  |  |
|  | 15 |  |  |  |  |  |  |  |  |  |  |  |  |  |  |  |  |  |  |  |  |  |
| 4 | 16 |  |  |  |  |  |  |  |  |  |  |  |  |  |  |  |  |  |  |  |  |  |
|  | 17 |  |  |  |  |  |  |  |  |  |  |  |  |  |  |  |  |  |  |  |  |  |
|  | 18 |  |  |  |  |  |  |  |  |  |  |  |  |  |  |  |  |  |  |  |  |  |
|  | 19 |  |  |  |  |  |  |  |  |  |  |  |  |  |  |  |  |  |  |  |  |  |
|  | 20 |  |  |  |  |  |  |  |  |  |  |  |  |  |  |  |  |  |  |  |  |  |

## **Exercise 2 – Data Analysis of Residual Particle Composition of Water**

### Part 1: Tap Water Composition Data Analysis (30 min)

- 1. Using Eqn. [1], calculate the standard deviation (i.e., variation or dispersion of a set of values) for the size and each element, and record the numbers in **Data Table 3**.

$\sigma= \sqrt{\frac{\sum_{i} \left( x_{i}-\mu\right)^{2}}{n-1}}$ [1]

- $\sigma$ = standard deviation
- *x_i_* = atomic % of individual element
- *µ* = mean
- *n* = number of observation (= residual particles analyzed)
- *n* – 1 = # of degrees of freedom = number of independent values on which a result is based (n > 2)
  1. Using Eqn. [2], calculate the standard error (i.e., the standard deviation of its sampling distribution) for the size and each element, and record the numbers in **Data Table 3**.

$\varepsilon=\frac{\sigma}{\sqrt{n}}$ [2]

- $\varepsilon$ = standard error
  1. Using Eqn. [3], calculate the 95% confidence interval (CI95%; i.e., the range of values within which there is a specified probability, 95%, that the true value will occur) for the size and each element, and record the numbers in **Data Table 3**.

$CI95\%=\frac{t(\alpha_{0.05})\sigma}{\sqrt{n}}$ [3]

- *t* = student t value at significance level (α) of 5% (constant - table)

### Part 2: Ultrapure Water Composition Data Analysis (30 min)

Repeat 1-3 using your ultrapure water data.

**Data Table 3.** Analysis of SEM-EDX data.

| Water Type | Parameters |  | Cross-section size |  | Atomic % | | | | | | | | | | | | | |
| --- | --- | --- | --- | --- | --- | --- | --- | --- | --- | --- | --- | --- | --- | --- | --- | --- | --- | --- |
|  |  |  | Average (µm) |  | C | N | O | Na | Mg | Si | P | S | Cl | K | Ca | Mn | Fe | Zn |
| Tap | *µ* |  |  |  |  |  |  |  |  |  |  |  |  |  |  |  |  |  |
|  | *n* |  |  |  |  |  |  |  |  |  |  |  |  |  |  |  |  |  |
|  | *σ* |  |  |  |  |  |  |  |  |  |  |  |  |  |  |  |  |  |
|  | *ε* |  |  |  |  |  |  |  |  |  |  |  |  |  |  |  |  |  |
|  | *t* |  |  |  |  |  |  |  |  |  |  |  |  |  |  |  |  |  |
|  | CI95% |  |  |  |  |  |  |  |  |  |  |  |  |  |  |  |  |  |
| Ultrapure | *µ* |  |  |  |  |  |  |  |  |  |  |  |  |  |  |  |  |  |
|  | *n* |  |  |  |  |  |  |  |  |  |  |  |  |  |  |  |  |  |
|  | *σ* |  |  |  |  |  |  |  |  |  |  |  |  |  |  |  |  |  |
|  | *ε* |  |  |  |  |  |  |  |  |  |  |  |  |  |  |  |  |  |
|  | *t* |  |  |  |  |  |  |  |  |  |  |  |  |  |  |  |  |  |
|  | CI95% |  |  |  |  |  |  |  |  |  |  |  |  |  |  |  |  |  |

### Cleanup

- Dispose of the used pipets and falcon tubes in the trash.
- Use a soft cloth or test tube cleaning brush, mild dishwashing detergent, and warm water to loosen solids or oils from all laboratory equipment after the completion of an experiment. Thoroughly rinse the items with distilled water and allow them to air dry on clean paper towels or a clean dishtowel.
- Store all clean, dry equipment in a safe location for future use.

## **ASSESSMENT**

### Before You Proceed

- Did you complete all of the required exercises in this lesson? If not, please return to the previous section to finalize your work.
- Are you confident that you’ve achieved the learning objectives listed below? If not, please review the INTRODUCTION content and your responses to the exercise activities.

### Learning Objectives

- Define the source of composition data deviation.
- Describe the residual particle properties using the SEM data.
- Explain the difference in datasets by statistical parameters.
- Analyze composition data semi-quantitatively.
- Relate water quality to sources of the residual composition.

### Exercise Review Questions

#### Question 1

**What are the elements you found in your tap water sample? Is it safe to drink tap water according to your State Department of Health data (e.g., Na < 20 mg/L)? While you may not be able to quantitatively assess it, would you at least suggest an additional analysi(e)s to make?**

The student answers may vary depending on their observation, but the student can mention the necessity of quantitative analysis of bulk water samples (e.g., ion chromatography). The single particle analysis, which is labor intensive and qualitative, has a limitation to assess certain things.

#### Question 2

**Do you see any relationship between residual particle size and composition? If so, describe your observation.**

Model Answer: The student answers will vary but should address the points discussed in SI Sect. S2 – 2.3.

#### Question 3

**An aspect ratio represents a proportional relationship between an image's horizontal length and vertical length. A spherical particle has an aspect ratio of 1:1. From your Data Table 2, describe if the particles you measured have spherical or aspherical shapes.**

Model Answer: The student answers will vary but should address the points discussed in SI Sect. S2 – 2.3.

#### Question 4

**What is the source of deviation in each element in Data Table 3?**

Model Answer: The student answers will vary but should address the points discussed in SI Sect. S2 – 1.3.2.

#### Question 5

**Compare your standard error results in Data Table 3 to the instructor’s results in Manuscript Table 3. Which dataset contains higher statistical uncertainty? Do you think increasing the number of measurements would help minimize the statistical error?**

Model Answer: The student answers will vary but should address the points discussed in SI Sect. S2 – 1.3.2.

### Competency Review Questions

#### Question 6

**The EDX detects and characterizes the X-ray and photon energy/wavelength emitted from a specimen to specify the atom(s) in it. This X-ray/photon emission is triggered by the transfer of [ ] in individual atoms.**

- electron
- neutron
- proton
- nucleus

Model Answer: electron

#### Question 7

**Explain why the atomic percentage is a representative value of EDX analysis.**

Model Answer: It is representative because it accounts for atomic weight and proportion.

#### Question 8

**The EDX on a potassium chloride particle gives you the 1:1 elemental weight percentage of potassium and chloride. Calculate their atomic weight percentages.**

Model Answer:

K: element weight % = 50 %

Cl: element weight % = 50 %

divide each by their atomic weights K (39.10) Cl (35.45) to compute atomic proportion:

K atomic proportion: 50 / 39.10 = 1.28

Cl atomic proportion: 50 / 35.45 = 1.41

divide each by a total atomic weight proportion (TAWP: 2.69 = 1.28 + 1.41) and turn into an atomic percentage:

Na Atomic %: 1.28 / 2.69 * 100 = 47.55 %

Cl Atomic %: 1.41 / 2.69 * 100 = 52.45 %

Thus, we are getting the atomic % for K and Cl.

#### Question 9

**From the SEM-EDX, GC-MS, and NMR results, what do you think what compounds in water act INPs?**

Model Answer: Some impurities were identified in our samples through GC-MS, NMR, and SEM-EDX. In particular, we found a non-negligible amount of methyl/alkyl organic compounds and inorganic compounds (i.e., mineral and salt elements) in the tap water sample.

## **REFERENCES**

- Chase, D.A., Karnjanapiboonwong, A., Fang, Y., Cobb, G. P., Morse, A. N., and Anderson, T. A.: Occurrence of synthetic musk fragrances in effluent and non-effluent impacted environments,  Science of the Total Environment, 416, 253–260, 2012.
- Fulmer, G. R., Miller, A. J. M., Sherden, N. H., Gottlieb, H. E., Nudelman, A., Stoltz, B. M., Bercaw, J. E., and Goldberg, K. I.: NMR chemical shifts of trace impurities: common laboratory solvents, organics, and gases in deuterated solvents relevant to the organometallic chemist, Organometallics, 29, 2176–2179, 2010.
- Giraudeau, P., Silvestre, V., and Akoka, S.: Optimizing water suppression for quantitative NMR-based metabolomics: a tutorial review, Metabolomics, 11, 1041–1055, 2015.
- Heintzman, L.J., Anderson, T. A., Carr, D. L., and McIntyre, N. E.:  Local and landscape influences on PAH contamination in urban stormwater,  Landscape and Urban Planning, 142, 29–37, 2015.
- JEOL: Scanning Electron Microscope A To Z: Basic Knowledge For Using The SEM, No.1101B972C(Ks), Available at https://www.jeolusa.com/RESOURCES/Electron-Optics/Documents-Downloads/scanning-electron-microscope-a-to-z-basic-knowledge-for-using-the-sem, Last visited on May 18, 2022.
- Liu, G., Levien, M., Karschin, N., Parigi, G., Luchinat, C., and Bennati, M: One-thousand-fold enhancement of high field liquid nuclear magnetic resonance signals at room temperature, Nature Chemistry, 9, 676–680, 2017.
- Moon, S.: Chemical and physical properties of atmospheric aerosols (A) a case study in the unique properties of agricultural aerosols (B) the role of chemical composition in ice nucleation during the Arctic spring, Ph.D. Dissertation, Texas A&M University, College Station, TX, USA, 2010.
- Silverstein, R. M., and Bassler, G. C.: Spectrometric identification of organic compounds, Journal of Chemical Education, 39, 546, 1962.

**Copyright Statement** Elemental composition analysis of water residual particles by SEM-EDX by Elise K. Wilbourn, Sarah Alrimaly, Holly Williams, Jacob Hurst, Gregory P. McGovern, Todd A. Anderson, and Naruki Hiranuma is marked with CC0 1.0 Universal Creative Commons license. To view a copy of this license, visit http://creativecommons.org/publicdomain/zero/1.0
