# Supplementary material for: Integrated Science Teaching in Atmospheric Ice Nucleation Research: Immersion Freezing Experiments
Source: J Chem Educ. 2023 Mar 8;100(4):1511–22. doi: 10.1021/acs.jchemed.2c01060 (PMC10100551; doi:10.1021/acs.jchemed.2c01060)
Supplement: Supplementary file 1 — ed2c01060_si_001.zip [file ed2c01060_si_001.zip › SI_Files/SI_Sect_S2_Module_Assessment.docx]

**Supporting Information:**

**Integrated Science Teaching in Atmospheric Ice Nucleation Research:**

**Immersion Freezing Experiments**

Elise K. Wilbourn^1,♦^, Sarah Alrimaly^1,♦^, Holly Williams^1^, Jacob Hurst^2^, Gregory P. McGovern^2^,

Todd A. Anderson^3^, and Naruki Hiranuma^1,^*

^1^Dept. of Life, Earth, and Environmental Sciences, West Texas A&M University, Canyon, TX, 79016

^2^ Dept. of Chemistry and Physics, West Texas A&M University, Canyon, TX, 79016

^3^ Dept. of Environmental Toxicology, Texas Tech University, Lubbock, TX, 79416

^♦^These authors equally lead and contributed to this work

*Corresponding author ([nhiranuma@wtamu.edu](mailto:nhiranuma@wtamu.edu))

# **SI Section S2**

The assessments of each module and associated students’ feedback in the classroom setting are discussed and provided in Sect. 1 and Sect. 2 below, respectively.

# 1. Formal module assessment

Information regarding the assessed area, target, method, and result for each module and outcome are provided below.

## Characterization of bulk water properties

### Learning Outcome 1:

**Assessed Area** – Competency review questions regarding the characterization of the bulk water properties module used in ENVR 1407 Fundamentals of Environmental Science in Spring 2022: Learning Outcome 1 (LO1).

**Target** – The mastery benchmark for lab exercise questions in LO1 was set at 90%. The high threshold was applied due to provision of hands-on support for students by teaching assistants. The same benchmark will be sustained in the future.

**Method** – LO1 was assessed using the characterization of the bulk water properties module and its competency review questions as they included multidisciplinary computational and numerical activities, which were the focus of LO1. The % of correct answers was calculated for all items and then aggregated to determine overall LO1-specific outcomes for environmental science majors (N = 17).

**Result** – Outcome, 93.6 ± 2.5 (avg. ± standard error) %: Benchmarks for LO1 (90 %) were comfortably exceeded by students even accounting for the standard error. Such a small error represents consistency in student performance on LO1, which can be seen as indicating adequate provision of learning materials and support. An evaluation of the missed exercise questions revealed that the majority of the errors were in the mathematical calculations of pH with the negative base 10 logarithms. A supplemental math workshop is envisioned for the future class to improve the students’ math skills, and the instructor will continue teaching math skills to address contemporary and emerging environmental issues via innovative technological training.

### Learning Outcome 2:

**Assessed Area** – Exercise review questions 3 and 4 regarding the characterization of the bulk water properties module in ENVR 1407 Fundamentals of Environmental Science.

**Target** – The benchmark for this LO was a 100% pass rate for the lab assignment with a goal of 80% obtaining a score of 80% or better.

**Method** – The students (N = 18) were required to assess their experimental outcomes in comparison with the model results prepared by the instructor. The level of success in characterizing properties, with 80% of students achieving a score of > 80%, was the standard employed.

It is noteworthy that additional instructions were given in the classroom setting. Briefly, all students assessed HPLC water. Three other water samples (i.e., unfiltered tap, filtered tap, and DI water) were examined by 18 students, and each sample is analyzed by 6 students. In the exercise, the students were encouraged to develop a summary table of their outcomes including pH, conductivity, surface tension, and temperature. This guidance was provided for the students to comprehensively understand the measured water properties.

**Result** – The criterion was met with 83% of students (15/18) achieving an 80% or better score on these exercise questions.

## Freezing of water droplets and ice-nucleating particles

### Learning Outcome 1:

**Assessed Area** – Competency Review Questions #11-14 of the freezing of water droplets and ice-nucleating particles module were assessed in ENVR6303 Advanced Numerical Analysis.

**Target** – The mastery benchmark for LO1 was set at 80% for these lab exercise questions.

**Method** – LO1 was assessed using four lab competency exercise questions. We picked these questions as they comprehensively examined students’ mathematical analysis skills in interdisciplinary science, which are the focus of LO1. The percentage of correct answers was calculated for all items and then aggregated to determine overall LO1-specific outcomes. Only environmental science majors were evaluated (N = 5).

**Result** – Outcome, 85.8 ± 3.9 (avg. ± standard error)%: The students performed well on the assignment pertaining to this outcome, and the benchmark for LO1 was exceeded. This number is a few % lower as compared to other exercises, representing the complexity of the exercise, but the overall standard error is not substantial. An evaluation of the missed exercise questions revealed that the majority of the errors were in the mathematical calculations of *n*_ice_(*t*) and CI95%. An instructor may need to support teaching math skills to engage students in relevant contemporary and emerging environmental issues.

### Learning Outcome 2:

**Assessed Area** – ENVR 6303 Advanced Numerical Analysis: Review questions 1 and 2 of the freezing of water droplets and ice-nucleating particles module, in which students describe research data and outcomes in tables and figures with statistical defensibility.

**Target** – The students’ performance on exercise questions was assessed by tracking percentages of students that correctly answered relevant LO2-related questions. These questions can then be aggregated and sorted by LO with a target set at the pass/fail threshold, ≥ 70% at WTAMU, for correctly answering LO2-related questions. This mastery benchmark is appropriate as the students have varied academic backgrounds and for many students, it was their first time using micro-pipettes.

**Method** – Students’ (N = 5) ability to assess their experimental outcomes by transforming tabular data into graphs with the instructor and teaching assistants providing individualized hands-on instruction during measurements and associated data analysis was assessed. Data resulting from student exercises, summary figures of their outcomes including *FF*(*T*), *C*_INP_(*T*), and CI95%(*T*), were employed. Students were expected to learn to the level of being able to analyze outcomes, evaluate the accuracy, and demonstrate an understanding of statistical defensibility, skills required in future careers. The figures are shown in the main manuscript **Fig. 4** in comparison to the model results.

**Result** – Outcome 2 benchmarks were exceeded and surpassed. Some students narrowly met the target. Some students struggled with word problems and still demonstrated weak math skills. The students that took fundamental numerical analysis and calculus courses performed better than those that did not. An instructor will encourage all environmental science students to take these courses that provide needed mathematical skills and problem-solving skills (i.e. development of defensible assumptions).

## Elemental composition analysis of water residual particles by SEM-EDX

### Learning Outcome 1:

**Assessed Area** – Competency review question #8 of the elemental composition analysis of water residual particles by SEM-EDX module in ENVR6092 Earth and Atmospheric Chemistry: LO1 which encompasses the majority of assessment material for this part of the module.

**Target** – The mastery benchmark for LO1 was set at 80% on the competency review question #8 (the model answer is provided in **SI Sect. 1**).

**Method** – LO1 was assessed using one extensive competency review question regarding atomic percentage calculation. We picked this exercise as it comprehensively examined students’ numerical and computational analysis skills in environmental chemistry, which are the focus of LO1. The percentage of correct answers was calculated for calculation processes, as well as the final answer, and then aggregated to determine overall LO1-specific outcomes for environmental science majors (N = 5).

**Result** –Outcome, 93.3 ± 4.1 (avg. ± standard error)%: The benchmark for LO1 was comfortably exceeded by students. These lab exercises were offered in ENVR6092 for the first time. The instructor and teaching assistants will keep offering hands-on support, technical demonstration, clarification of objectives/contents, and pre-lab hypothesis formulation opportunity to maintain positive outcomes in the future.

### Learning Outcome 2:

**Assessed Area** – The exercise review questions 4 and 5 of the elemental composition analysis of water residual particles by SEM-EDX module, which involves the interpretation of research data and outcomes in tables with statistical defensibility.

**Target** – The goal for the LO is a 100% pass rate with 80% passing with a score of 80% or better. The minimum score is 70%.

**Method** – The students were required to work individually with the support of the instructor and teaching assistants to complete the exercise questions on the report. The students were allowed to discuss their data and outcomes with other students and re-assess their own reports based on the input from their peers. The reports were evaluated for organization, understandability, clarity, and factual basis. These are real-world standards applied to descriptions addressing complex issues that are written in STEM fields.

**Result** – The LO goal was exceeded with 80% of the students exceeding the score of 80% or better on the exercise questions. Missed questions were student-specific based on their academic and interest background. For example, students with weak chemistry backgrounds struggled with the exercise. The instructor has developed a basic skills pre-test to be given at the beginning of the class to identify areas where he needs to expand his lecture or have workshops to level the students for the course. **The students were able to recognize that the source of observed deviation in the SEM-EDX results may have stemmed from the size of particles, degree of agglomeration, and stability of the electron beam. Other concerns are summarized in Sect. 2.3.**

**The students also compared their standard error results in Data Table 3 (shown below) to the instructor’s results in Manuscript Table 5. A detailed discussion of the students’ results vs. model results is provided in Sect. 3.4.1 in the main manuscript.**

**Student Data - Data Table 3.** Analysis of SEM-EDX data.

| Water Type | Parameters |  | Cross-section size |  | Atomic % | | | | | | | | | | | | | |
| --- | --- | --- | --- | --- | --- | --- | --- | --- | --- | --- | --- | --- | --- | --- | --- | --- | --- | --- |
|  |  |  | Average (µm) |  | C | N | O | Na | Mg | Si | P | S | Cl | K | Ca | Mn | Fe | Zn |
| Tap | *µ* |  | 1.94 |  | 7.90 | 0.00 | 72.44 | 8.32 | 6.46 | 1.11 | 0.03 | 0.86 | 2.16 | 0.21 | 0.15 | 0.03 | 0.30 | 0.04 |
|  | *n* |  | 100 |  | 100 | 100 | 100 | 100 | 100 | 100 | 100 | 100 | 100 | 100 | 100 | 100 | 100 | 100 |
|  | *σ* |  | 0.68 |  | 3.36 | 0.00 | 9.98 | 6.43 | 1.78 | 0.34 | 0.03 | 0.32 | 4.50 | 0.07 | 0.07 | 0.03 | 0.22 | 0.06 |
|  | ***ε*** |  | **0.07** |  | **0.34** | **0.00** | **1.00** | **0.64** | **0.18** | **0.03** | **0.00** | **0.03** | **0.45** | **0.01** | **0.01** | **0.00** | **0.02** | **0.01** |
|  | *t* |  | 1.98 |  | 1.98 | 1.98 | 1.98 | 1.98 | 1.98 | 1.98 | 1.98 | 1.98 | 1.98 | 1.98 | 1.98 | 1.98 | 1.98 | 1.98 |
|  | CI95% |  | 0.14 |  | 0.67 | 0.00 | 1.98 | 1.27 | 0.35 | 0.07 | 0.01 | 0.06 | 0.89 | 0.01 | 0.01 | 0.01 | 0.04 | 0.01 |
| Ultrapure | *µ* |  | 2.33 |  | 28.29 | 0.00 | 65.15 | 0.08 | 1.65 | 1.14 | 0.03 | 0.20 | 0.01 | 0.03 | 3.08 | 0.01 | 0.17 | 0.17 |
|  | *n* |  | 31 |  | 31 | 31 | 31 | 31 | 31 | 31 | 31 | 31 | 31 | 31 | 31 | 31 | 31 | 31 |
|  | *σ* |  | 0.89 |  | 8.56 | 0.00 | 6.77 | 0.07 | 1.94 | 2.50 | 0.05 | 0.98 | 0.01 | 0.03 | 1.56 | 0.02 | 0.14 | 0.08 |
|  | ***ε*** |  | **0.16** |  | **1.54** | **0.00** | **1.22** | **0.01** | **0.35** | **0.45** | **0.01** | **0.18** | **0.00** | **0.01** | **0.28** | **0.00** | **0.02** | **0.01** |
|  | *t* |  | 2.04 |  | 2.04 | 2.04 | 2.04 | 2.04 | 2.04 | 2.04 | 2.04 | 2.04 | 2.04 | 2.04 | 2.04 | 2.04 | 2.04 | 2.04 |
|  | CI95% |  | 0.33 |  | 3.14 | 0.00 | 2.48 | 0.03 | 0.71 | 0.92 | 0.02 | 0.36 | 0.00 | 0.01 | 0.57 | 0.01 | 0.05 | 0.03 |

# 2. Direct feedback

## 2.1. Characterization of bulk water properties

### Survey Questions:

The five post-module exercise survey questions asked of students were:

Question 1. What are typical problems that you encounter during preparing the pH, conductivity, and surface tension experiments?

Question 2.  What are typical problems that you encounter as you are performing the experiments after the samples are prepared? Any suggestions for future improvement in the module?

Question 3. Before you started the experiment, did you expect there to be a relationship between pH and electrical conductivity?

Question 4. (a) Discuss what bulk properties have an influence on ice nucleation activity by looking at the table below. (b) explain sources of error in the results below (standard deviations).

|  | pH | Conductivity (µS cm^-1^) | Surface Tension (dynes cm^-1^) | *T* (ᵒC) | IN activity |
| --- | --- | --- | --- | --- | --- |
| Unfiltered-tap water | 7.5 ± 0.1 | 859.8 ± 23.7 | 73.8 ± 0.5 | 19.3 ± 0.9 | High |
| Filtered-tap water | 8.0 ± 0.3 | 1006.3 ± 47.6 | 73.0 ± 0.7 | 18.7 ± 1.5 | Middle |
| HPLC water | 5.6 ± 0.3 | 22.4 ± 16.4 | 75.9 ± 2.1 | 18.9 ± 1.1 | Low |

Question 5.  Would you expect the surface tension of tap water to be lower than that of ultrapure water (based on the theory)? Is the surface tension of tap water lower than that of ultrapure water (based on your observation)? If yes, explain what this suppression tells you.

### Feedback Responses:

The student response varied (n = 8), but example responses include the followings:

Response example for Q1.

- Need to make sure the water samples in each type are at the same temperature as each other during all steps to obtain comparable results
- Need to make sure the probes are calibrated correctly, and the probe tip is undamaged
- Occasionally difficult to put glass straw through the stopper of the tensiometer

Response example for Q2.

- Some tubes weren’t clean. Sterilized and disposable tubes can be used.
- The water samples weren’t kept at the same temperature.
- Material/ions had leached into the pure water from long-term storage in an improper container. Preparing water stock samples can eliminate the storage issue.

Response example for Q3.

Only to a very small extent, as conductivity is controlled to a much greater extent by ions other than H^+^

Response example for Q4.

- a) by only looking at the data, it appears that the property driving ice nucleation ability is likely not represented in this table, as there is no direct relationship between the high ice nucleation activity and which parameters are highest or lowest.
- a) High conductivity and ions in tap water samples (as compared to the HPLC water sample) may drive ice nucleation.
- b) sources of error could be due to differences between the different samples tested or due to the instruments used to make the measurements.

Response example for Q5.

The surface tension is lower in the tap water due to the presence of impurities that are capable of acting as surfactants, but the pure water measurements are much more variable (std dev is larger).

## Freezing of water droplets and ice-nucleating particles

### Survey Questions:

The five post-module exercise survey questions asked of students were:

Question 1. What are typical problems that you encounter during the WT-CRAFT experiments?

Question 2. What are the problems in preparation? What steps in preparing the sample could be sources of contamination?

Question 3. Before you started the experiment, did you expect the DI water or tap water to freeze more efficiently at a higher temperature? Explain why. Did your observations match your predictions? If not, explain any sources of possible error.

Question 4. What would we expect to see with the frozen fraction curve and associated error if we employed the 0.05 °C min^-1^ cooling instead of the 1 °C min^-1^ cooling? What about with 3 °C min^-1^? Do you think we should worry about the time dependence of ice nucleation?

Question 5. Compare your DI water frozen fraction curve to the theoretical pure water curves. How do INPs contribute to atmospheric ice formation?

### Feedback Responses:

The student response varied (responding n = 5), but example responses include the followings:

Response example for Q1.

- A typical problem that I experience with the WT-CRAFT system is that occasionally there is a size inconsistency in the droplets due to manual micro-pipette operation to generate individual droplets.
- Speed of preparation can introduce contaminants, causing early freezing that must be distinguished from “real” data.
- Some typical problems I encounter during my WT-CRAFT experiments are:
  - the thermostat falls onto the plate, forcing a restart.
  - the typical problem for the WT-CRAFT for me is the long-operation time.
- Problems I have encountered include ambiguous video interpretation and disrupting sample plate during insertion into the WT-CRAFT machine.

Response example for Q2.

- Sample preparation:
  - static cling makes the sample preparation difficult.
  - Suspension samples may not be homogenized over time.
- Plating:
  - The Vaseline layer must be even.
  - Vaseline must be spread very quickly.
  - The pipette tip may touch Vaseline without noticing.
  - Water droplets may stick to the outside of the pipette tip and fall on the plate.
  - The grid must be even (and with the correct number of droplets).
- Loading the plate into the WT-CRAFT system:
  - Operators must hold the plate correctly without touching the plate.
  - Lamp falls.
  - Operators must place the plate without dropping it into the well/disturbing the grid.
  - The thermostat probe must be balanced and occasionally falls into the sample, requiring a complete reset (if enough sample is available – otherwise, no data for that sample).

Response example for Q3.

- Tap water, as particle matter in the water (naturally occurring or introduced through interaction with pipes/plumbing/faucet/etc.) can act as INPs
- Yes (note – unless the operator is introducing extreme amounts of contaminants with the purified water, I doubt they will ever see the tap water freezing colder than the DI water).
- Tap water is less 'pure' than DI-grade water and therefore should freeze at a higher temperature because there are more nucleation sites for water to freeze upon. If this is not observed in experiments, possible sources of error are contamination of DI water or errors in the temperature probe.

Response example for Q4.

- 0.05 °C min^-1^ cooling would result in a multiple-hour experiment, causing evaporation of the droplets as no humidified air is introduced into the chamber and the average RH in Amarillo is so low.
- 3 °C min^-1^ risks missing freezing events and potentially does not allow heat transfer at the same rate between the droplets and the plate, so the FF curve may shift inaccurately to the left.
- The time dependence of the actual nucleation events is so fast as to be negligible when watching in real-time for freezing to occur in videos, but the cooling rate should be optimized to ensure the rate of heat transfer between the droplet and cooling system is correct (and thus that data is as correct as it can be).

Response example for Q5.

DI water is still not quite as clean/free of contamination as ultrapure water and freezes slightly warmer than theoretical ultrapure water freezing homogeneously. It is nearly impossible to have pure water in a non-lean room-equipped lab setting without the introduction of contaminants. INPs induce ice formation in the atmosphere at temperatures above -36 °C.

## Elemental composition analysis of water residual particles by SEM-EDX

### Survey Questions:

The five post-module exercise survey questions asked of students were:

Question 1. What are typical problems that you encounter during the SEM-EDX experiments?

Question 2. What are the problems in preparation? What steps in preparing the sample could be sources of errors/contamination?

Question 3. Before you started the experiment, did you expect to see any relationship between residual particle size, shape, and composition? If so, describe your hypothesis.

Question 4. An aspect ratio represents a proportional relationship between an image's horizontal length and vertical length. A spherical particle has an aspect ratio of 1:1. What do you typically observe on your SEM data? Describe if the particles you usually measure have spherical or aspherical shapes.

Question 5. In your EDX elemental atomic % analysis, what is the source of deviation in each element (e.g., sodium Atomic % can vary for different particles, right?)? Do you think increasing the number of measurements would help minimize the statistical deviation/error?

### Feedback Responses:

The student response varied (responding n = 3), but example responses include the followings:

Response example for Q1.

- Typically the only problem I face is making sure that the image quality is nice and that the count of electrons per second on the machine is virtually consistent for all particles analyzed.
- Finding a particle that is ≤ 1um is very hard and time-consuming in the beginning.
- Since we only examined the 2D images, the interpretation of agglomerated particles seems limited in this SEM-EDX analysis.
- To get a reasonable SEM image, one needs to control many settings, such as the focus, brightness, contrast, and even how the background looks. Very labor intensive.

Response example for Q2.

In preparation, I am always concerned about contamination (e.g., while cutting a sample substrate and transporting it onto the SEM stage with static).

Response example for Q3.

- No, I did not expect to see any correlation between particle size and composition considering that the particles can come as a result of larger particles breaking down.
- I am skeptical about the relationship between residual particle size and composition. I analyzed less than 100 samples. I think more sample analysis needs to be done for me to confidently give a hypothesis.
- Yes. It is interesting to see any relationship between the residual particle sizes and composition. My hypothesis is the large irregular size water residual particles would be mineral- and/or salt-rich.

Response example for Q4.

- I would say that a spherical assumption works most of the time, but some types of particles like salts can produce more rectangular prism-like shapes just due to how they would fracture as a crystal.
- For my data, the X/Y ratio average was around 1.1. Looking at all the samples one by one, the ratio fluctuates in a narrow range (i.e., from 0.9 to 1.1). With this data, I can confidently the shape of my samples was virtually spherical, and the spherical assumption might be valid for the samples I assessed.
- The mineral dominant particle usually has a large aseptic ratio and with aspherical shapes, while organic and salt particles seem almost spherical (i.e., the aspect ratio ~1:1).

Response example for Q5.

- I think that the source of deviation in particle composition stems explicitly from the sources of those particles being different.
- I think doing more EDX would improve the statistical validity of the data. As we use 4 quadrants for our analysis region on our substrate, we are not being biased by any specific regions at least.
- Increasing the measurement of samples definitely helps on improving the accuracy of atomic % and reduces the uncertainty.

# 3. Remark & outlook

This assessment utilized a number of different approaches, including evaluating course laboratory assignments, review questions, and student direct feedback. The instructors assessed two learning outcomes that we measure. Overall students did well in all three modules. The course outcomes exceeded benchmarks in most cases. An action plan and specific assignments have been noted for the future to improve learning outcomes in several areas.

Instructors will continue to seek improvement for our students in problem-solving, hypothesis formulation, mathematics, statistics, and student engagement. Another important area where we continue to seek improvements is in written and oral communication skills, likely in part due to COVID-19 impacts on learning. Hiring more teaching assistants and providing small group training more frequently may enhance student learning.

A key challenge in implementing the developed modules is the wide variety of academic backgrounds that our students have, which can introduce some deviation in the learning outcome. Instructors continue to improve their curriculum assessment artifacts and methods to meet all student needs to the best of their abilities based on future student feedback.

Some students struggle with math problems and still demonstrate, in some cases, weak overall computational math and data interpretation skills. Instructors may add more pre-requisite math courses (especially for Module 2) and require students to take a rigorous math class to improve the computation of their aptitude. Instructors also may update the module introduction and exercise to adopt alternative ways of teaching math.
