# Supplementary material for: Integrated Science Teaching in Atmospheric Ice Nucleation Research: Immersion Freezing Experiments
Source: J Chem Educ. 2023 Mar 8;100(4):1511–22. doi: 10.1021/acs.jchemed.2c01060 (PMC10100551; doi:10.1021/acs.jchemed.2c01060)
Supplement: Supplementary file 1 — ed2c01060_si_001.zip [file ed2c01060_si_001.zip › SI_Files/SI_Sect_S7_data/Figure_S1_data.pdf]

Supporting Information:
Integrated Science Teaching in Atmospheric Ice Nucleation Research:
Immersion Freezing Experiments

Elise K. Wilbourn[1,•], Sarah Alrimaly[1,•], Holly Williams[1], Jacob Hurst[2], Gregory P. McGovern[2], Todd A. Anderson[3], and Naruki Hiranuma[1,*]

[1]Dept. of Life, Earth, and Environmental Sciences, West Texas A&M University, Canyon, TX, 79016
[2]Dept. of Chemistry and Physics, West Texas A&M University, Canyon, TX, 79016
[3]Dept. of Environmental Toxicology, Texas Tech University, Lubbock, TX, 79416

[•]These authors equally lead and contributed to this work
*Corresponding author (nhiranuma@wtamu.edu)

Figure S1 data

A series of images taken at an incremental temperature step of 0.5 °C for 2a_DI (Table S1) is available in this file. A subset of images is used for Fig. S1.

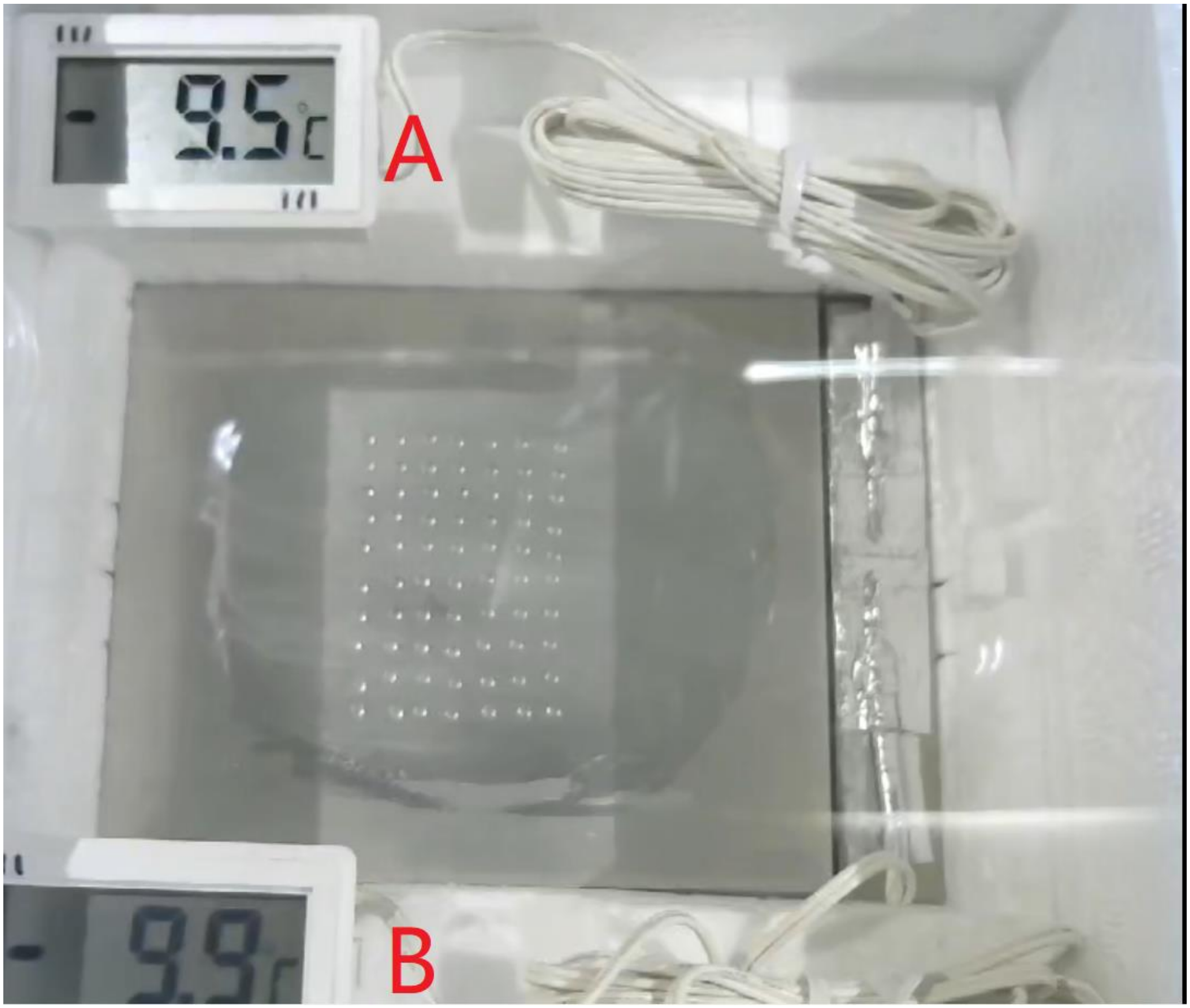

NOTE: The deviation between two temperature sensors (A and B) is always within the experimental temperature uncertainty (± 0.5 °C)

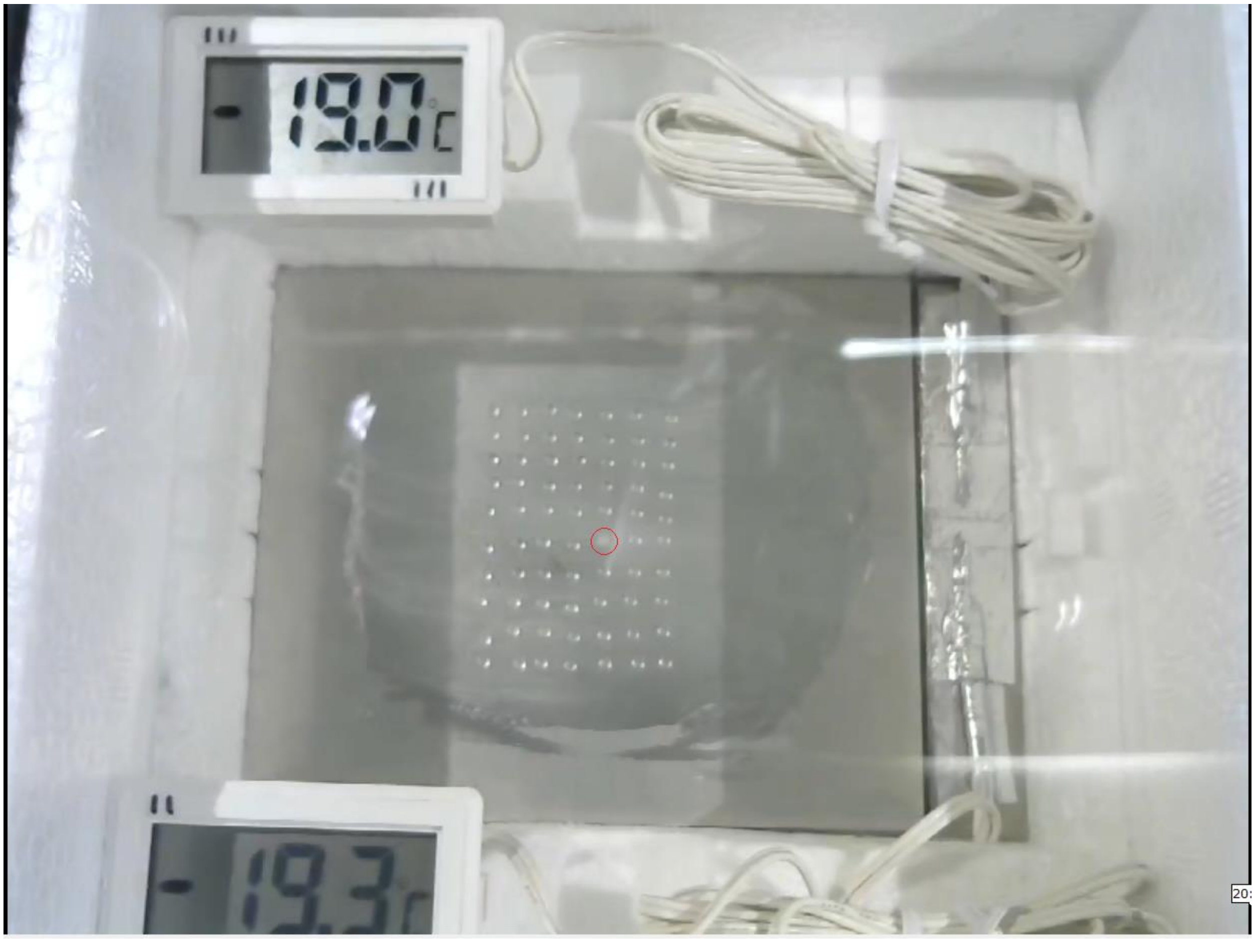

19.0
19.3

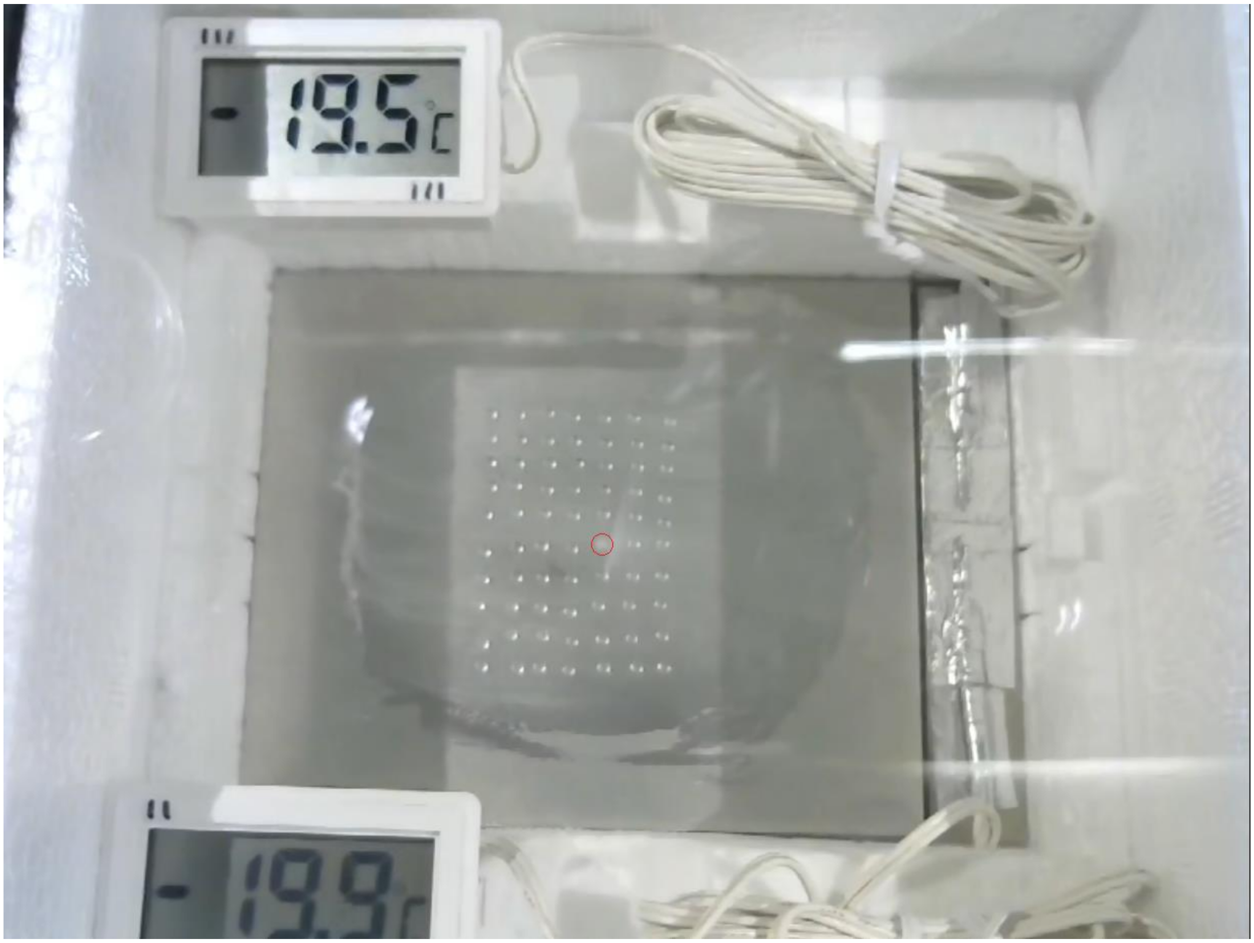
-19.5°C
-19.9°C

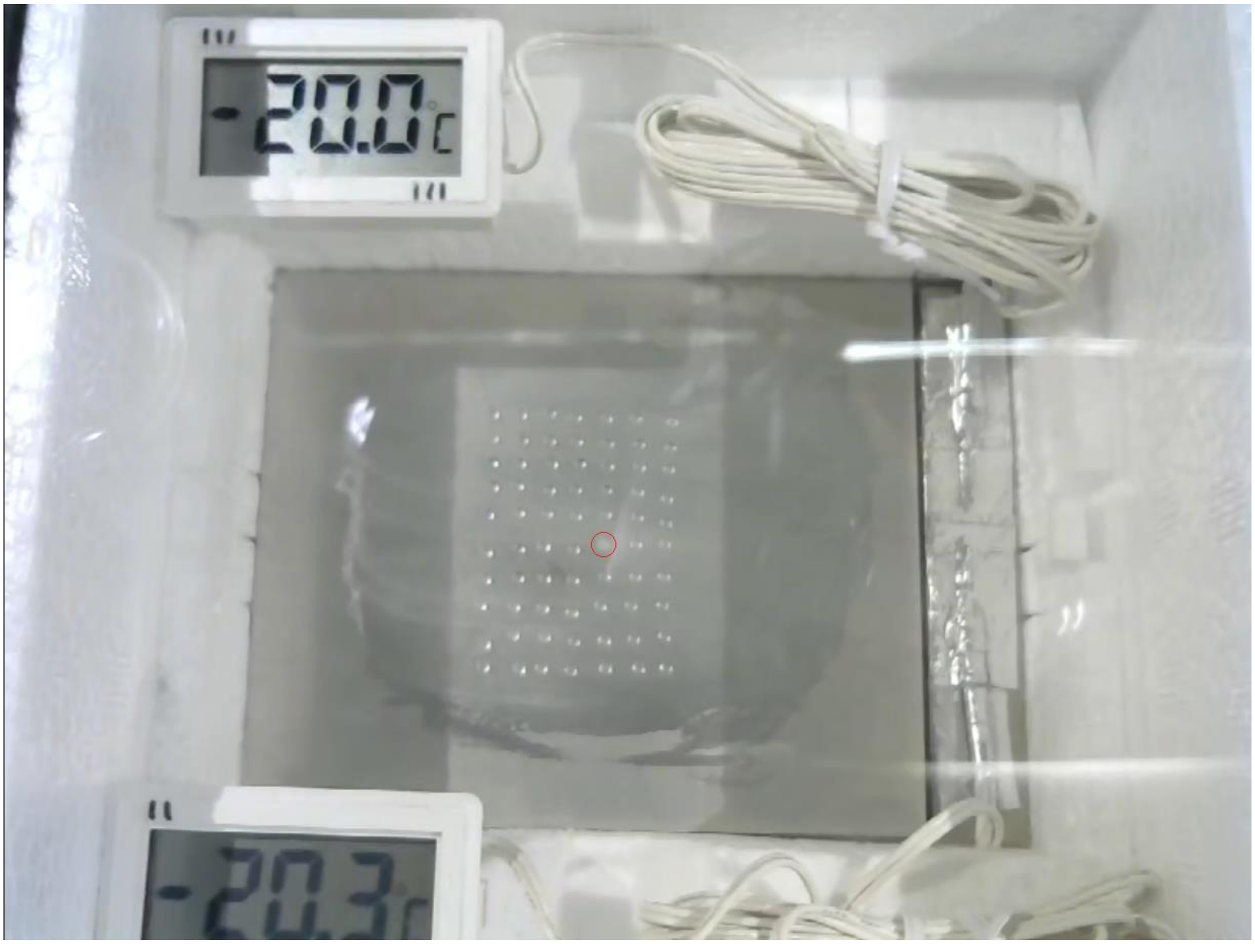
-20.0°C
-20.3°C

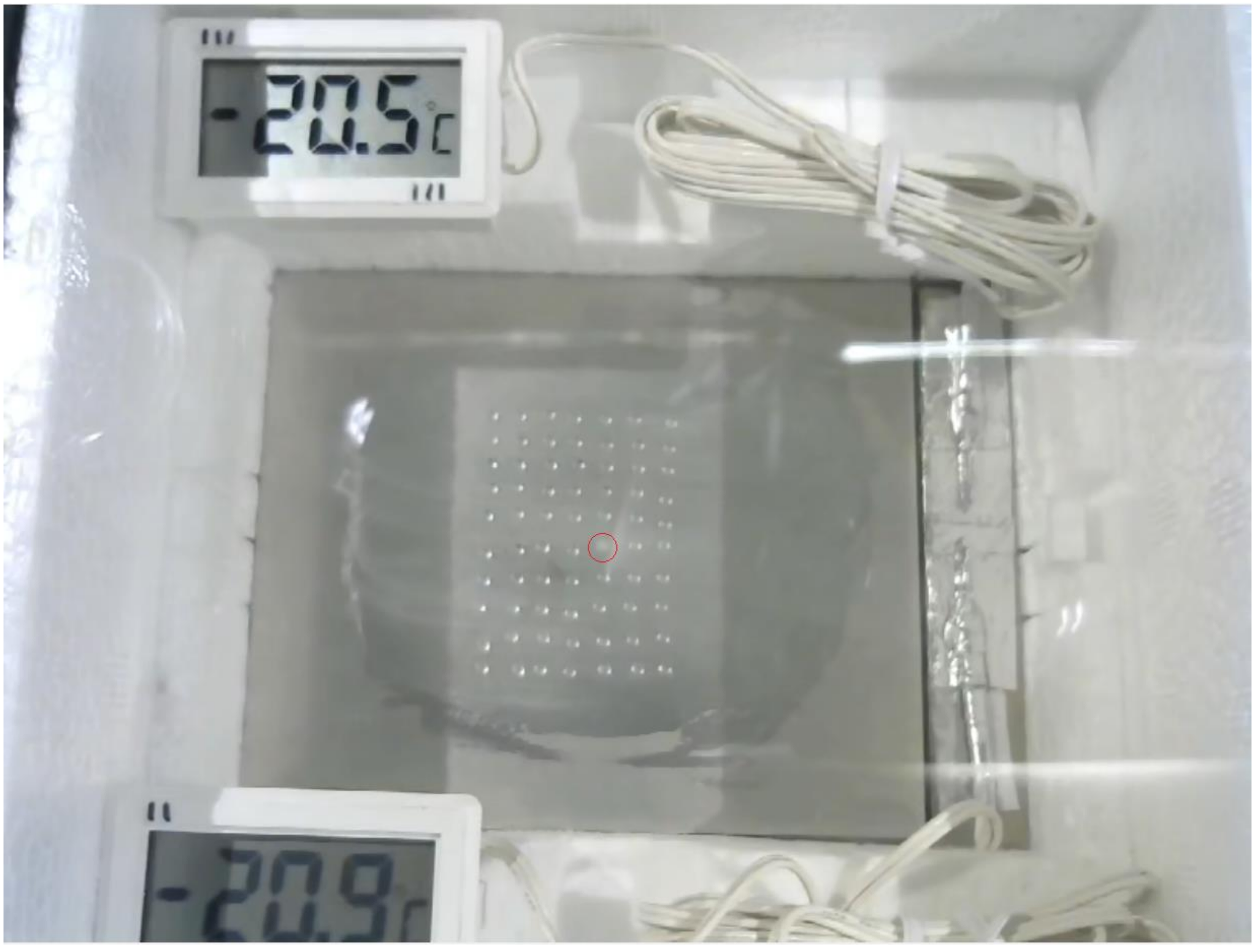
-20.5°C
-20.9°C

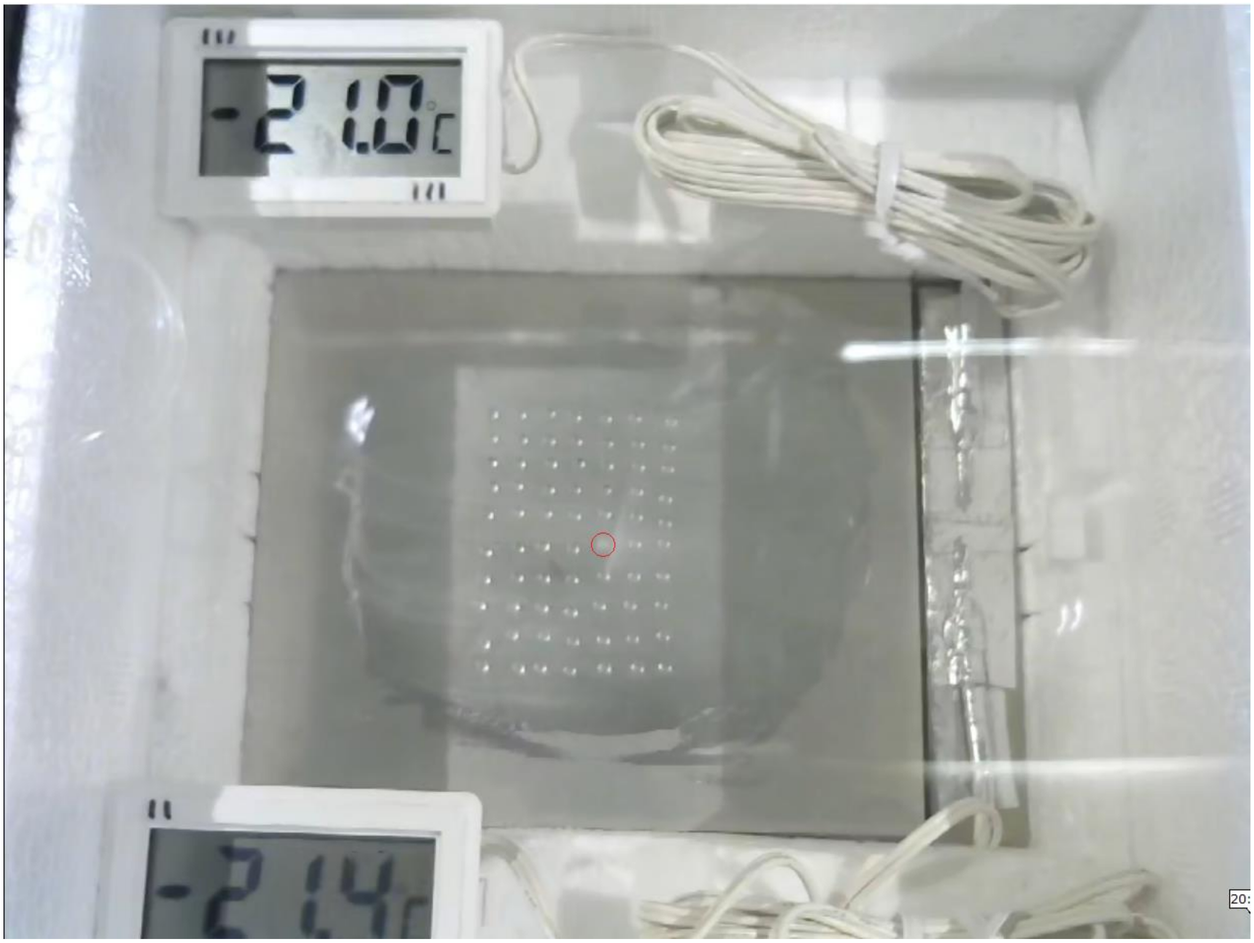
-21.0°C
-21.4°C

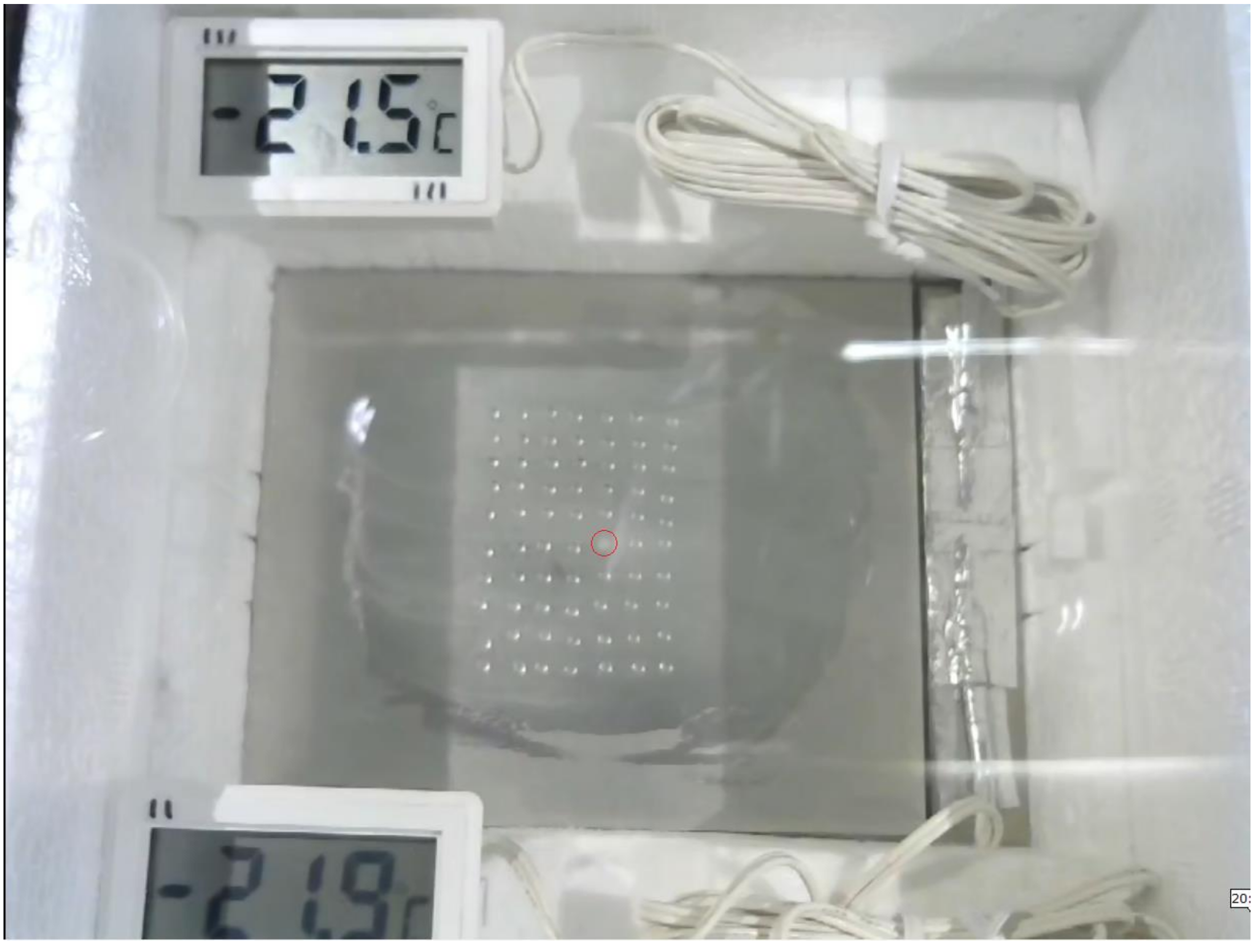
-21.5℃
-21.9℃

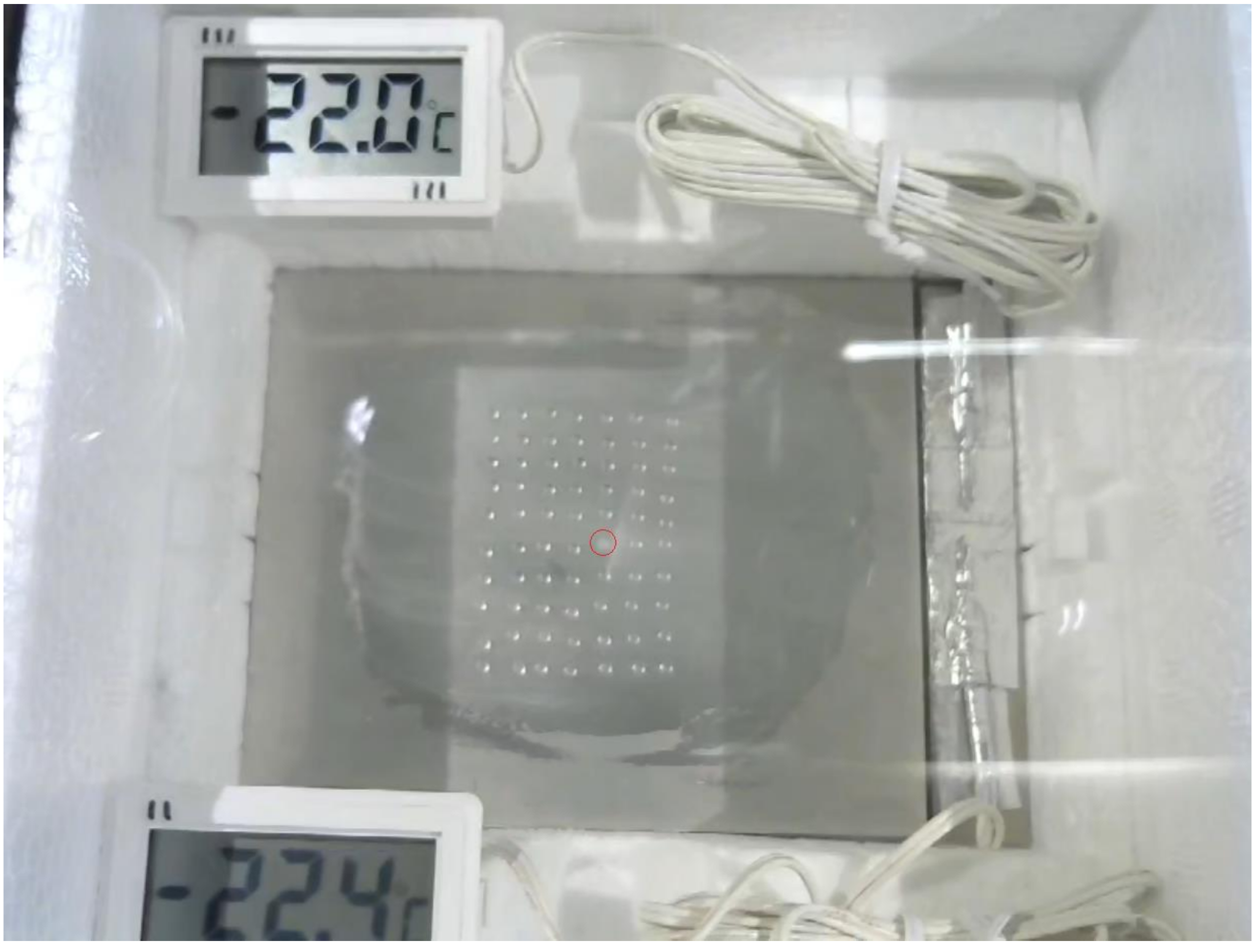
-22.0°C
-22.4°C

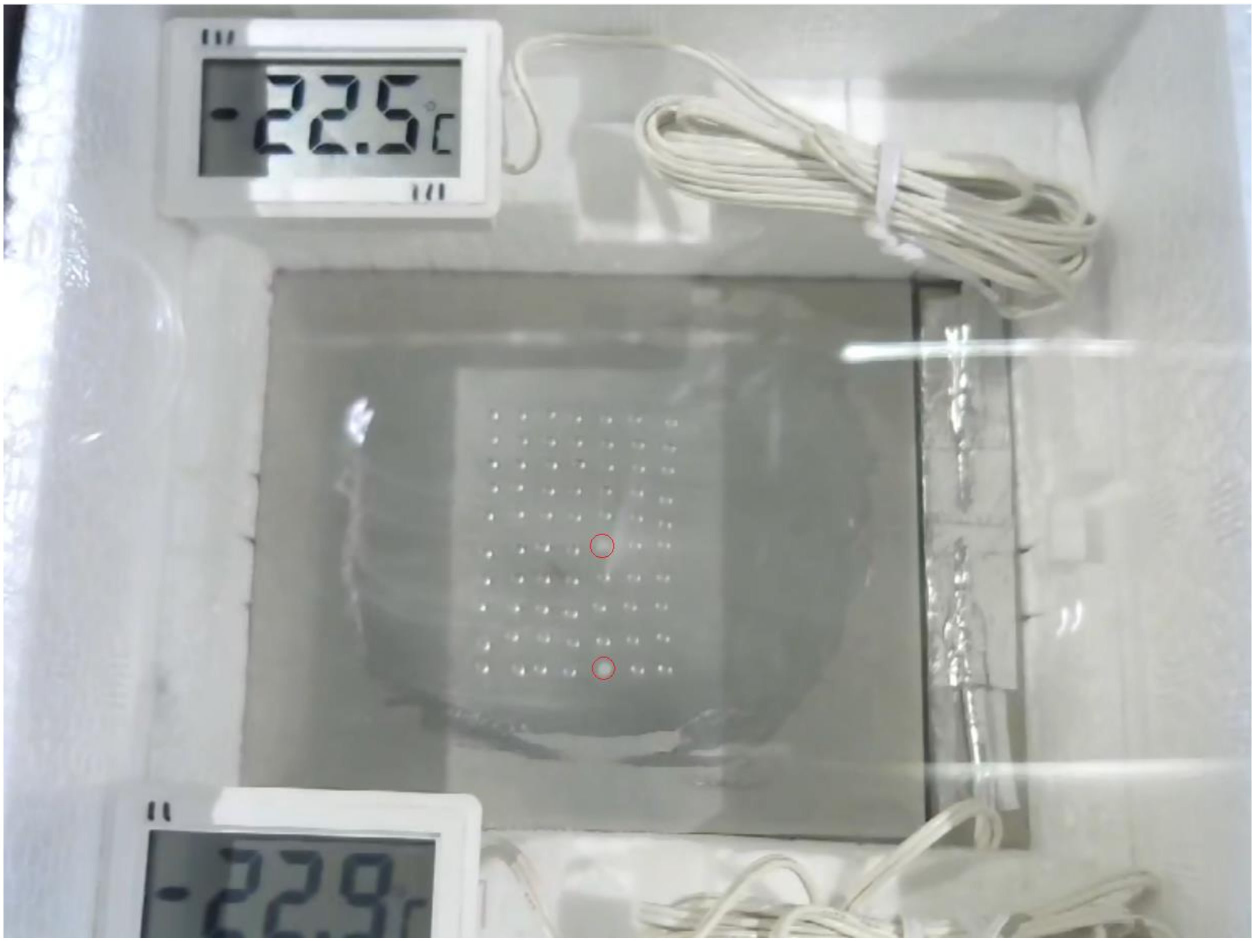
-22.5°C
-22.9°C

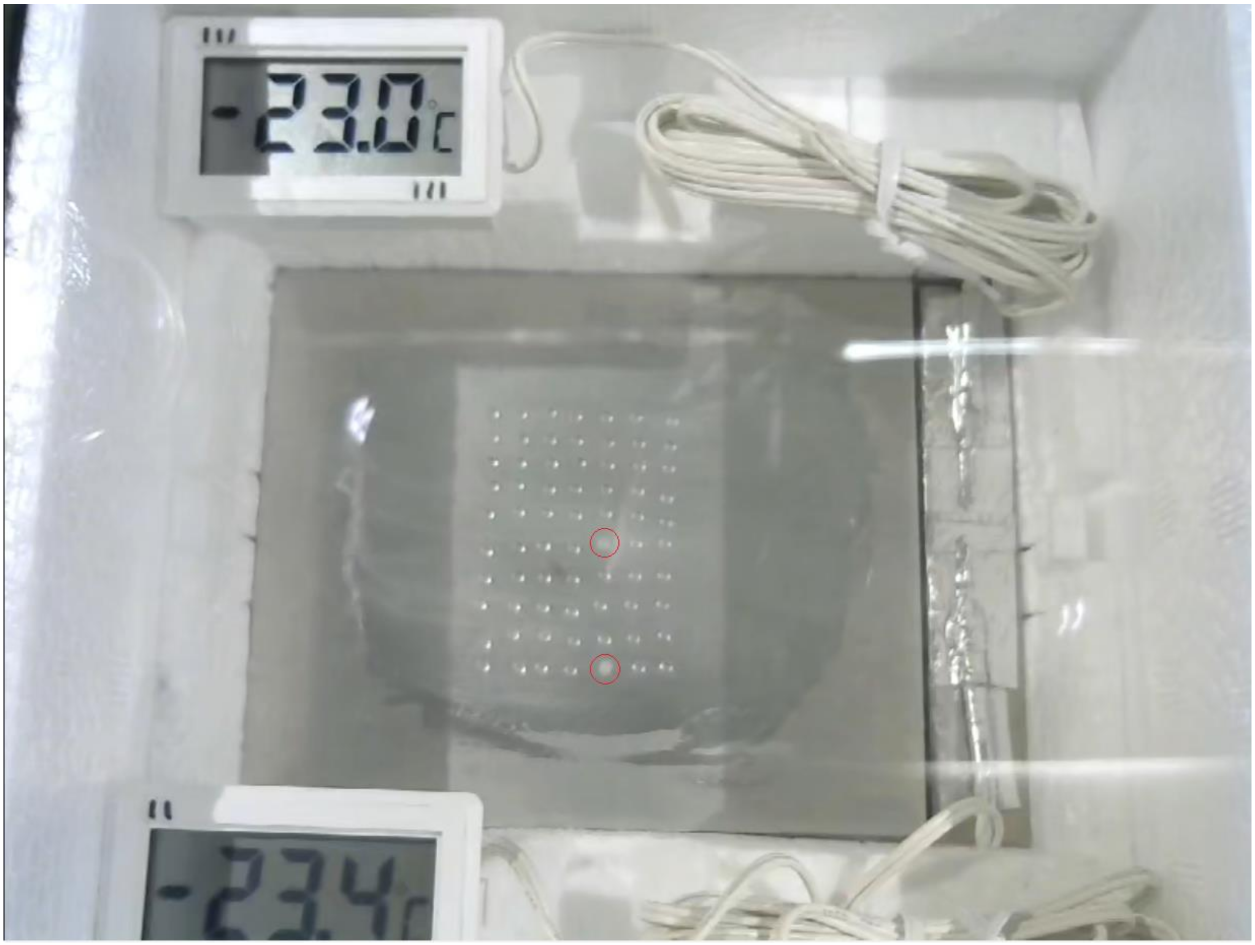
-23.0°C
-23.4°C

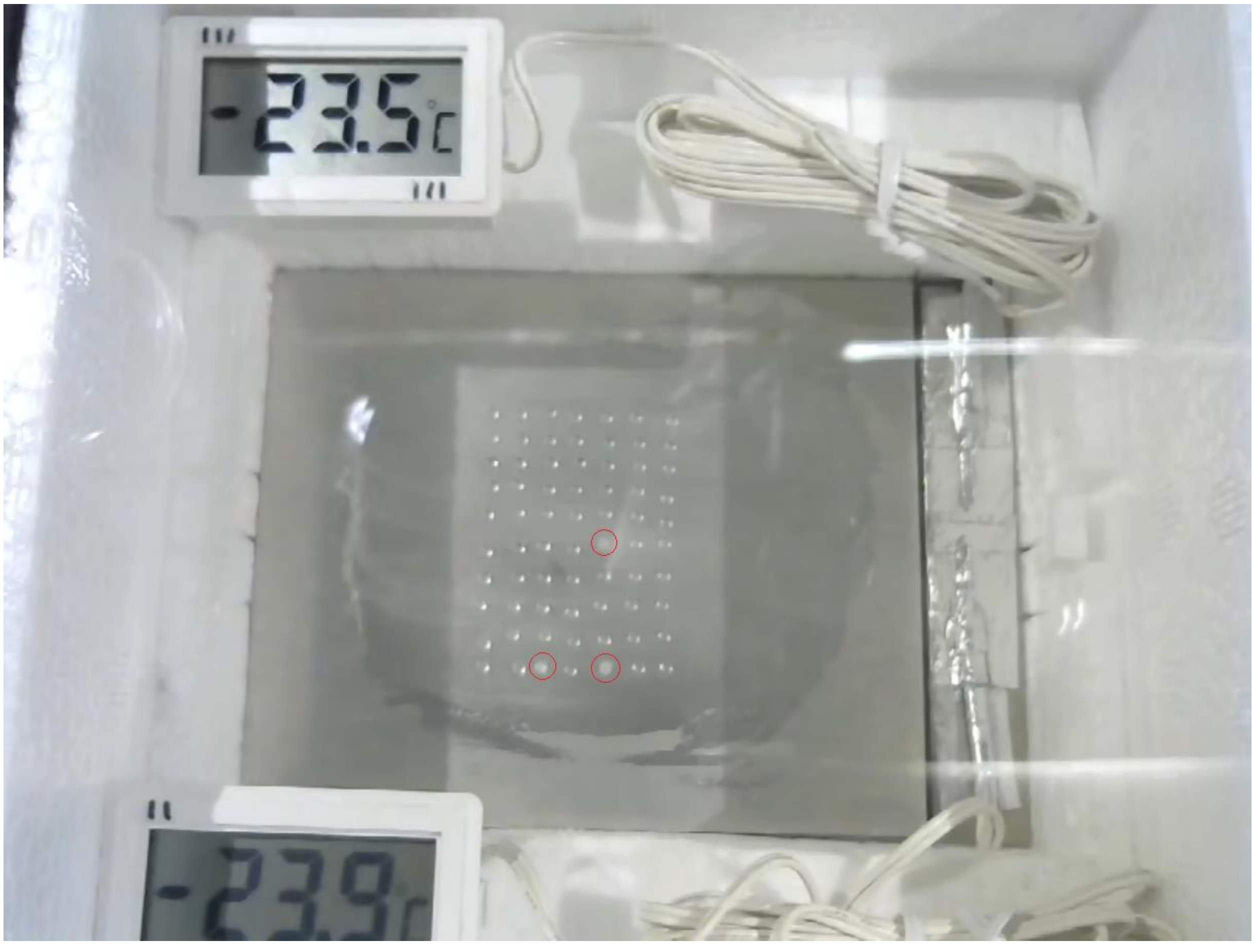
-23.5°C
-23.9°C

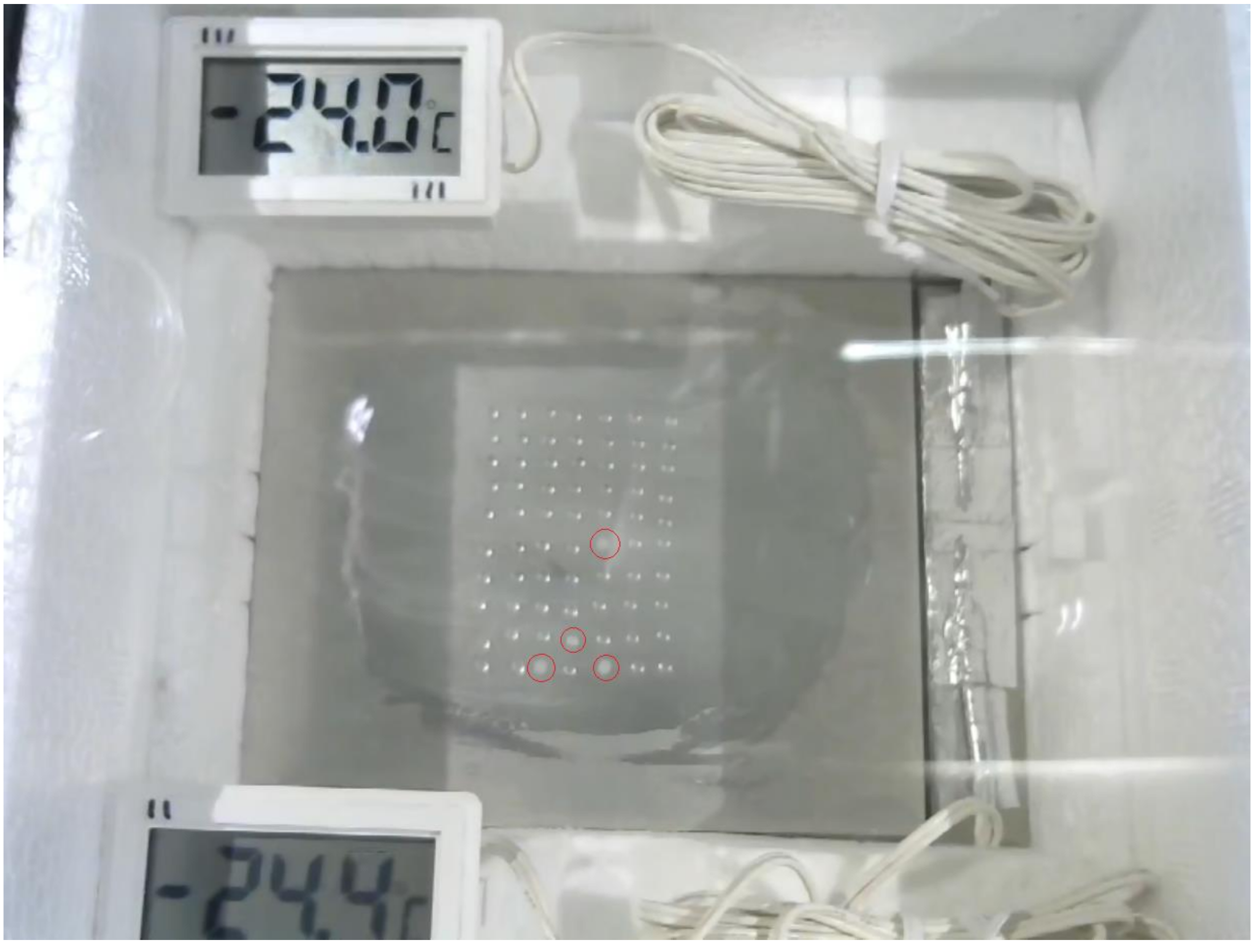
-24.0°C
-24.4°C

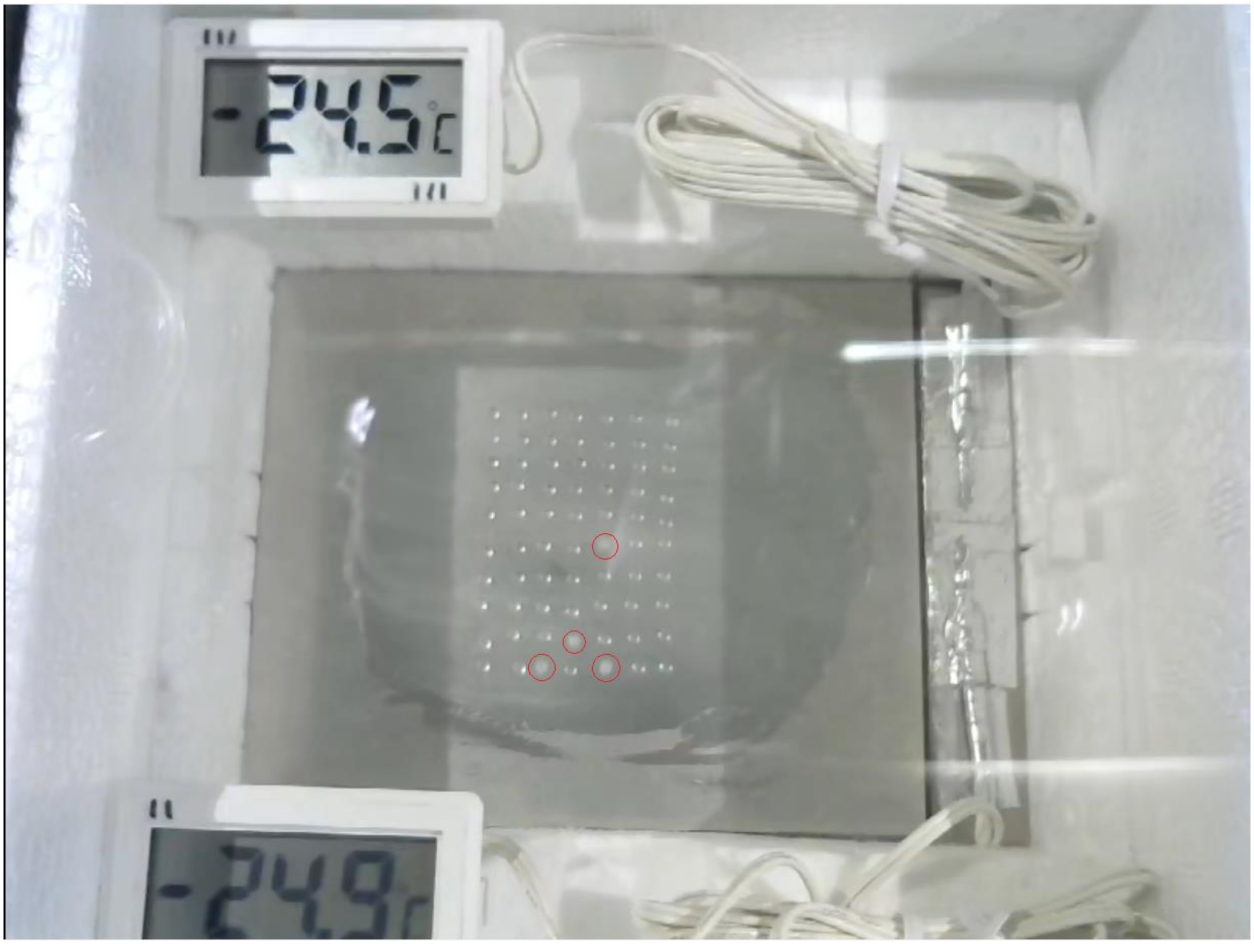
-24.5°C
-24.9°C

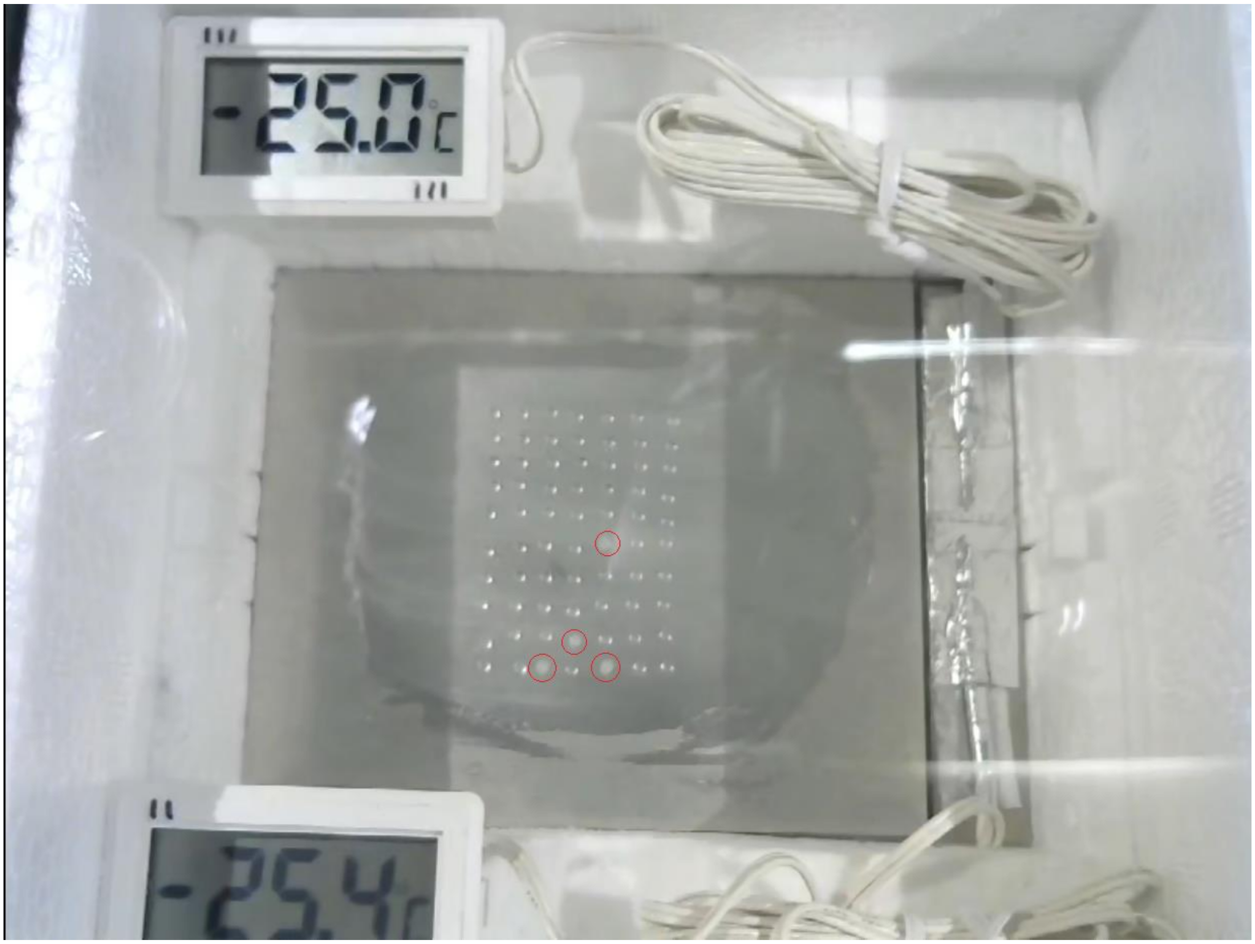
-25.0 C
-25.4 C

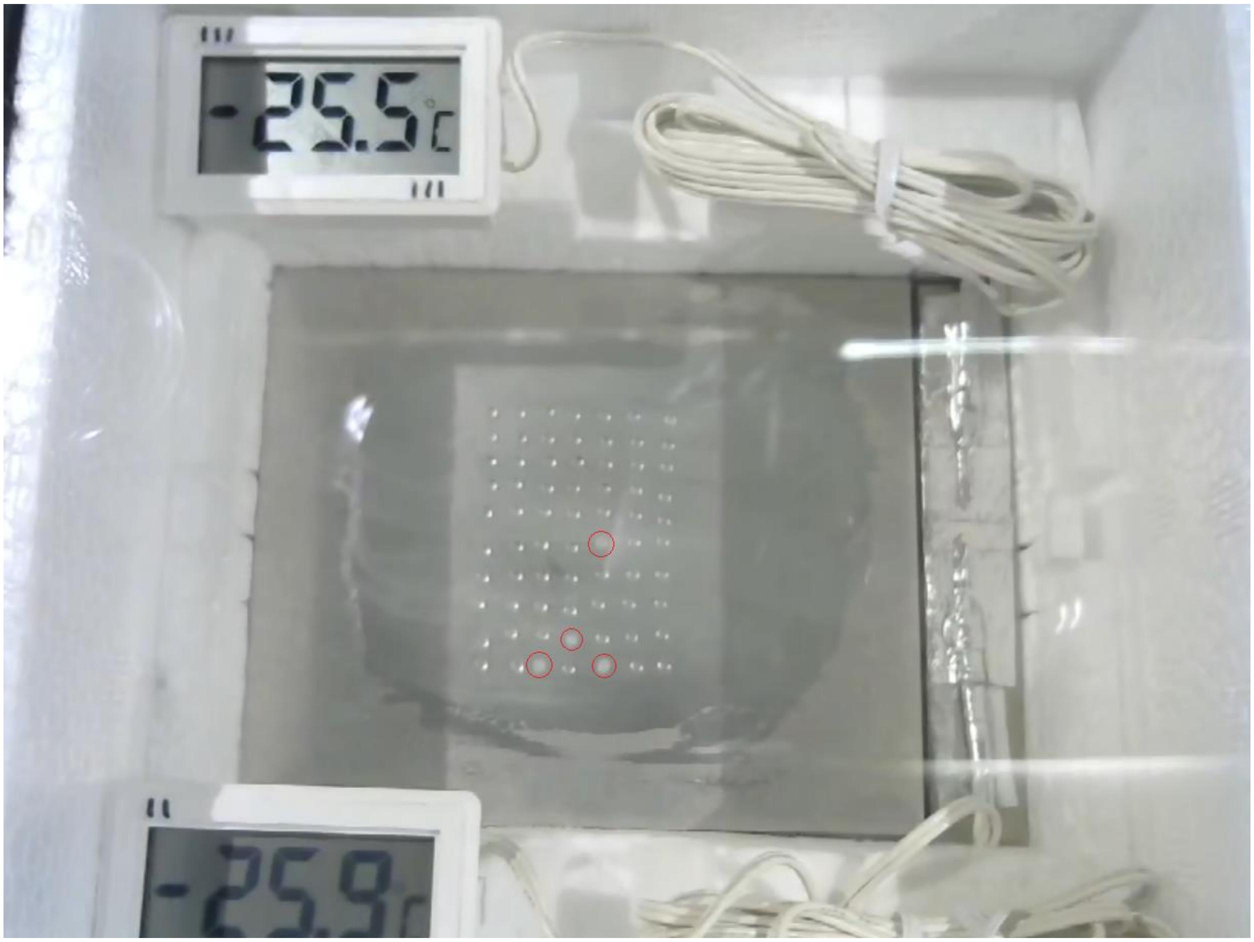

-25.5°C
-25.9°C

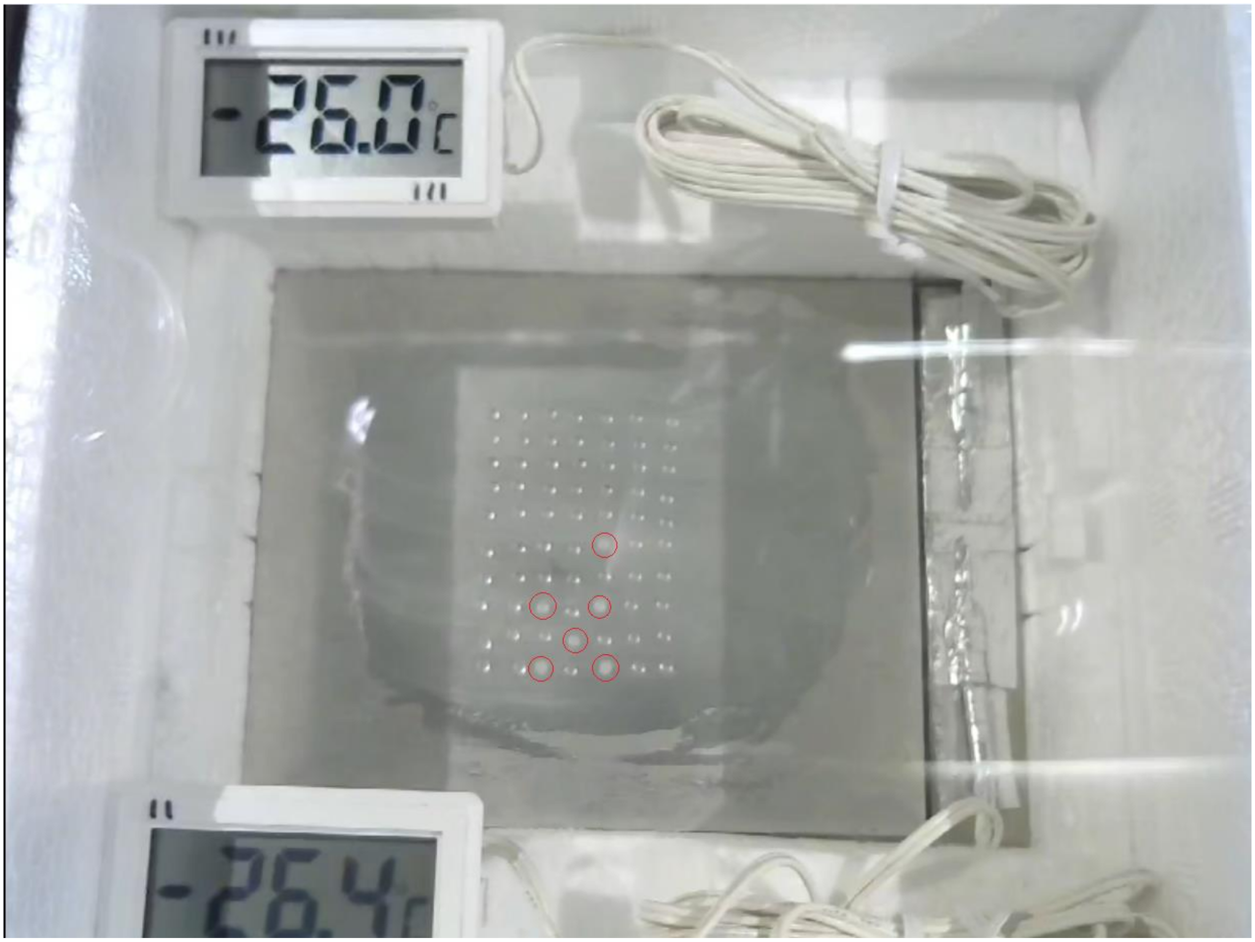
-26.0°C
-26.4°C

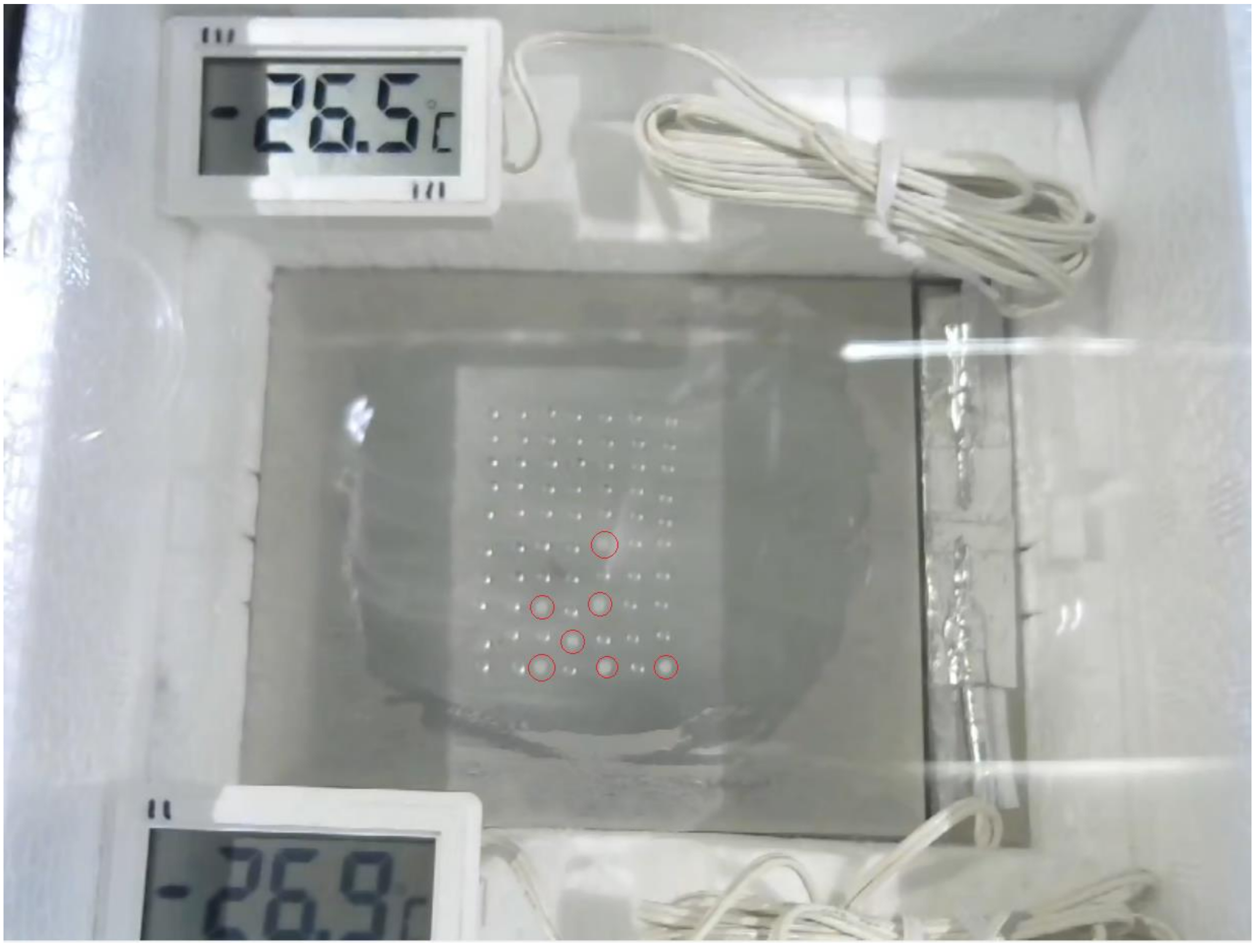
-26.5°C
-26.9°C

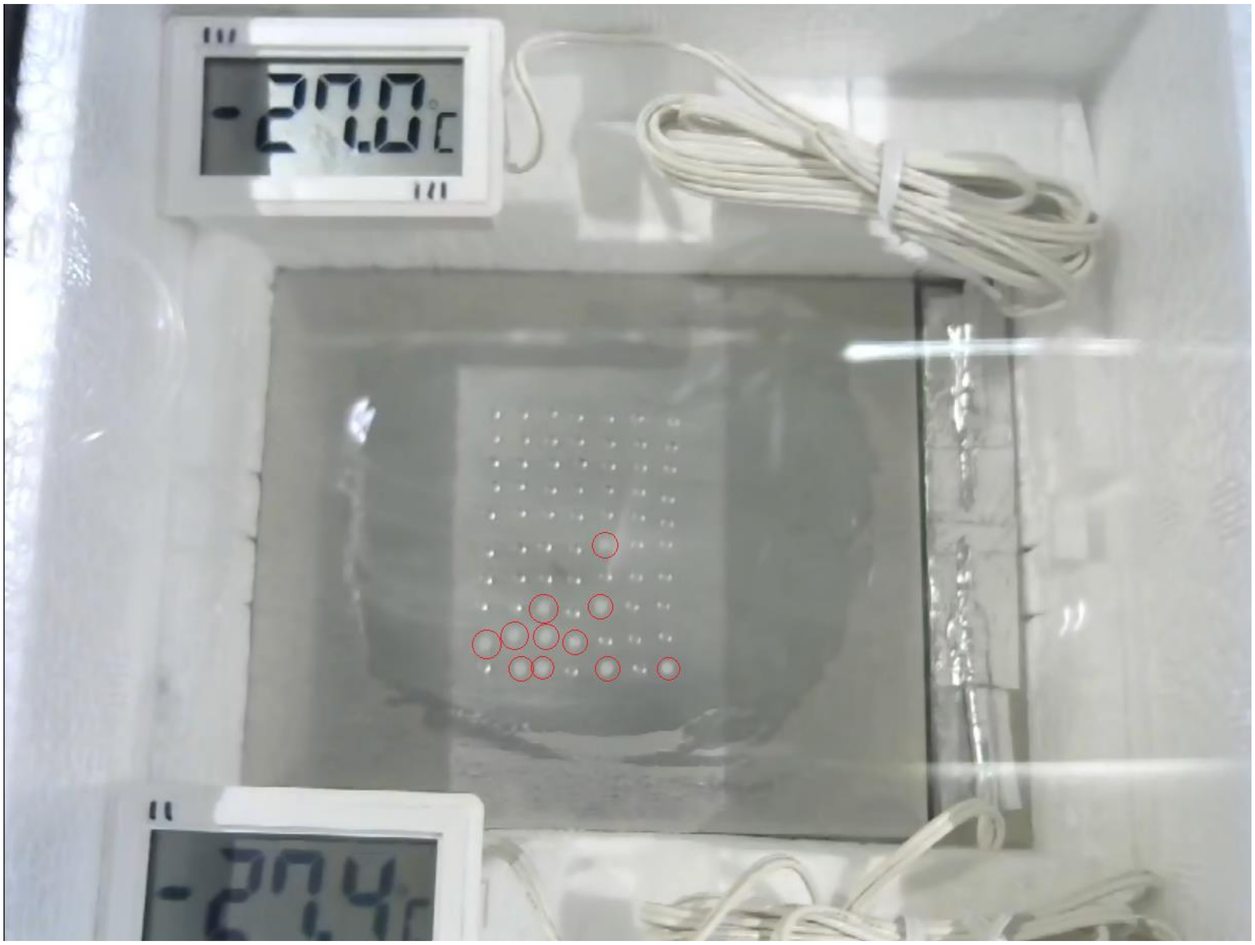
-27.0°C
-27.4

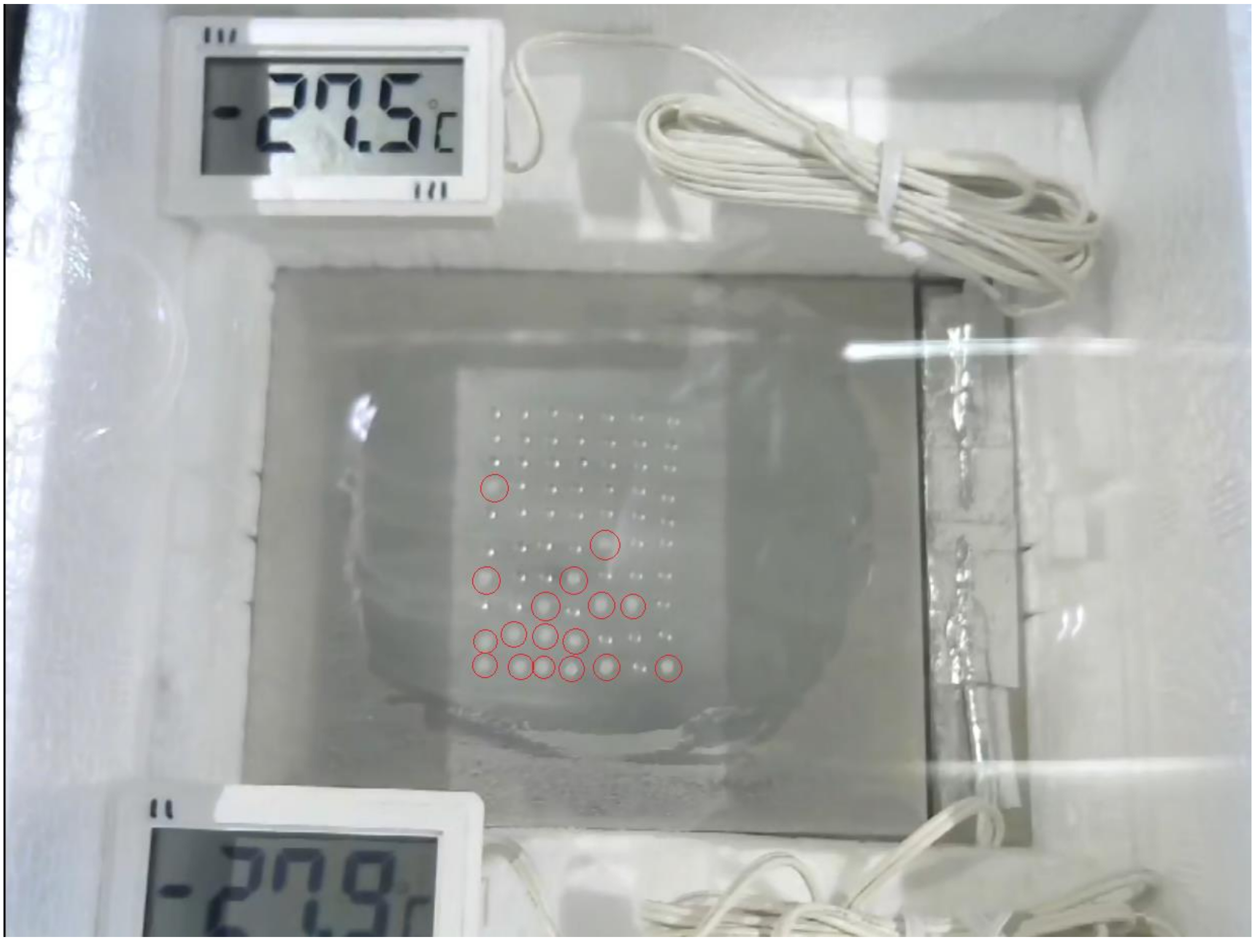
-27.5°C
-27.9°C

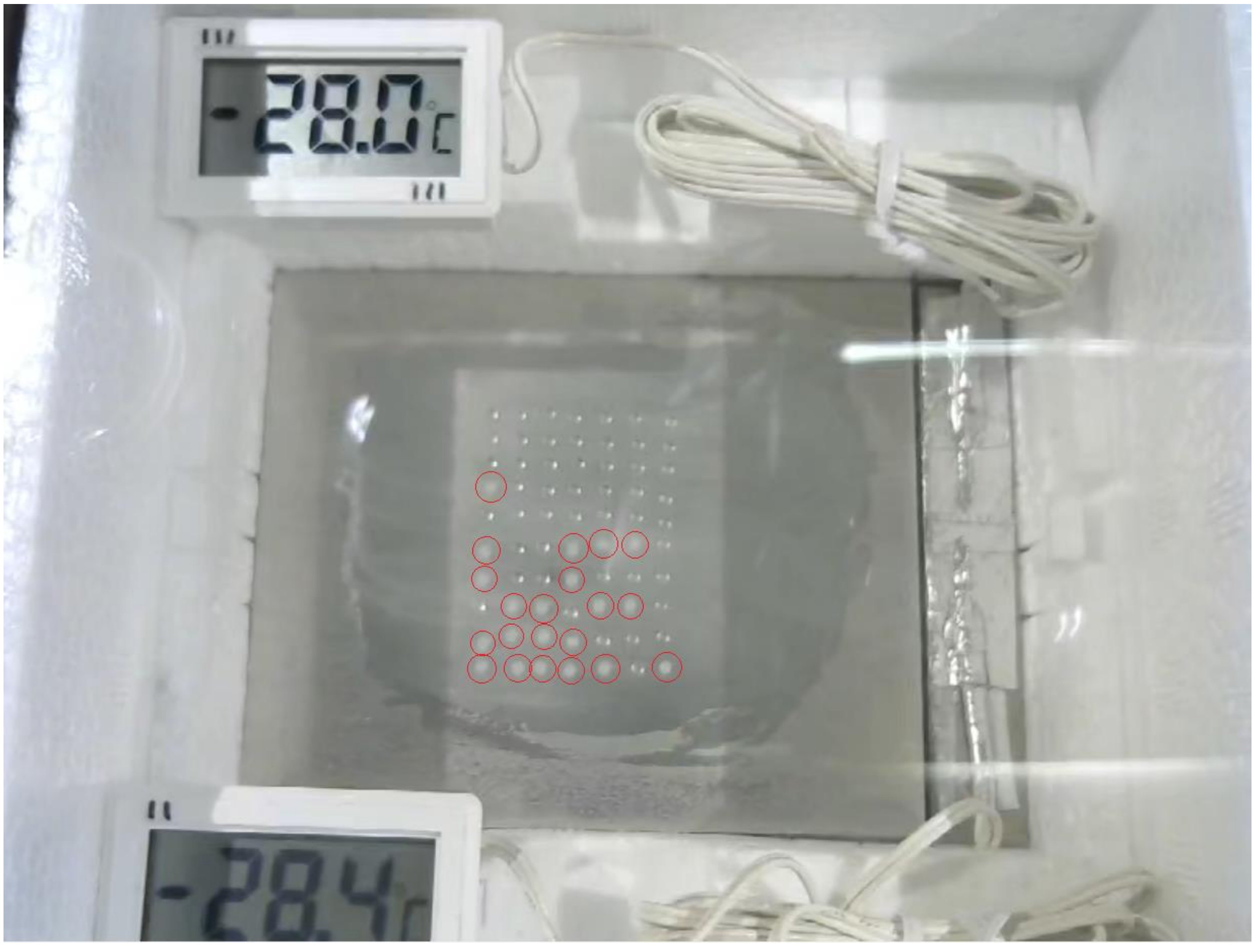

-28.0℃
-28.4℃

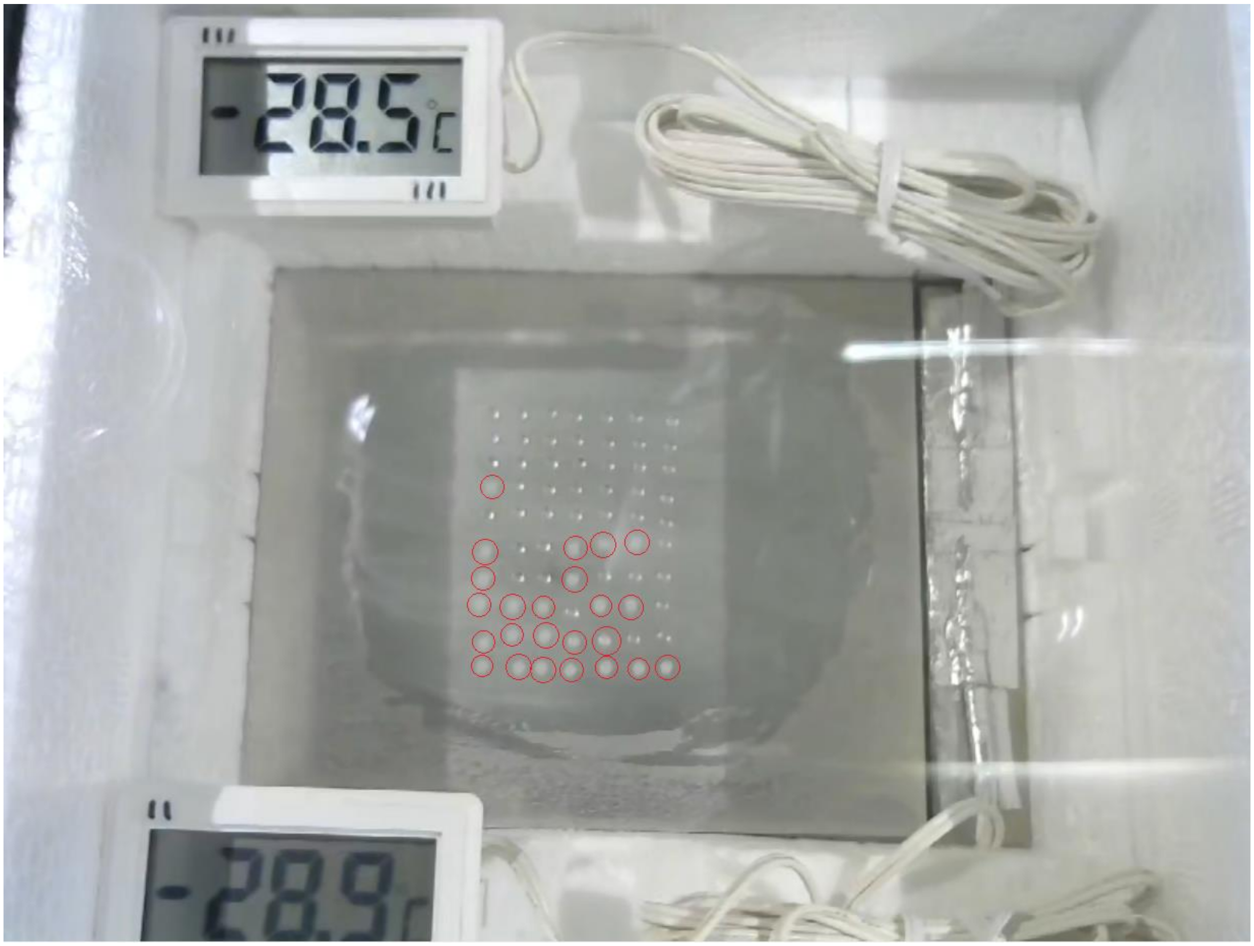
-28.5°C
-28.9°C

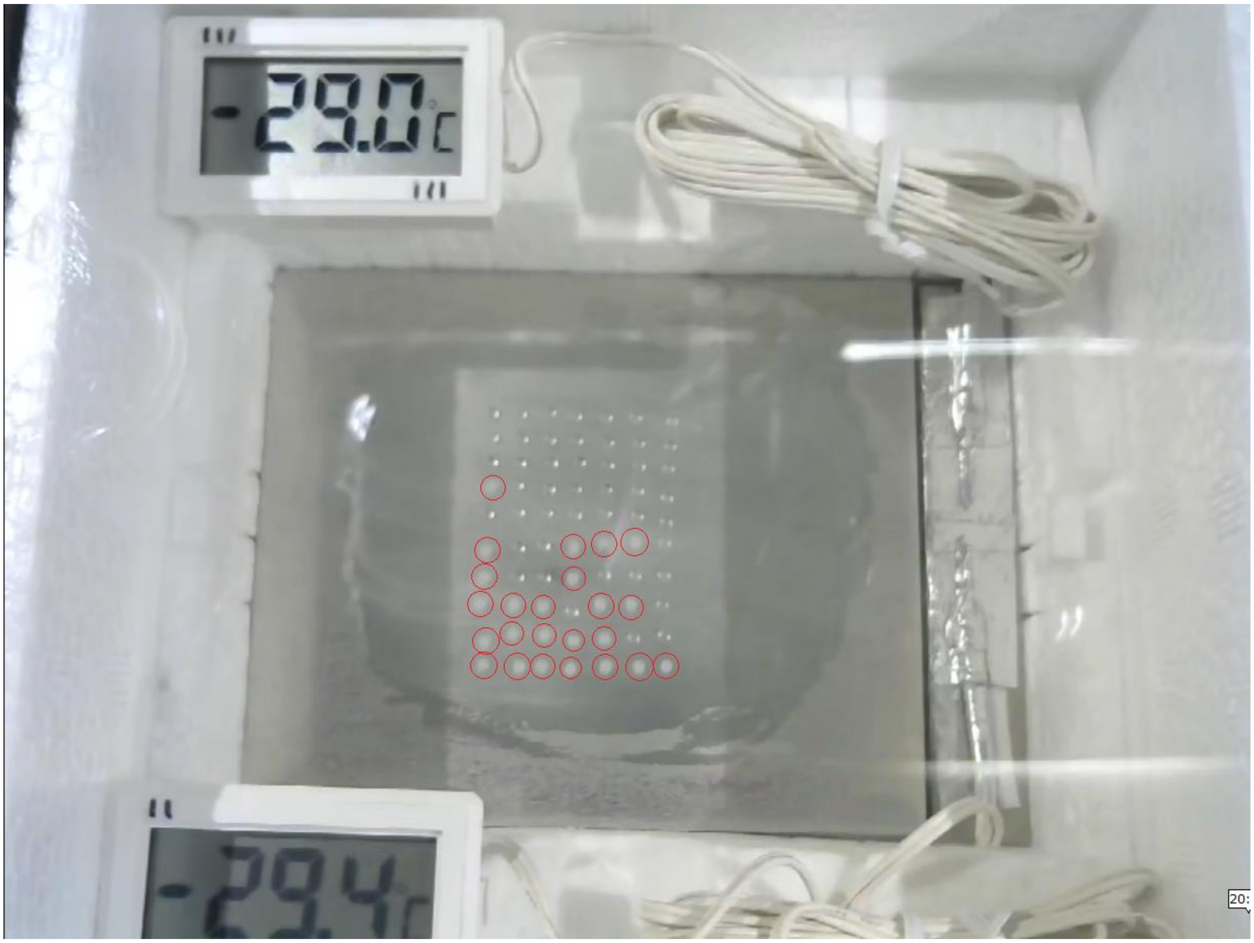
-29.0°C
-29.4°C

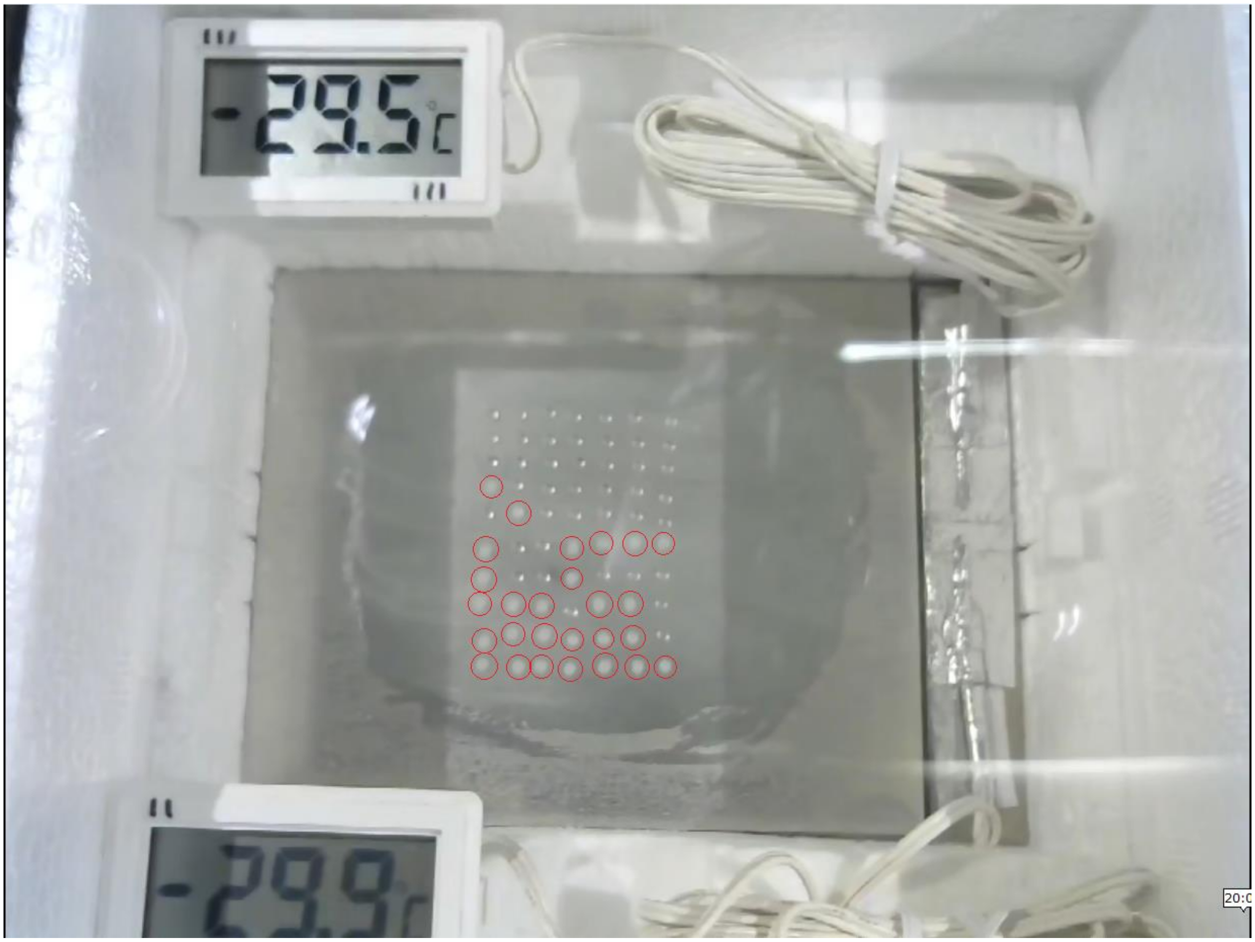
-29.5 C
-29.9 C

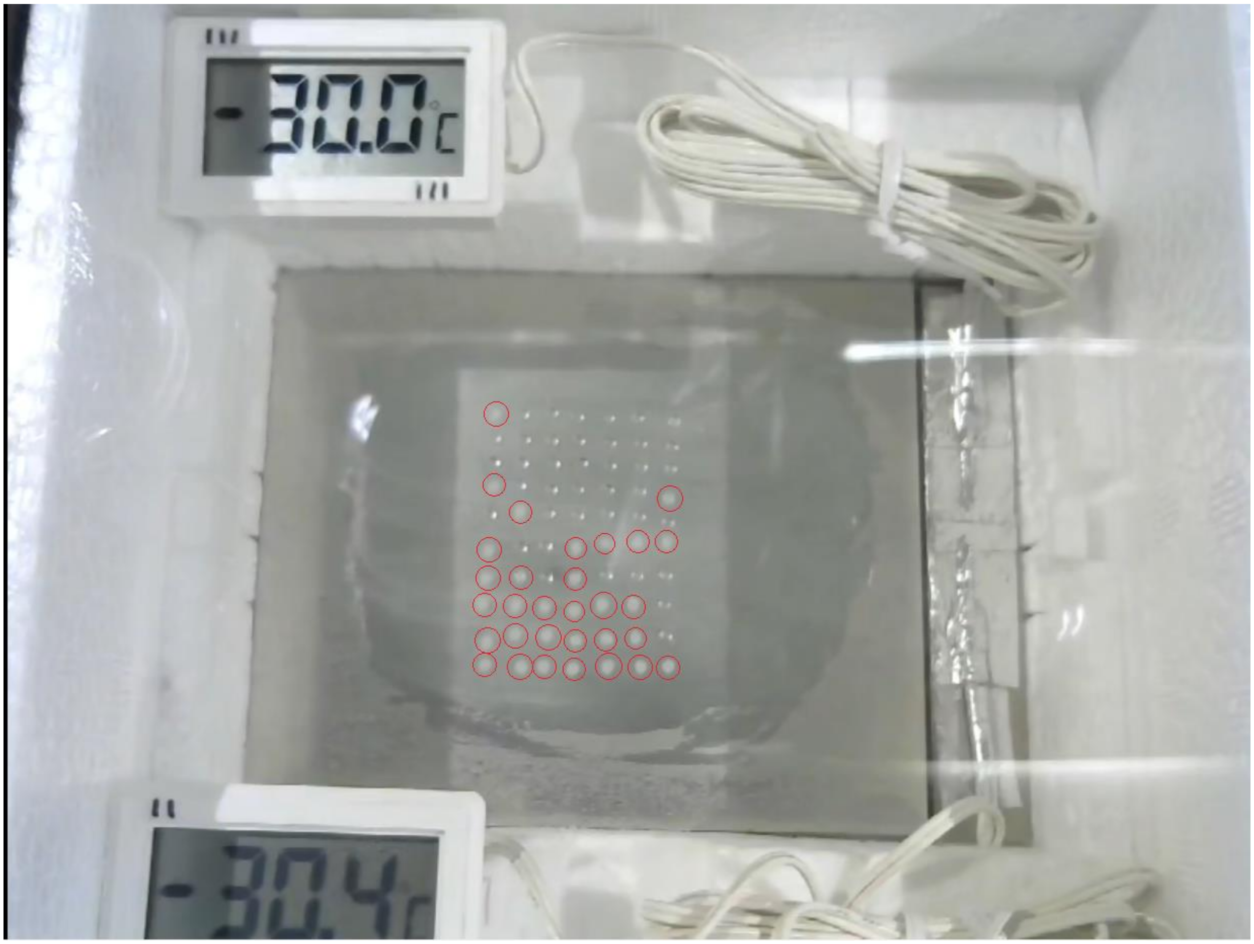
-30.0°C
-30.4°C

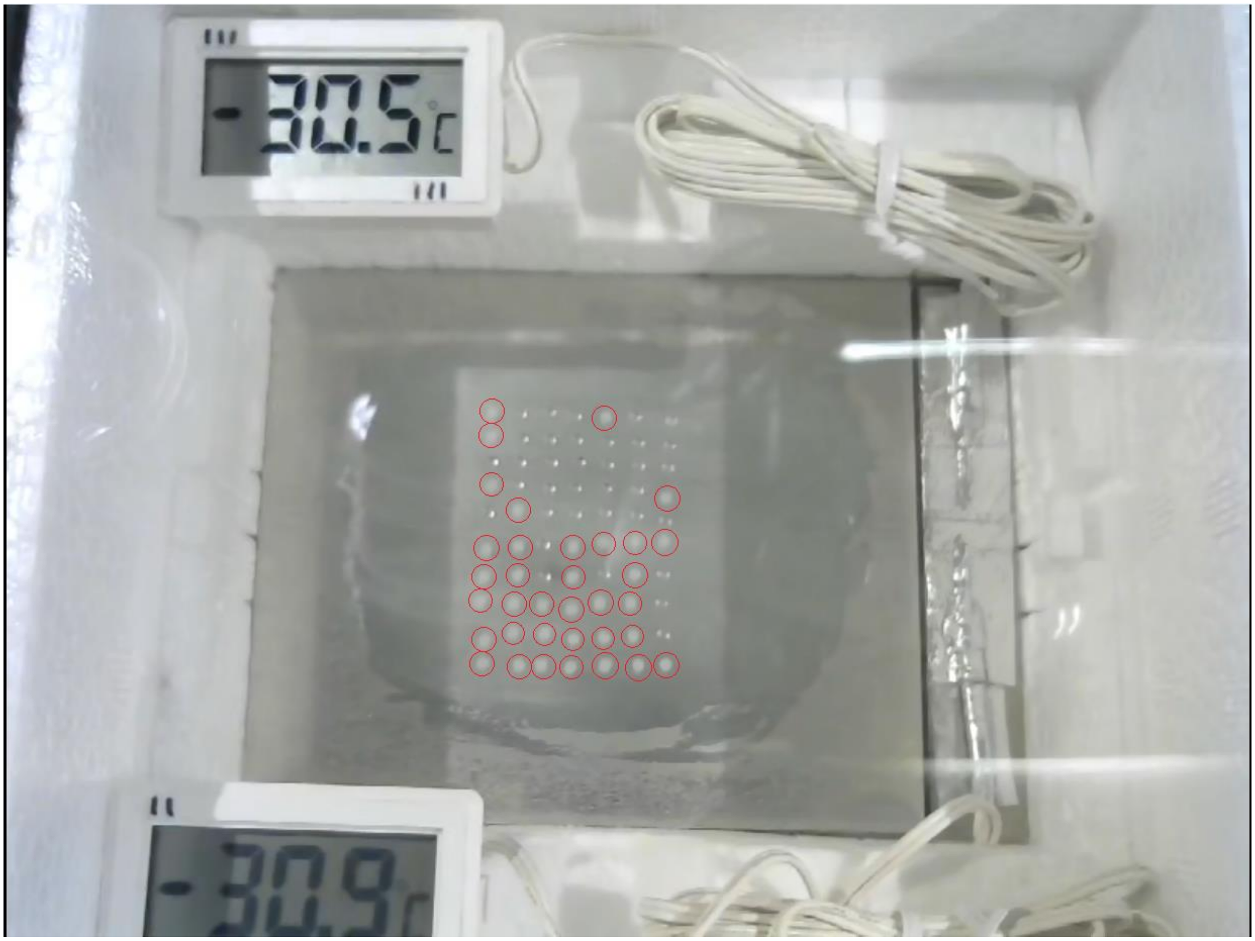
-30.5°C
-30.9°C

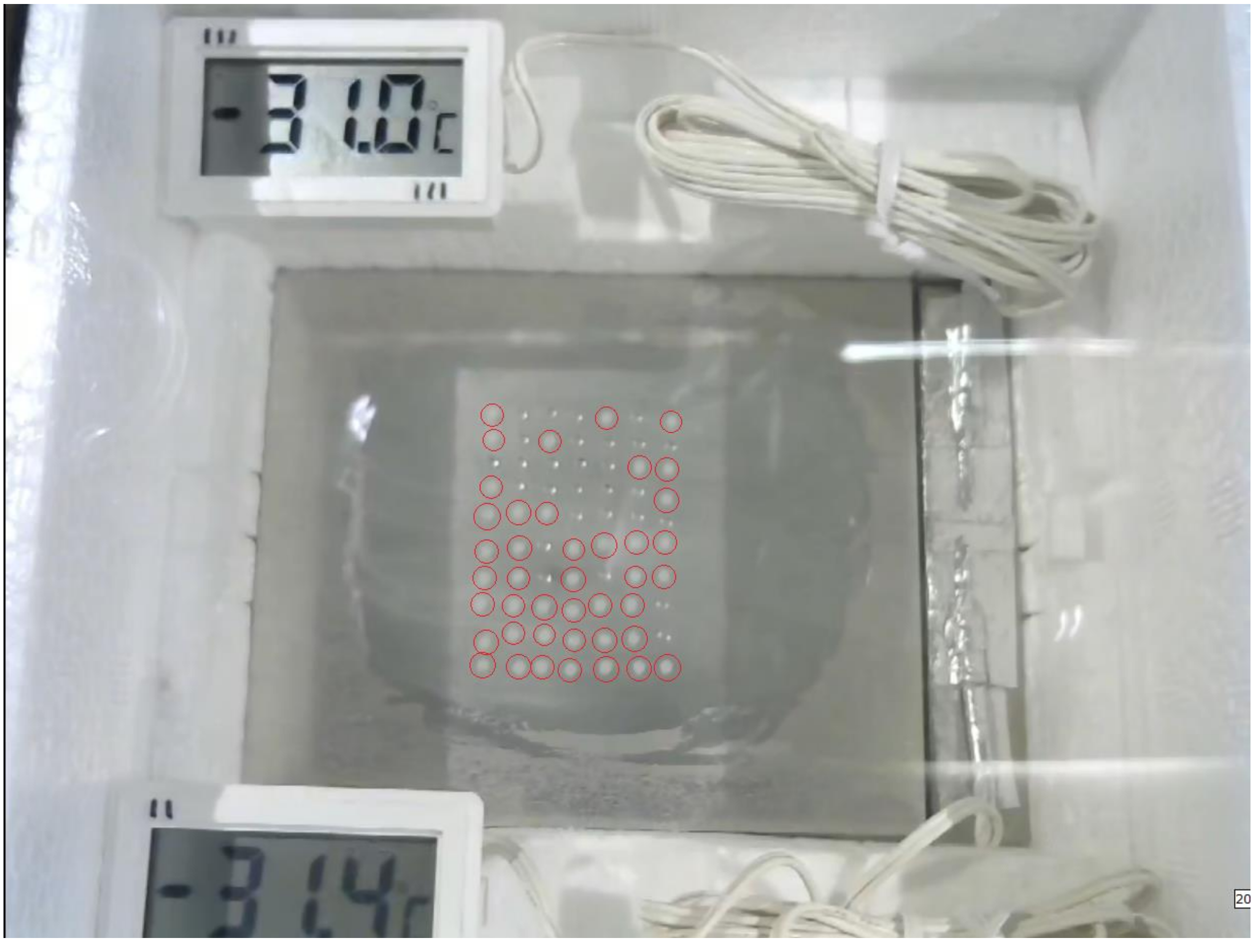
-31.0C
-31.4C

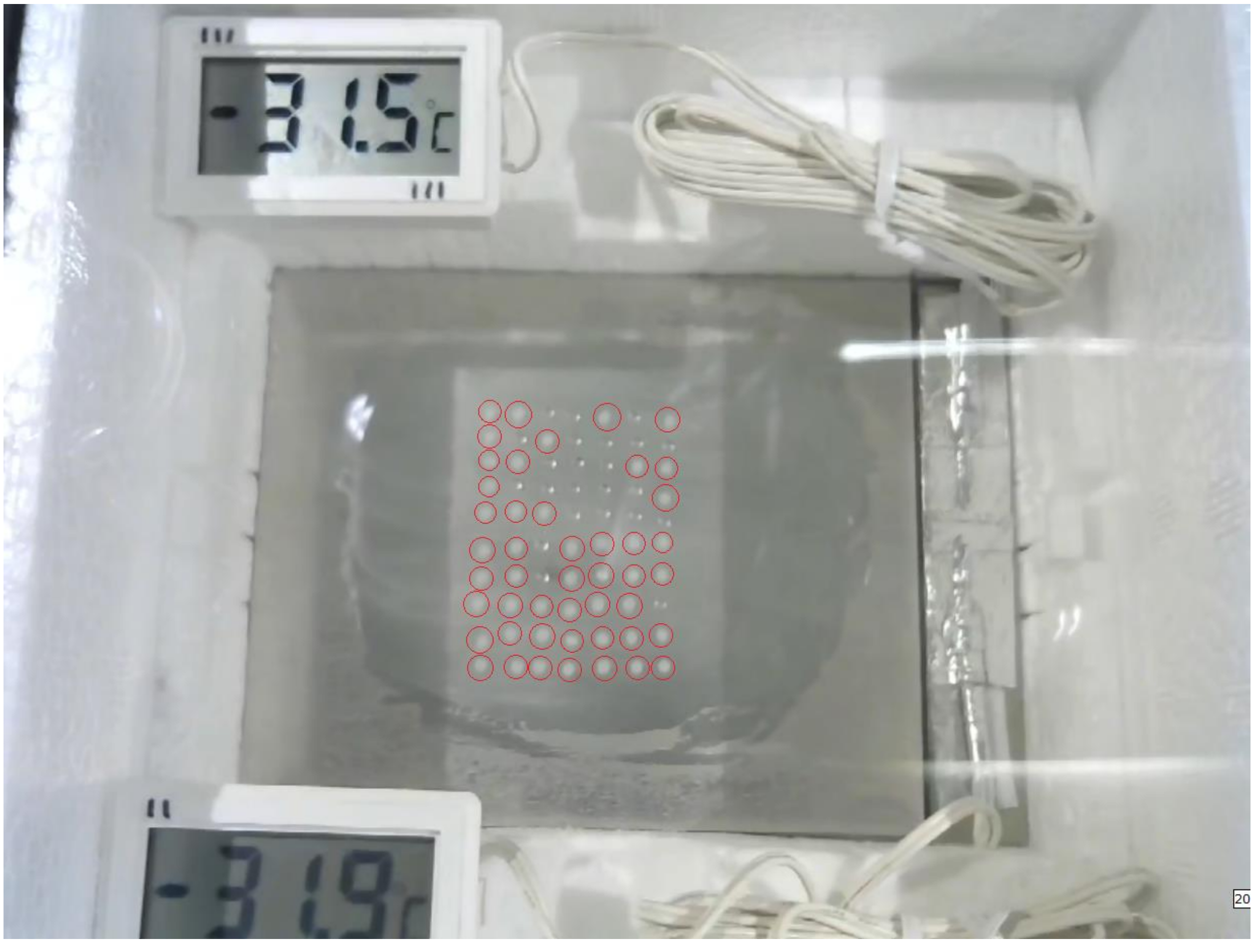
-31.5°C
-31.9

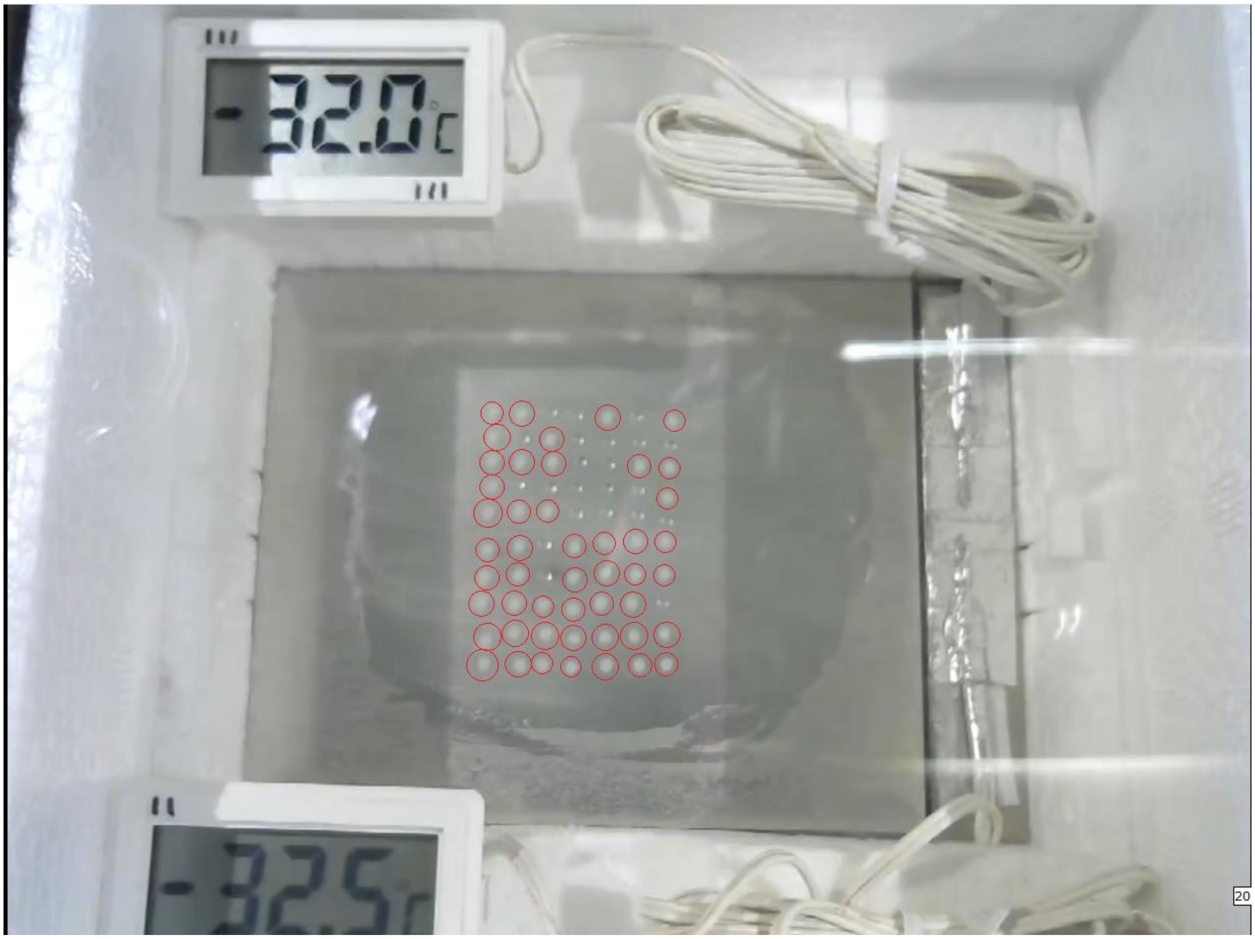
-32.0
-32.5
20

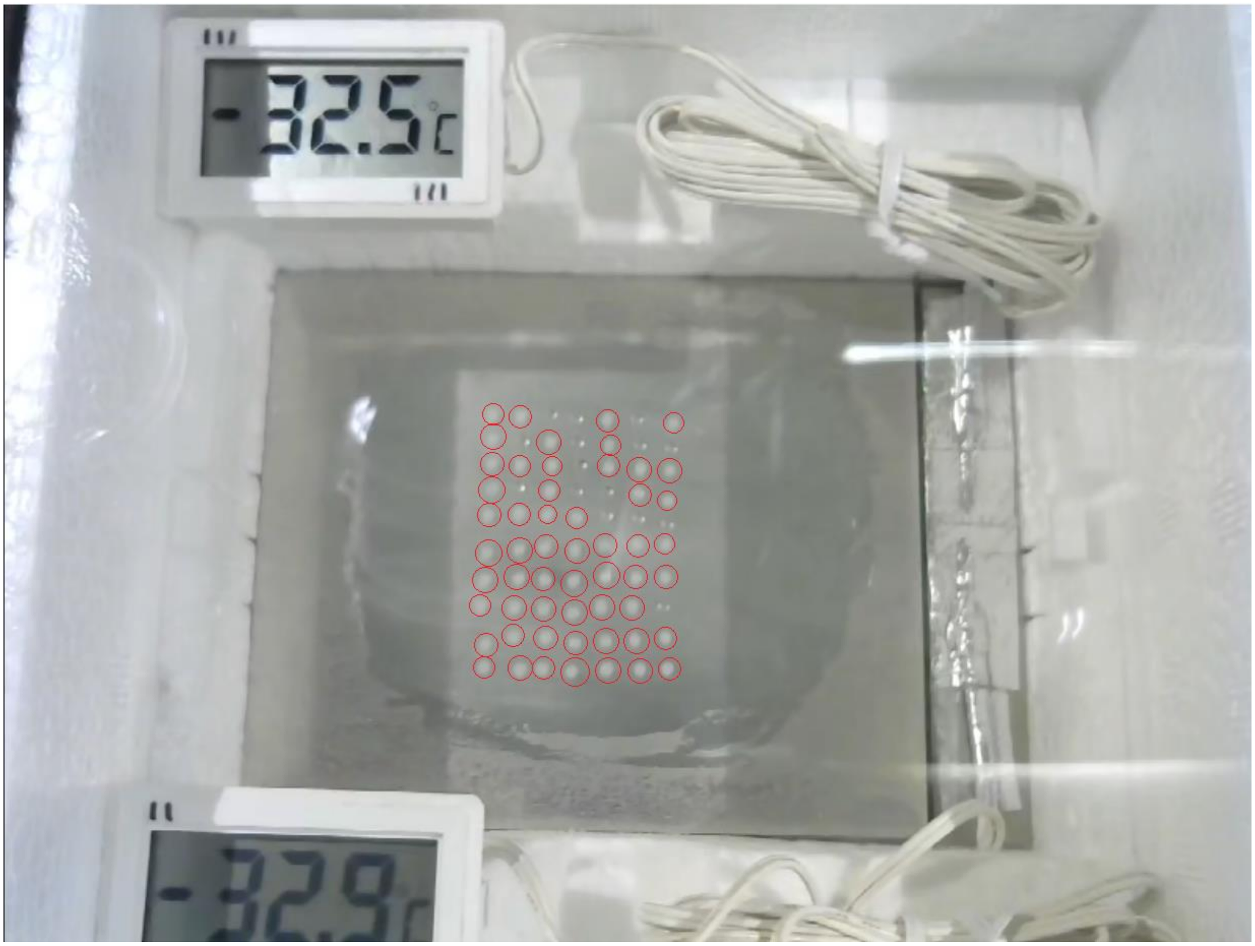
-32.5°C
-32.9°C

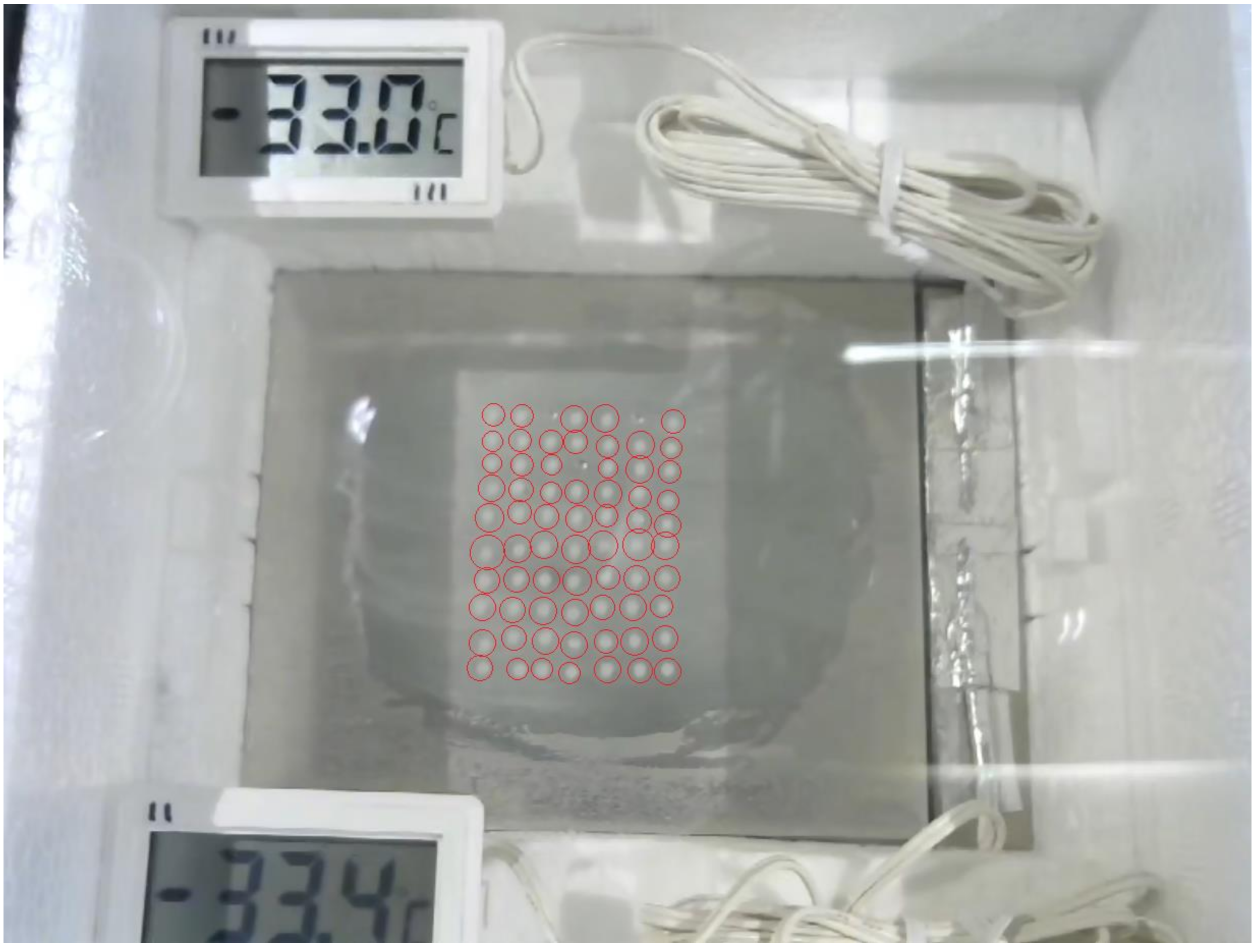
-33.0°C
-33.4°C

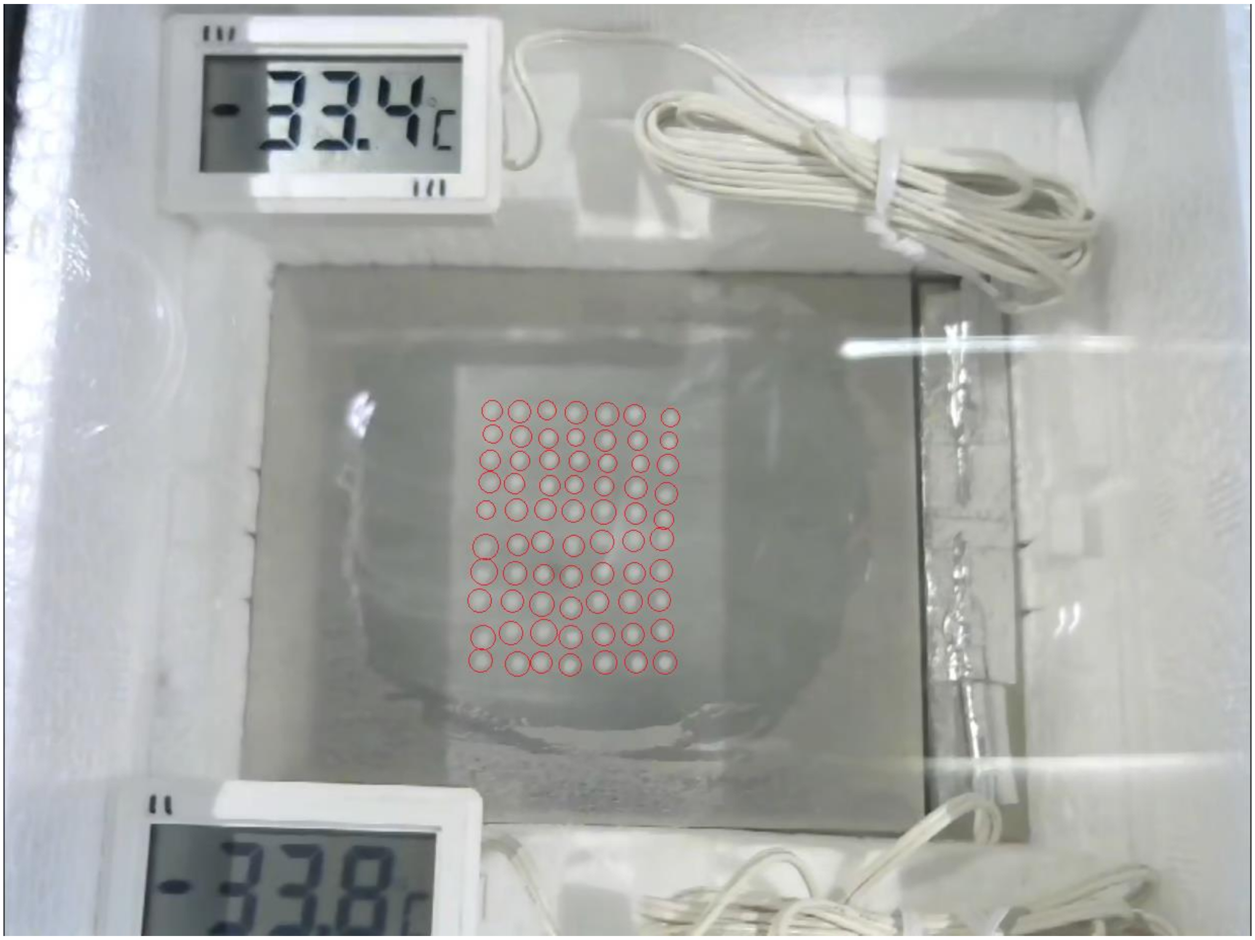
-33.4°C
-33.8°C
