# Supplementary material for: Integrated Science Teaching in Atmospheric Ice Nucleation Research: Immersion Freezing Experiments
Source: J Chem Educ. 2023 Mar 8;100(4):1511–22. doi: 10.1021/acs.jchemed.2c01060 (PMC10100551; doi:10.1021/acs.jchemed.2c01060)
Supplement: Supplementary file 1 — ed2c01060_si_001.zip [file ed2c01060_si_001.zip › SI_Files/Supplemental Information.docx]

**Supporting Information:**

**Integrated Science Teaching in Atmospheric Ice Nucleation Research:**

**Immersion Freezing Experiments**

Elise K. Wilbourn^1,♦^, Sarah Alrimaly^1,♦^, Holly Williams^1^, Jacob Hurst^2^, Gregory P. McGovern^2^,

Todd A. Anderson^3^, and Naruki Hiranuma^1,^*

^1^Dept. of Life, Earth, and Environmental Sciences, West Texas A&M University, Canyon, TX, 79016

^2^ Dept. of Chemistry and Physics, West Texas A&M University, Canyon, TX, 79016

^3^ Dept. of Environmental Toxicology, Texas Tech University, Lubbock, TX, 79416

^♦^These authors equally lead and contributed to this work

*Corresponding author (nhiranuma@wtamu.edu)

# **Supplemental Information (SI)**

# **Table of Contents**

Section Subject Page

S1 Module Instructions 2

Module_1_BULK_PROPERTIES

Module_2_WT_CRAFT

Module_3_SEM_EDX

S2 Module Assessment 2

1. Formal module assessment

2. Direct feedback

3. Remark & outlook

S3 Surface Tension Estimation 2

S4 Water Freezing Test Data 3

S5 Freezing Assay Calibration Procedure 4

S6 Advancing Freezing Assay 8

S7 Data Used to Generate Figures 8

References 9

**SI Sect. S1:** Module Instructions

To develop intuitive and student-centered curricular activities, a written protocol of the developed modules was documented as instructional materials. The DOCX copy of each module instruction is available in the **SI folder** (*SI_Sect_S1_Module_1_BULK_PROPERTIES.docx*, *SI_Sect_S1_Module_2_WT_CRAFT.docx*, and *SI_Sect_S1_Module_3_SEM_EDX.docx*).

**SI Sect. S2:** Module Assessment

The DOCX copy of the assessment summary, entitled *Module_Assessment*, is available in the **SI folder** (*SI_Sect_S2_Module_Assessment.docx*). The assessments of each module, associated students’ feedback in the classroom setting, and remark & outlook are discussed and provided in **Sect. 1**, **Sect. 2**, and **Sect. 3**, respectively.

**SI Sect. S3:** Surface Tension Estimation

Estimation of surface tension of bulk suspension sample is given by the Equation S1 as follows:

$y= \frac{1}{2}\cdot h\cdot r\cdot d\cdot g$ [S1]

y = surface tension (dybes cm^-1^)

h = distance between menisci (cm)

r = radius of capillary (= 0.025 cm)

d = density of sample at measuring temperature (≈ 0.998 g cm^-3^)

g = acceleration due to gravity at the location of measurement (≈ 980.7 cm s^-2^)

**SI Sect. S4:** Water Freezing Test Data

**Table S1** summarizes the West Texas Cryogenic Refrigerator Applied to Freezing Test (WT-CRAFT) calibration data based on measurements with filtered tap (F_Tap), deionized (DI), and high-performance liquid chromatography (HPLC)-grade water. Each measurement was performed by different individuals. The number of frozen droplets is shown as a function of temperature. The total number of droplets examined in each experiment was 70. Recorded videos are available through https://doi.pangaea.de/10.1594/PANGAEA.952536.

**Table S1.** A summary of WT-CRAFT calibration data with the examined water samples.

| T, °C | 33_F_Tap | *2a_DI | *3a_DI | 27_DI | 17_HPLC | 18_HPLC | 30_HPLC | 31_HPLC | 32_HPLC | **HPLC Average  ±  Standard Error | | |
| --- | --- | --- | --- | --- | --- | --- | --- | --- | --- | --- | --- | --- |
| 0 | 0 | 0 | 0 | 0 | 0 | 0 | 0 | 0 | 0 | 0.0 | ± | 0.0 |
| -0.5 | 0 | 0 | 0 | 0 | 0 | 0 | 0 | 0 | 0 | 0.0 | ± | 0.0 |
| -1 | 0 | 0 | 0 | 0 | 0 | 0 | 0 | 0 | 0 | 0.0 | ± | 0.0 |
| -1.5 | 0 | 0 | 0 | 0 | 0 | 0 | 0 | 0 | 0 | 0.0 | ± | 0.0 |
| -2 | 0 | 0 | 0 | 0 | 0 | 0 | 0 | 0 | 0 | 0.0 | ± | 0.0 |
| -2.5 | 0 | 0 | 0 | 0 | 0 | 0 | 0 | 0 | 0 | 0.0 | ± | 0.0 |
| -3 | 0 | 0 | 0 | 0 | 0 | 0 | 0 | 0 | 0 | 0.0 | ± | 0.0 |
| -3.5 | 0 | 0 | 0 | 0 | 0 | 0 | 0 | 0 | 0 | 0.0 | ± | 0.0 |
| -4 | 0 | 0 | 0 | 0 | 0 | 0 | 0 | 0 | 0 | 0.0 | ± | 0.0 |
| -4.5 | 0 | 0 | 0 | 0 | 0 | 0 | 0 | 0 | 0 | 0.0 | ± | 0.0 |
| -5 | 0 | 0 | 0 | 0 | 0 | 0 | 0 | 0 | 0 | 0.0 | ± | 0.0 |
| -5.5 | 0 | 0 | 0 | 0 | 0 | 0 | 0 | 0 | 0 | 0.0 | ± | 0.0 |
| -6 | 0 | 0 | 0 | 0 | 0 | 0 | 0 | 0 | 0 | 0.0 | ± | 0.0 |
| -6.5 | 0 | 0 | 0 | 0 | 0 | 0 | 0 | 0 | 0 | 0.0 | ± | 0.0 |
| -7 | 0 | 0 | 0 | 0 | 0 | 0 | 0 | 0 | 0 | 0.0 | ± | 0.0 |
| -7.5 | 0 | 0 | 0 | 0 | 0 | 0 | 0 | 0 | 0 | 0.0 | ± | 0.0 |
| -8 | 0 | 0 | 0 | 0 | 0 | 0 | 0 | 0 | 0 | 0.0 | ± | 0.0 |
| -8.5 | 0 | 0 | 0 | 0 | 0 | 0 | 0 | 0 | 0 | 0.0 | ± | 0.0 |
| -9 | 0 | 0 | 0 | 0 | 0 | 0 | 0 | 0 | 0 | 0.0 | ± | 0.0 |
| -9.5 | 0 | 0 | 0 | 0 | 0 | 0 | 0 | 0 | 0 | 0.0 | ± | 0.0 |
| -10 | 0 | 0 | 0 | 0 | 0 | 0 | 0 | 0 | 0 | 0.0 | ± | 0.0 |
| -10.5 | 0 | 0 | 0 | 0 | 0 | 0 | 0 | 0 | 0 | 0.0 | ± | 0.0 |
| -11 | 0 | 0 | 0 | 0 | 0 | 0 | 0 | 0 | 0 | 0.0 | ± | 0.0 |
| -11.5 | 0 | 0 | 0 | 0 | 0 | 0 | 0 | 0 | 0 | 0.0 | ± | 0.0 |
| -12 | 0 | 0 | 0 | 0 | 0 | 0 | 0 | 0 | 0 | 0.0 | ± | 0.0 |
| -12.5 | 0 | 0 | 0 | 0 | 0 | 0 | 0 | 0 | 0 | 0.0 | ± | 0.0 |
| -13 | 0 | 0 | 0 | 0 | 0 | 0 | 0 | 0 | 0 | 0.0 | ± | 0.0 |
| -13.5 | 0 | 0 | 0 | 0 | 0 | 0 | 0 | 0 | 0 | 0.0 | ± | 0.0 |
| -14 | 0 | 0 | 0 | 0 | 0 | 0 | 0 | 0 | 0 | 0.0 | ± | 0.0 |
| -14.5 | 0 | 0 | 0 | 0 | 0 | 0 | 0 | 0 | 0 | 0.0 | ± | 0.0 |
| -15 | 1 | 0 | 0 | 0 | 0 | 0 | 0 | 0 | 0 | 0.0 | ± | 0.0 |
| -15.5 | 3 | 0 | 0 | 0 | 0 | 0 | 0 | 0 | 1 | 0.2 | ± | 0.2 |
| -16 | 4 | 0 | 0 | 0 | 0 | 0 | 0 | 0 | 1 | 0.2 | ± | 0.2 |
| -16.5 | 5 | 0 | 1 | 0 | 0 | 0 | 0 | 0 | 1 | 0.2 | ± | 0.2 |
| -17 | 5 | 0 | 1 | 0 | 0 | 0 | 0 | 0 | 1 | 0.2 | ± | 0.2 |
| -17.5 | 6 | 0 | 1 | 0 | 0 | 0 | 0 | 0 | 1 | 0.2 | ± | 0.2 |
| -18 | 7 | 0 | 1 | 0 | 0 | 0 | 0 | 0 | 1 | 0.2 | ± | 0.2 |
| -18.5 | 10 | 0 | 1 | 0 | 1 | 0 | 0 | 0 | 1 | 0.4 | ± | 0.2 |
| -19 | 13 | 1 | 2 | 0 | 1 | 0 | 0 | 0 | 1 | 0.4 | ± | 0.2 |
| -19.5 | 16 | 1 | 2 | 0 | 1 | 0 | 0 | 0 | 1 | 0.4 | ± | 0.2 |
| -20 | 21 | 1 | 3 | 0 | 2 | 0 | 0 | 0 | 1 | 0.6 | ± | 0.4 |
| -20.5 | 24 | 1 | 4 | 0 | 2 | 0 | 0 | 0 | 1 | 0.6 | ± | 0.4 |
| -21 | 27 | 1 | 5 | 0 | 2 | 0 | 0 | 0 | 1 | 0.6 | ± | 0.4 |
| -21.5 | 36 | 1 | 5 | 2 | 2 | 0 | 0 | 0 | 1 | 0.6 | ± | 0.4 |
| -22 | 37 | 1 | 6 | 2 | 2 | 1 | 0 | 0 | 1 | 0.8 | ± | 0.4 |
| -22.5 | 42 | 2 | 6 | 4 | 2 | 1 | 0 | 0 | 1 | 0.8 | ± | 0.4 |
| -23 | 54 | 2 | 7 | 5 | 2 | 1 | 0 | 0 | 1 | 0.8 | ± | 0.4 |
| -23.5 | 60 | 3 | 7 | 5 | 2 | 1 | 0 | 0 | 1 | 0.8 | ± | 0.4 |
| -24 | 68 | 4 | 7 | 7 | 3 | 1 | 0 | 0 | 1 | 1.0 | ± | 0.5 |
| -24.5 | 70 | 4 | 7 | 9 | 3 | 2 | 0 | 0 | 1 | 1.2 | ± | 0.6 |
| -25 | 70 | 4 | 8 | 10 | 4 | 2 | 0 | 1 | 1 | 1.6 | ± | 0.7 |
| -25.5 | N/A | 4 | 8 | 10 | 5 | 2 | 0 | 1 | 1 | 1.8 | ± | 0.9 |
| -26 | N/A | 6 | 8 | 13 | 5 | 2 | 0 | 1 | 1 | 1.8 | ± | 0.9 |
| -26.5 | N/A | 7 | 8 | 22 | 5 | 2 | 0 | 1 | 1 | 1.8 | ± | 0.9 |
| -27 | N/A | 11 | 8 | 46 | 5 | 2 | 0 | 1 | 1 | 1.8 | ± | 0.9 |
| -27.5 | N/A | 17 | 10 | 57 | 6 | 2 | 1 | 1 | 1 | 2.2 | ± | 1.0 |
| -28 | N/A | 21 | 17 | 66 | 7 | 2 | 1 | 1 | 2 | 2.6 | ± | 1.1 |
| -28.5 | N/A | 24 | 24 | 69 | 8 | 4 | 1 | 2 | 2 | 3.4 | ± | 1.2 |
| -29 | N/A | 24 | 36 | 69 | 10 | 8 | 1 | 2 | 2 | 4.6 | ± | 1.8 |
| -29.5 | N/A | 27 | 46 | 69 | 10 | 11 | 1 | 3 | 4 | 5.8 | ± | 2.0 |
| -30 | N/A | 31 | 55 | 69 | 10 | 11 | 1 | 6 | 4 | 6.4 | ± | 1.9 |
| -30.5 | N/A | 35 | 58 | N/A | 11 | N/A | 3 | 6 | 4 | 6.0 | ± | 1.6 |
| -31 | N/A | 42 | 61 | N/A | 11 | N/A | 4 | 6 | 6 | 6.8 | ± | 1.3 |
| -31.5 | N/A | 47 | 61 | N/A | 11 | N/A | 4 | 6 | 6 | 6.8 | ± | 1.3 |
| -32 | N/A | 48 | 61 | N/A | 12 | N/A | 5 | 7 | 6 | 7.5 | ± | 1.4 |
| -32.5 | N/A | 55 | 61 | N/A | 14 | N/A | 6 | 9 | 6 | 8.8 | ± | 1.7 |
| -33 | N/A | 67 | 62 | N/A | 15 | N/A | 7 | 10 | 8 | 10.0 | ± | 1.6 |
| -33.5 | N/A | 70 | 69 | N/A | 23 | N/A | 32 | 30 | 23 | 27.0 | ± | 2.1 |
| -34 | N/A | N/A | N/A | N/A | 41 | N/A | 69 | 62 | 62 | 58.5 | ± | 5.4 |
| -34.5 | N/A | N/A | N/A | N/A | 70 | N/A | 70 | 69 | 70 | 69.8 | ± | 0.2 |
| -35 | N/A | N/A | N/A | N/A | N/A | N/A | 70 | 69 | 70 | 69.7 | ± | 0.3 |
| -35.5 | N/A | N/A | N/A | N/A | N/A | N/A | N/A | 69 | 70 | 69.5 | ± | 0.3 |
| -36 | N/A | N/A | N/A | N/A | N/A | N/A | N/A | 69 | 70 | 69.5 | ± | 0.3 |

*We tested the systematic variation of the temperature sensor by co-deploying two identical sensors on the aluminum substrate and simultaneously measuring the substrate temperature. As seen in the video (2a_DI and 3a_DI), the largest deviation between two sensors is 0.5 °C, which is reported as the systematic uncertainty in the WT-CRAFT temperature (Vepuri et al., 2021).; **We consider the first 3% freezing events (~2/70 droplets) stem from uncontrollable artifacts as reported in Table S4 of Hiranuma et al. (2019).

**SI Sect. S5:** Freezing Assay Calibration Procedure

This section describes the procedure used for the WT-CRAFT calibration with known ice-nucleating particle (INP) calibrators. A recent method intercomparison study shows reasonable agreement between WT-CRAFT and other immersion freezing techniques for ultrapure waters as well as the known composition of commercially available materials (e.g., microcrystalline cellulose, MCC) within the known uncertainties (Hiranuma et al., 2019). While there is no stable calibrator used within the field as an INP standard to the best of our knowledge, two other materials, including Snomax (Wex et al., 2015) and illite NX (Hiranuma et al., 2015), have been widely used as calibrator-surrogates in the INP research community. The instructing team performed the system calibration using these INPs before exposing the students to the WT-CRAFT module lesson. Below, the (A) calibration protocol and (B) results are presented.

*(A) Procedure:* Based on Vali (1971) and Eqns. 3 and 4 of Hiranuma (2015), we describe the derivations of INP concentration per unit mass, *n*_m_, as well as INP concentration per unit geometric aerosol particle surface, *n*_s,geo_, as proxies of immersion freezing efficiencies**.** First, the *C*_INP_(*T*) value can be computed to represent the nucleus concentration in suspension (L*^−^*^1^ water) as a function of temperature as:

$$C_{INP}\left( T \right)= - \frac{\ln\left( 1-FF\left( T \right) \right)}{V_{d}} [S2]$$

in which, *V_d_* is the volume of the sample in each droplet (3 *µ*L or 0.003 mL) for WT-CRAFT. This equation is identical to Eqn. 9 in the module lesson plan and experimentation material (refer to Module 2 in **SI Sect. S1**). Following this initial calculation, *C_INP_*(*T*) can be subsequently converted to *n*_m_(*T*) as:

$$n_{m}\left( T \right)= \frac{C_{INP}\left( T \right)}{C_{m}}\times DF [S3]$$

where *C_m_* is a mass concentration of analyte per unit volume of suspension (mg mL^-1^), and *DF* is a serial dilution factor (i.e., x1 to x10^7^ for this study). Each suspension sample of test INPs was prepared by suspending dry powder in the HPLC water. Specific ranges of weight percent (wt%; e.g., 1 mg mL^-1^ = 0.1 wt%) were used and examined for each sample type (i.e., MCC 0.05-10^-4^ wt%, Snomax 0.1-10^-8^ wt%, and illite NX 0.1-10^-6^ wt%). Moreover, the serial dilution method discussed in Cory (2017) and Vepuri et al. (2021) was employed to assess immersion freezing efficiency in a wide range of temperatures (at least down to -30 °C) through WT-CRAFT. All suspension samples (including diluted suspensions) were prepared using disposable and sterilized tubes and pipette tips, which minimize the additional introduction of contaminants and potential INPs. This practical feedback was provided by the interns. Finally, *n*_s,geo_(*T*) can be derived using:

$$n_{s,geo}\left( T \right) \approx\frac{n_{m}\left( T \right)}{SSA} [S4]$$

where *SSA* is a geometric specific surface area (m^2^ g^-1^). The SSA values used for MCC, Snomax, and illite NX are 0.068 m^2^ g^-1^, 7.99 m^2^ g^-1^, and 6.54 m^2^ g^-1^, respectively (Hiranuma et al., 2019; Wex et al., 2015; Hiranuma et al., 2015).

This examination with known compositions (i.e., pure water, Snomax, MCC, and illite NX) provided a comprehensive and insightful opportunity for students to understand how to calibrate WT-CRFAT against non-biological and biological INP proxies.

*(B) Results:* After providing adequate training, intern students first ran background WT-CRAFT immersion freezing tests with HPLC water and DI water, which is purified water through an ion exchange cartridge to achieve a resistivity of 18.2 MΩ.cm at 25 °C. An example model result of the DI water freezing experiment is shown in **Fig. S1**. As can be seen in **Fig. S1**, students assessed the progression of freezing events by comparing images of examined droplets from unfrozen conditions (frozen fraction or *FF* = 0) to 100% frozen state (*FF* = 1) every 0.5 °C.

**Figure S2** shows the resultant *FF* spectra of 3 µL droplets from the tested water samples superposed on the reference homogeneous freezing spectrum (Koop and Murray, 2016). As seen, both HPLC and DI water types show similar freezing properties as a function of temperature, but intern students found DI water contains more INPs than HPLC water. The estimated *T_FF_*_50_ values were approximately -28 °C and -33.5 °C for DI water and HPLC water, respectively. More importantly, both *FF* spectra show a hump of water freezing above -33 °C and, thereby, a deviation from a homogeneous freezing spectrum was observed, especially at *FF* below ~0.5. The observed hump and deviation may stem from impurities in the water samples acting as INPs (detailed in **Sect. 3.3**) or could derive from artifacts during the preparation of the experiment. This emphasizes the importance of consistent laboratory protocol (**SI Sect. 1** Module 2) for minimizing and eliminating potential artifacts. Further details of the model experimental data for individual water samples and associated statistical uncertainties, as well as the video recorded during each experiment, are provided in **SI Sects. 4 and 7**, respectively. Nevertheless, the *FF* result with HPLC water is statistically reproducible within computed uncertainties (**SI Sect. 4**). Thus, the average *FF*(*T*) of our measurements with HPLC water is considered as our negative control (i.e., calibration standard) on WT-CRAFT, which is a practical reference for training students in the classroom setting.

Next, interns examined freezing properties of suspensions with known ice nucleation active compounds in immersion mode (i.e., illite NX, Snomax, and MCC) to ascertain whether previous laboratory results are reproducible on WT-CRAFT. **Figure S3** summarizes the immersion freezing efficiency data by means of *n*_s,geo_(*T*). The abovementioned SSA values were adapted for the conversion of *FF*(*T*) to *n*_s,geo_(*T*). As shown in **Fig. S3**, a negligible deviation was confirmed for the results from previous immersion freezing results. This assessment was carried out to establish positive controls with known water samples. Through this positive control test, students gained hands-on opportunity and insight to fully understand that INPs in suspension can act as ice-nucleating compounds in the examined samples, causing heterogeneous freezing. The removal of these compounds through the dilution procedure, described in Vepuri et al. (2021) has demonstrated that assessing heterogeneous freezing at -25 °C (with < 3% inclusion of artifacts) and lower temperatures (with the potential inclusion of artifacts) is possible when following this procedure.


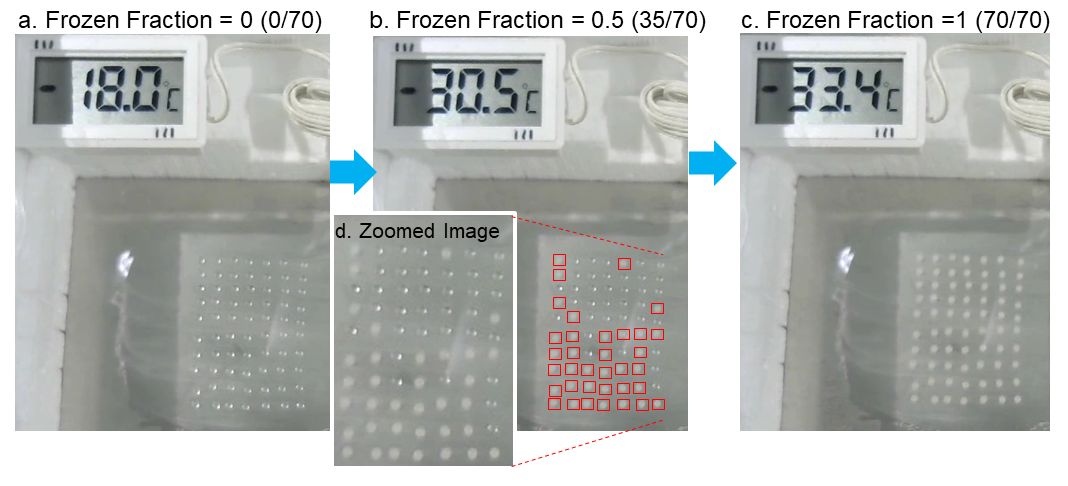


**Figure S1**. Progression of DI water droplet freezing on WT-CRAFT (Sample ID 2a_DI – see **SI Sect. 4**). The images shown in each panel correspond to FF of 0 (a), 0.5 (b), and 1 (c). Frozen droplets in Panel b are indicated by red squares. Panel d is a magnified image of Panel b without any identification markers.


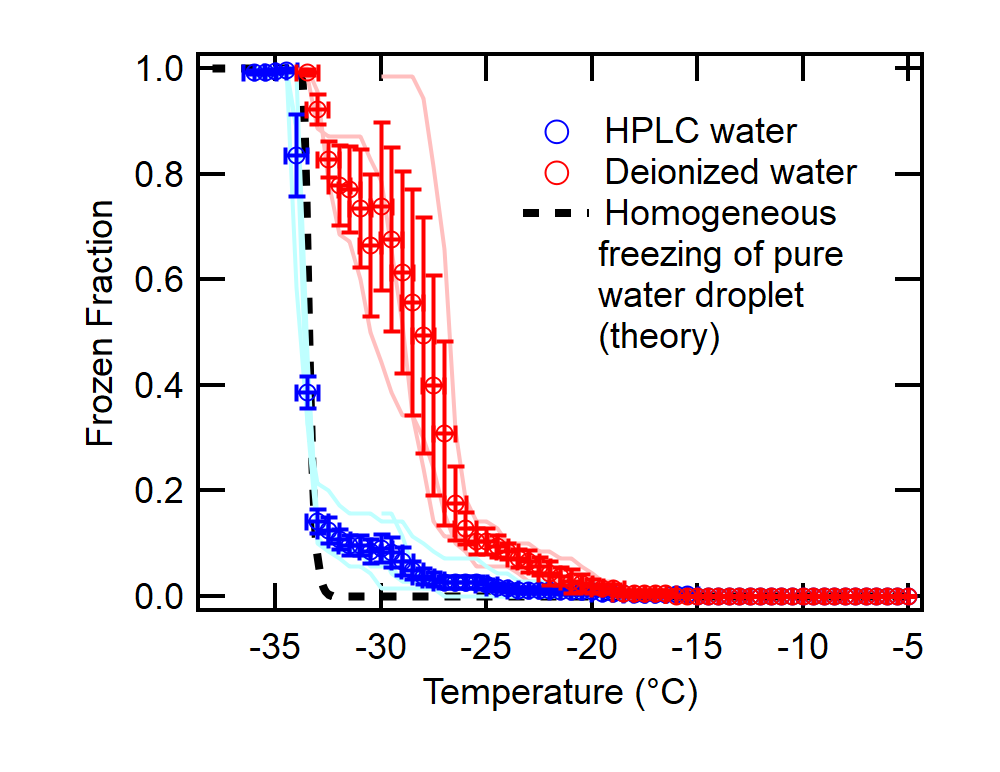


**Figure S2.** Frozen fraction for DI water (red circle, an average of N = 3), HPLC water (blue circle, an average of N = 5), and theoretical homogeneous droplet freezing (dashed grey). Horizontal error bars represent the temperature uncertainty of WT-CRAFT (± 0.5 °C). Light pink and blue lines are individual measurements of DI water and HPLC water, respectively (original data are reported in **SI Sect. 4**), and the vertical error bars are standard errors.


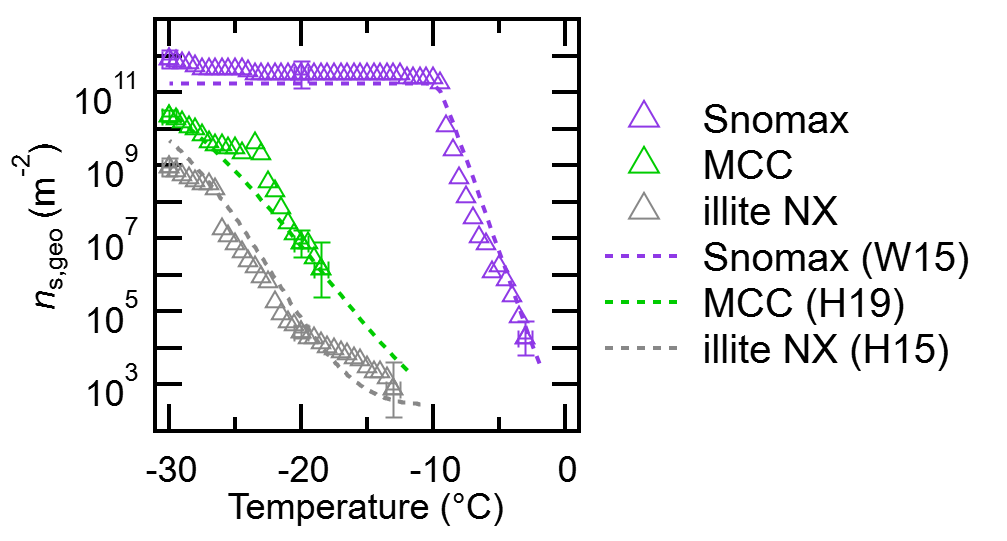


**Figure S3.** Experimental vs. reference *n*_s,geo_ for Snomax, MCC, and illite NX. The error values are shown for a subset of data points. W15, H19, and H15 denote Wex et al. (2015), Hiranuma et al. (2019), and Hiranuma et al. (2015), respectively. The temperature uncertainty (± 0.5 °C) and *n*_s,geo_ error [± CI95% described in Schiebel (2017)] are shown for the selected data (first freezing event observed at the highest temperature, -20 °C, and -30 °C) to keep all data points visible.

**SI Sect. S6:** Advancing Freezing Assay

This section discusses how to advance freezing assay. A small concentration of hydrogen peroxide, H_2_O_2_, oxidatively destroys the ice nucleation ability of organic impurities in suspension samples (Hill et al., 2016; Perkins et al., 2020). The observed suppression in organic INP ability is presumably attributed to chemical oxidative modification or adsorption to the active site. To improve the detection sensitivity of WT-CRAFT (plus other freezing assays) and remove water impurities with respect to immersion freezing, it is plausible to utilize oxidating reactions, in which aqueous H_2_O_2_ undergoes when exposed to heat and ultraviolet light (Lunak and Sedlak, 1992). This treatment might be especially useful to purify test water by removing organic impurities. The removal of these compounds, which were not detectable in offline analysis (**Sect. 3.3**), through chemical treatment, might realize homogeneous freezing (**Fig. S2**) when following this procedure.

The solute effect of H_2_O_2_ (< 20 µL) on homogeneous freezing, equilibrium melting temperature, and water activity has been previously studied, and it was observed that the effect is negligible (Oguni and Angell, 1980; Koop et al., 2000). Thus, H_2_O_2_ seems normal in terms of water activity compared to homogeneously freezing water.

Some H_2_O_2_ products contain stabilizers to prevent H_2_O_2_ degradation. These stabilizers are typically large molecules (e.g., chelates), which may impact freezing. However, the concentration of stabilizers is typically < 10 ppm by weight, which would not alter bulk water properties substantially. In fact, the equilibrium melting temperature suppression by this concentration is negligible (in the order of ~10^-3^ °C), assuming a molar heat of fusion of 6.02 kJ mol^-1^. Hence, even though H_2_O_2_ can contain stabilizers, the low concentration means it may still prove useful for droplet freezing assays and further aid in reducing experimental errors due to background contaminants in test water.

Nonetheless, additional study is necessary to apply H_2_O_2_ to simulate atmospheric immersion freezing. Caution should be given on physicochemical modification of INPs by H_2_O_2_ especially because residual H_2_O_2_ may remain in the water and destroy INPs in the analyte when it is suspended in the H_2_O_2_ treated water. Carefully assessing residual H_2_O_2_ quantities through UV-spectrophotometry (λ = 628 nm, Sunil and Narayana, 2008) and other complementary techniques (e.g., **Sect. 3.3**) after boiling at 100 °C and exposing to full spectra UV treatment for 1 hour is important to optimizing the H_2_O_2_ concentration to recreate homogeneous freezing and ascertain whether H_2_O_2_ treatment suppresses the ice nucleation efficiency of any test INPs present.

**SI Sect. S7:** Data Used to Generate Figures

The details of the experimental data used for generating Figs. 1, 2, 4, S1, S2, and S3, as well as the data used for Modules 2 (Figs. 2 and 3) and Module 3 (Figs. 4 and 5), are provided in a separate data file, entitled *SI_Sect_S7_data*.

# **References**

Cory, K. M.: *Immersion freezing of non-proteinaceous biological aerosol proxies and arctic ambient particles*, M.S. thesis, West Texas A&M University, Canyon, TX, USA, available at *https://wtamu-ir.tdl.org/handle/11310/227* (last accessed on March 26, 2022), pp 66, 2019.

Hill, T. C. J., DeMott, P. J., Tobo, Y., Fröhlich-Nowoisky, J., Moffett, B. F., Franc, G. D., and Kreidenweis, S. M.: Sources of organic ice nucleating particles in soils, *Atmos. Chem. Phys., 16,* 7195–7211, https://doi.org/10.5194/acp-16-7195-2016, 2016.

Hiranuma, N., Augustin-Bauditz, S., Bingemer, H., Budke, C., Curtius, J., Danielczok, A., Diehl, K., Dreischmeier, K., Ebert, M., Frank, F., Hoffmann, N., Kandler, K., Kiselev, A., Koop, T., Leisner, T., Möhler, O., Nillius, B., Peckhaus, A., Rose, D., Weinbruch, S., Wex, H., Boose, Y., DeMott, P. J., Hader, J. D., Hill, T. C. J., Kanji, Z. A., Kulkarni, G., Levin, E. J. T., McCluskey, C. S., Murakami, M., Murray, B. J., Niedermeier, D., Petters, M. D., O'Sullivan, D., Saito, A., Schill, G. P., Tajiri, T., Tolbert, M. A., Welti, A., Whale, T. F., Wright, T. P., and Yamashita, K.: A comprehensive laboratory study on the immersion freezing behavior of illite NX particles: a comparison of 17 ice nucleation measurement techniques, *Atmos. Chem. Phys., 15,* 2489–2518, https://doi.org/10.5194/acp-15-2489-2015, 2015.

Hiranuma, N., Adachi, K., Bell, D. M., Belosi, F., Beydoun, H., Bhaduri, B., Bingemer, H., Budke, C., Clemen, H.-C., Conen, F., Cory, K. M., Curtius, J., DeMott, P. J., Eppers, O., Grawe, S., Hartmann, S., Hoffmann, N., Höhler, K., Jantsch, E., Kiselev, A., Koop, T., Kulkarni, G., Mayer, A., Murakami, M., Murray, B. J., Nicosia, A., Petters, M. D., Piazza, M., Polen, M., Reicher, N., Rudich, Y., Saito, A., Santachiara, G., Schiebel, T., Schill, G. P., Schneider, J., Segev, L., Stopelli, E., Sullivan, R. C., Suski, K., Szakáll, M., Tajiri, T., Taylor, H., Tobo, Y., Ullrich, R., Weber, D., Wex, H., Whale, T. F., Whiteside, C. L., Yamashita, K., Zelenyuk, A., and Möhler, O.: A comprehensive characterization of ice nucleation by three different types of cellulose particles immersed in water, Atmos. Chem. Phys., 19, 4823–4849, https://doi.org/10.5194/acp-19-4823-2019, 2019.

Koop T., Luo B. P., Tsias A., and Peter T.: Water activity as the determinant for homogeneous ice nucleation in aqueous solutions, *Nature, 406,* 611–614, 2000.

Koop, T., and Murray, B. J.: A physically constrained classical description of the homogeneous nucleation of ice in water, *Chemical Physics., 145,* 1–12, 2016.

Lunak, S., Sedlak, P.: Photoinitiated reactions of hydrogen peroxide in the liquid phase, *Photochem. Photobiol. A: Chem., 68,* 1–33, 1992.

Oguni, M., and Angell, C. A.: Heat capacities of H_2_O+H_2_O_2_, and H _2_O+N_2_H_4_, binary solutions: Isolation of a singular component for C_p_ of supercooled water, Journal of Chemical Physics, 73, 1948–1954, 1980.

Perkins, R. J., Gillette, S. M., Hill, T. C. J., and DeMott, P. J.: The labile nature of ice nucleation by Arizona Test Dust. *ACS Earth Space Chem., 4,* 133–141, 2020.

Schiebel, T.: *Ice nucleation activity of soil dust aerosols*, Ph.D. thesis, Karlsruhe Institute of Technology, Karlsruhe, Germany, available at *https://doi.org/10.5445/IR/1000076327* (last accessed on March 26, 2022), pp 131, 2017.

Sunil, K., and Narayana, B.: Spectrophotometric determination of hydrogen peroxide in water and cream samples, *Bull. Environ. Contam. Toxicol., 81,* 422-426, 2008.

Vali, G.: Quantitative evaluation of experimental results on the heterogeneous freezing nucleation of supercooled liquids*, J. Atmos. Sci., 28,* 402–409, 1971.

Vepuri, H. S. K., Rodriguez, C. A., Georgakopoulos, D. G., Hume, D., Webb, J., Mayer, G. D., and Hiranuma, N.: Ice-nucleating particles in precipitation samples from the Texas Panhandle, Atmos. Chem. Phys., 21, 4503–4520, https://doi.org/10.5194/acp-21-4503-2021, 2021.

Wex, H., Augustin-Bauditz, S., Boose, Y., Budke, C., Curtius, J., Diehl, K., Dreyer, A., Frank, F., Hartmann, S., Hiranuma, N., Jantsch, E., Kanji, Z. A., Kiselev, A., Koop, T., Möhler, O., Niedermeier, D., Nillius, B., Rösch, M., Rose, D., Schmidt, C., Steinke, I., and Stratmann, F.: Intercomparing different devices for the investigation of ice nucleating particles using Snomax^®^ as test substance, *Atmos. Chem. Phys., 15,* 1463–1485, https://doi.org/10.5194/acp-15-1463-2015, 2015.
